# Author Correction: Study of 300,486 individuals identifies 148 independent genetic loci influencing general cognitive function

**DOI:** 10.1038/s41467-019-10160-w

**Published:** 2019-05-01

**Authors:** Gail Davies, Max Lam, Sarah E. Harris, Joey W. Trampush, Michelle Luciano, W. David Hill, Saskia P. Hagenaars, Stuart J. Ritchie, Riccardo E. Marioni, Chloe Fawns-Ritchie, David C. M. Liewald, Judith A. Okely, Ari V. Ahola-Olli, Catriona L. K. Barnes, Lars Bertram, Joshua C. Bis, Katherine E. Burdick, Andrea Christoforou, Pamela DeRosse, Srdjan Djurovic, Thomas Espeseth, Stella Giakoumaki, Sudheer Giddaluru, Daniel E. Gustavson, Caroline Hayward, Edith Hofer, M. Arfan Ikram, Robert Karlsson, Emma Knowles, Jari Lahti, Markus Leber, Shuo Li, Karen A. Mather, Ingrid Melle, Derek Morris, Christopher Oldmeadow, Teemu Palviainen, Antony Payton, Raha Pazoki, Katja Petrovic, Chandra A. Reynolds, Muralidharan Sargurupremraj, Markus Scholz, Jennifer A. Smith, Albert V. Smith, Natalie Terzikhan, Anbupalam Thalamuthu, Stella Trompet, Sven J. van der Lee, Erin B. Ware, B. Gwen Windham, Margaret J. Wright, Jingyun Yang, Jin Yu, David Ames, Najaf Amin, Philippe Amouyel, Ole A. Andreassen, Nicola J. Armstrong, Amelia A. Assareh, John R. Attia, Deborah Attix, Dimitrios Avramopoulos, David A. Bennett, Anne C. Böhmer, Patricia A. Boyle, Henry Brodaty, Harry Campbell, Tyrone D. Cannon, Elizabeth T. Cirulli, Eliza Congdon, Emily Drabant Conley, Janie Corley, Simon R. Cox, Anders M. Dale, Abbas Dehghan, Danielle Dick, Dwight Dickinson, Johan G. Eriksson, Evangelos Evangelou, Jessica D. Faul, Ian Ford, Nelson A. Freimer, He Gao, Ina Giegling, Nathan A. Gillespie, Scott D. Gordon, Rebecca F. Gottesman, Michael E. Griswold, Vilmundur Gudnason, Tamara B. Harris, Annette M. Hartmann, Alex Hatzimanolis, Gerardo Heiss, Elizabeth G. Holliday, Peter K. Joshi, Mika Kähönen, Sharon L. R. Kardia, Ida Karlsson, Luca Kleineidam, David S. Knopman, Nicole A. Kochan, Bettina Konte, John B. Kwok, Stephanie Le Hellard, Teresa Lee, Terho Lehtimäki, Shu-Chen Li, Christina M. Lill, Tian Liu, Marisa Koini, Edythe London, Will T. Longstreth, Oscar L. Lopez, Anu Loukola, Tobias Luck, Astri J. Lundervold, Anders Lundquist, Leo-Pekka Lyytikäinen, Nicholas G. Martin, Grant W. Montgomery, Alison D. Murray, Anna C. Need, Raymond Noordam, Lars Nyberg, William Ollier, Goran Papenberg, Alison Pattie, Ozren Polasek, Russell A. Poldrack, Bruce M. Psaty, Simone Reppermund, Steffi G. Riedel-Heller, Richard J. Rose, Jerome I. Rotter, Panos Roussos, Suvi P. Rovio, Yasaman Saba, Fred W. Sabb, Perminder S. Sachdev, Claudia L. Satizabal, Matthias Schmid, Rodney J. Scott, Matthew A. Scult, Jeannette Simino, P. Eline Slagboom, Nikolaos Smyrnis, Aïcha Soumaré, Nikos C. Stefanis, David J. Stott, Richard E. Straub, Kjetil Sundet, Adele M. Taylor, Kent D. Taylor, Ioanna Tzoulaki, Christophe Tzourio, André Uitterlinden, Veronique Vitart, Aristotle N. Voineskos, Jaakko Kaprio, Michael Wagner, Holger Wagner, Leonie Weinhold, K. Hoyan Wen, Elisabeth Widen, Qiong Yang, Wei Zhao, Hieab H. H. Adams, Dan E. Arking, Robert M. Bilder, Panos Bitsios, Eric Boerwinkle, Ornit Chiba-Falek, Aiden Corvin, Philip L. De Jager, Stéphanie Debette, Gary Donohoe, Paul Elliott, Annette L. Fitzpatrick, Michael Gill, David C. Glahn, Sara Hägg, Narelle K. Hansell, Ahmad R. Hariri, M. Kamran Ikram, J. Wouter Jukema, Eero Vuoksimaa, Matthew C. Keller, William S. Kremen, Lenore Launer, Ulman Lindenberger, Aarno Palotie, Nancy L. Pedersen, Neil Pendleton, David J. Porteous, Katri Räikkönen, Olli T. Raitakari, Alfredo Ramirez, Ivar Reinvang, Igor Rudan, Dan Rujescu, Reinhold Schmidt, Helena Schmidt, Peter W. Schofield, Peter R. Schofield, John M. Starr, Vidar M. Steen, Julian N. Trollor, Steven T. Turner, Cornelia M. Van Duijn, Arno Villringer, Daniel R. Weinberger, David R. Weir, James F. Wilson, Anil Malhotra, Andrew M. McIntosh, Catharine R. Gale, Sudha Seshadri, Thomas H. Mosley, Jan Bressler, Todd Lencz, Ian J. Deary

**Affiliations:** 1 Centre for Cognitive Ageing and Cognitive Epidemiology, Department of Psychology, School of Philosophy, Psychology and Language Sciences, The University of Edinburgh, Edinburgh, EH8 9JZ UK; 2 Institute of Mental Health, Singapore, 539747 Singapore; 3 Medical Genetics Section, Centre for Genomic & Experimental Medicine, MRC Institute of Genetics & Molecular Medicine, University of Edinburgh, Western General Hospital, Edinburgh, EH4 2XU UK; 4 BrainWorkup, LLC, Los Angeles, 90033 CA USA; 5 Department of Psychiatry and Behavioral Sciences, Keck School of Medicine, University of Southern California, Los Angeles, 90033 CA USA; 6 Social, Genetic and Developmental Psychiatry Centre, Institute of Psychiatry, Psychology & Neuroscience, King’s College London, De Crespigny Park, Denmark Hill, London, SE5 8AF UK; 7 Research Centre of Applied and Preventive Cardiovascular Medicine, University of Turku, Turku, 20520 Finland; 8 Department of Internal Medicine, Satakunta Central Hospital, Pori, 28100 Finland; 9 Centre for Global Health Research, Usher Institute of Population Health Sciences and Informatics, University of Edinburgh, Teviot Place, Edinburgh, EH8 9AG Scotland; 10 Max Planck Institute for Molecular Genetics, Berlin, 14195 Germany; 11 Cardiovascular Health Research Unit, Department of Medicine, University of Washington, Seattle, 98101 Washington USA; 12 Department of Psychiatry, Icahn School of Medicine at Mount Sinai, New York, 10029 NY USA; 13 Mental Illness Research, Education, and Clinical Center (VISN 3), James J. Peters VA Medical Center, Bronx, 10468 NY USA; 14 Department of Psychiatry, Brigham and Women’s Hospital, Harvard Medical School, Boston, 02115 MA USA; 15 NORMENT, K.G. Jebsen Centre for Psychosis Research, Department of Clinical Science, University of Bergen, Bergen, 5021 Norway; 16 Dr. Einar Martens Research Group for Biological Psychiatry, Center for Medical Genetics and Molecular Medicine, Haukeland University Hospital, Bergen, 5020 Norway; 17 Center for Psychiatric Neuroscience, Feinstein Institute for Medical Research, Manhasset, 11030 NY USA; 18 Department of Medical Genetics, Oslo University Hospital, University of Bergen, Oslo, 0424 Norway; 19 Department of Psychology, University of Oslo, Oslo, 0373 Norway; 20 Division of Mental Health and Addiction, Oslo University Hospital, Oslo, 0315 Norway; 21 Department of Psychology, University of Crete, Crete, GR-74100 Greece; 22 Department of Psychiatry, University of California, San Diego, 92093 CA USA; 23 Center for Behavior Genetics of Aging, University of California, San Diego, 92093 CA USA; 24 Medical Research Council Human Genetics Unit, Institute of Genetics and Molecular Medicine, University of Edinburgh, Edinburgh, EH4 2XU UK; 25 Generation Scotland, Centre for Genomic and Experimental Medicine, University of Edinburgh, Edinburgh, EH4 2XU UK; 26 Department of Neurology, Clinical Division of Neurogeriatrics, Medical University of Graz, Graz, 8036 Austria; 27 Institute of Medical Informatics Statistics and Documentation, Medical University of Graz, Graz, 8036 Austria; 28 Department of Epidemiology, Erasmus University Medical Center, Rotterdam, 3015 The Netherlands; 29 Department of Radiology and Nuclear Medicine, Erasmus University Medical Center, Rotterdam, 3015 The Netherlands; 30 Department of Neurology, Erasmus University Medical Center, Rotterdam, xxxxxx The Netherlands; 31 Department of Medical Epidemiology and Biostatistics, Karolinska Institutet, Stockholm, SE-171 77 Sweden; 32 Department of Psychiatry, Yale University School of Medicine, New Haven, 06511 CT USA; 33 Department of Psychology and Logopedics, Faculty of Medicine, University of Helsinki, Helsinki, 00014 Finland; 34 Helsinki Collegium for Advanced Studies, University of Helsinki, Helsinki, 00014 Finland; 35 Department of Psychiatry and Psychotherapy, University of Cologne, Cologne, D-50937 Germany; 36 Department of Biostatistics, Boston University School of Public Health, Boston, 02118 MA USA; 37 Centre for Healthy Brain Ageing, School of Psychiatry, University of New South Wales, Sydney, 2031 Australia; 38 Neuroimaging, Cognition & Genomics (NICOG) Centre, School of Psychology and Discipline of Biochemistry, National University of Ireland Galway, Galway, H91 TK33 Ireland; 39 Medical Research Institute and Faculty of Health, University of Newcastle, New South Wa0les, 2308 Australia; 40 Institute for Molecular Medicine Finland (FIMM), University of Helsinki, Helsinki, FI-00014 Finland; 41 Centre for EpidemiologyDivision of Population Health, Health Services Research & Primary Care, The University of Manchester, Manchester, M13 9PL UK; 42 Department of Epidemiology and Biostatistics, School of Public Health, Imperial College London, London, W2 1PG UK; 43 Department of Psychology, University of California Riverside, Riverside, 92521 CA USA; 44 University of Bordeaux, Bordeaux Population Health Research Center, INSERM UMR 1219, F-33000 Bordeaux, France; 45 Institute for Medical Informatics, Statistics and Epidemiology, University of Leipzig, Leipzig, 04107 Germany; 46 LIFE—Leipzig Research Center for Civilization Diseases, University of Leipzig, Leipzig, 04107 Germany; 47 Department of Epidemiology, School of Public Health, University of Michigan, Ann Arbor, MI 48109 USA; 48 Survey Research Center, Institute for Social Research, University of Michigan, Ann Arbor, MI 48104 USA; 49 Icelandic Heart Association, Kopavogur, IS-201 Iceland; 50 University of Iceland, Reykjavik, 101 Iceland; 51 Department of Respiratory Medicine, Ghent University Hospital, De Pintelaan 185, 9000 Ghent, Belgium; 52 Section of Gerontology and Geriatrics, Department of Internal Medicine, Leiden University Medical Center, Leiden, 2333 The Netherlands; 53 Department of Medicine, Division of Geriatrics, University of Mississippi Medical Center, Jackson, 39216 MS USA; 54 Queensland Brain Institute, University of Queensland, Brisbane, 4072 Australia; 55 Centre for Advanced Imaging, University of Queensland, Brisbane, 4072 Australia; 56 Rush Alzheimer’s Disease Center, Rush University Medical Center, Chicago, 60612 IL USA; 57 Department of Neurological Sciences, Rush University Medical Center, Chicago, 60612 IL USA; 58 National Ageing Research Institute, Royal Melbourne Hospital, Victoria, 3052 Australia; 59 Academic Unit for Psychiatry of Old Age, University of Melbourne, St George’s Hospital, Kew, 3010 Australia; 60 Univ. Lille, Inserm, CHU Lille, Institut Pasteur de Lille, U1167-LabEx DISTALZ, F-59000 Lille, France; 61 Norwegian Centre for Mental Disorders Research (NORMENT), Institute of Clinical Medicine, University of Oslo, Oslo, 0372 Norway; 62 Mathematics and Statistics, Murdoch University, Perth, 6150 Australia; 63 Hunter Medical Research Institute and Faculty of Health, University of Newcastle, New South Wales, 2305 Australia; 64 Department of NeurologyBryan Alzheimer’s Disease Research Center, and Center for Genomic and Computational Biology, Duke University Medical Center, Durham, 27708 NC USA; 65 Psychiatry and Behavioral Sciences, Division of Medical Psychology, and Department of Neurology, Duke University Medical Center, Durham, 27708 NC USA; 66 Department of Psychiatry, Johns Hopkins University School of Medicine, MD Baltimore, 21287 USA; 67 McKusick-Nathans Institute of Genetic Medicine, Johns Hopkins University School of Medicine, MD Baltimore, 21287 USA; 68 Institute of Human Genetics, University of Bonn, Bonn, 53113 Germany; 69 Department of Genomics, Life and Brain Center, University of Bonn, Bonn, 53113 Germany; 70 Departments of Behavioral Sciences, Rush University Medical Center, Chicago, 60612 IL USA; 71 Dementia Centre for Research Collaboration, University of New South Wales, Sydney, 2031 NSW Australia; 72 Department of Psychology, Yale University, New Haven, 06520 CT USA; 73 Human Longevity Inc, Durham, 27709 NC USA; 74 UCLA Semel Institute for Neuroscience and Human Behavior, Los Angeles, 90024 CA USA; 75 23andMe, Inc., Mountain View, 94041 CA USA; 76 Department of Cognitive Science, University of California, San Diego, La Jolla, 92093 CA USA; 77 Department of Neurosciences, University of California, San Diego, La Jolla, 92093 CA USA; 78 Department of Radiology, University of California, San Diego, La Jolla, 92093 CA USA; 79 MRC-PHE Centre for Environment, School of Public Health, Imperial College London, London, W2 1PG UK; 80 Department of Psychology, Virginia Commonwealth University, Richmond, 23284 VA USA; 81 Clinical and Translational Neuroscience Branch, Intramural Research Program, National Institute of Mental Health, National Institute of Health, Bethesda, 20892 MD USA; 82 National Institute for Health and Welfare, Helsinki, FI-00271 Finland; 83 Department of General Practice and Primary Health Care, University of Helsinki, Helsinki, 00290 Finland; 84 Helsinki University Central Hospital, Unit of General Practice, Helsinki, FI-00029 Finland; 85 Folkhälsan Research Centre, Helsinki, 2018 Finland; 86 Robertson Centre for Biostatistics, University of Glasgow, Glasgow, G12 8QQ United Kingdom; 87 Department of Psychiatry, Martin Luther University of Halle-Wittenberg, Halle, 06108 Germany; 88 Virginia Institute for Psychiatric and Behavioral Genetics, Virginia Commonwealth University, Richmond, 23298 VA USA; 89 QIMR Berghofer Medical Research Institute, Brisbane, 4029 Australia; 90 Department of Neurology, Johns Hopkins University School of Medicine, Baltimore, 21287 MD USA; 91 Department of Epidemiology, Johns Hopkins Bloomberg School of Public Health, Baltimore, 21205 MD USA; 92 Department of Data Science, University of Mississippi Medical Center, Jackson, 39216 MS USA; 93 Intramural Research Program National Institutes on Aging, National Institutes of Health, Bethesda, 20892 MD USA; 94 Department of Psychiatry, National and Kapodistrian University of Athens Medical School, Eginition Hospital, Athens, 11528 Greece; 95 University Mental Health Research Institute, Athens, GR-156 01 Greece; 96 Neurobiology Research Institute, Theodor-Theohari Cozzika Foundation, Athens, 11521 Greece; 97 Department of Epidemiology, University of North Carolina Gillings School of Global Public Health, Chapel Hill, 27599 NC USA; 98 Department of Clinical Physiology, Tampere University Hospital, and Finnish Cardiovascular Research Center, Tampere, FI-33014 Finland; 99 Faculty of Medicine and Life Sciences, University of Tampere, Tampere, 33521 Finland; 100 Department of Clinical Physiology, Finnish Cardiovascular Research Center—Tampere, Faculty of Medicine and Life Sciences, University of Tampere, Tampere, 33014 Finland; 101 Department of Psychiatry Medical Faculty, University of Cologne, Cologne, 50923 Germany; 102 Department of Psychiatry and Psychotherapy, University of Bonn, Bonn, 53127 Germany; 103 German Center for Neurodegenerative Diseases (DZNE), Bonn, 53127 Germany; 104 Department for Neurodegenerative Diseases and Geriatric Psychiatry, University of Bonn, Bonn, 53127 Germany; 105 Department of Neurology, Mayo Clinic, Rochester, 55905 MN USA; 106 Neuropsychiatric Institute, Prince of Wales Hospital, Sydney, 2031 Australia; 107 Brain and Mind Centre—The University of Sydney, Camperdown, NSW 2050 Australia; 108 School of Medical Sciences, University of New South Wales, Sydney, 2052 Australia; 109 Department of Clinical Chemistry, Fimlab Laboratories, Finnish Cardiovascular Research Center—Tampere, Faculty of Medicine and Life Sciences, University of Tampere, Tampere, 33014 Finland; 110 Department of Clinical Chemistry, Finnish Cardiovascular Research Center—Tampere, Faculty of Medicine and Life Sciences, University of Tampere, Tampere, 33014 Finland; 111 Max Planck Institute for Human Development, Berlin, 14195 Germany; 112 Technische Universität Dresden, Dresden, 01187 Germany; 113 Genetic and Molecular Epidemiology Group, Lübeck Interdisciplinary Platform for Genome Analytics, Institutes of Neurogenetics & Cardiogenetics, University of Lübeck, Lübeck, Germany; 114 Department of Neurology, School of Medicine, University of Washington, Seattle, 98195-6465 WA USA; 115 Department of Epidemiology, University of Washington, Seattle, 98195 WA USA; 116 Department of Neurology and Psychiatry, University of Pittsburgh, Pittsburgh, 15213 PA USA; 117 Institute of Social Medicine, Occupational Health and Public Health (ISAP), University of Leipzig, Leipzig, 04103 Germany; 118 Department of Biological and Medical Psychology, University of Bergen, Bergen, 5009 Norway; 119 K. G. Jebsen Center for Neuropsychiatry, University of Bergen, Bergen, N-5009 Norway; 120 Umeå Center for Functional Brain Imaging (UFBI), Umeå University, Umeå, SE-901 87 Sweden; 121 Department of Statistics, USBE Umeå University, S-907 97 Umeå, Sweden; 122 Institute for Molecular Bioscience, University of Queensland, Brisbane, 4072 Australia; 123 The Institute of Medical Sciences, Aberdeen Biomedical Imaging Centre, University of Aberdeen, Aberdeen, AB25 2ZD UK; 124 Division of Brain Sciences, Department of Medicine, Imperial College, London, SW7 2AZ UK; 125 Department of Radiation Sciences, Umeå University, Umeå, SE-901 87 Sweden; 126 Department of Integrative Medical Biology, Umeå University, Umeå, SE-901 87 Sweden; 127 Centre for Integrated Genomic Medical Research, Institute of Population Health, University of Manchester, Manchester, M13 9PT UK; 128 Karolinska Institutet, Aging Research Center, Stockholm University, Stockholm, SE-113 30 Sweden; 129 Department of Psychology, School of Philosophy, Psychology and Language Sciences, The University of Edinburgh, Edinburgh, EH8 9JZ UK; 130 Gen-Info LLC, Zagreb, 10000 Croatia; 131 Faculty of Medicine, University of Split, Split, 21000 Croatia; 132 Department of Psychology, Stanford University, Palo Alto, 94305-2130 CA USA; 133 Deparment of Health Services, University of Washington, Seattle, 98195-7660 WA USA; 134 Kaiser Permanente Washington Health Research Institute, Seattle, 98101 WA USA; 135 Department of Developmental Disability Neuropsychiatry, School of Psychiatry, University of New South Wales, Sydney, 2052 Australia; 136 Department of Psychological and Brain Sciences, Indiana University, Bloomington, IN 47405-7007 USA; 137 Institute for Translational Genomics and Population Sciences, Los Angeles Biomedical Research Institute at Harbor-UCLA Medical Center, Torrance, CA 90502 USA; 138 Department of Pediatrics, Harbor-UCLA Medical Center, Torrance, 90509 CA USA; 139 Department of Genetics and Genomic Science and Institute for Multiscale Biology, Icahn School of Medicine at Mount Sinai, New York, 10029 NY USA; 140 Mental Illness Research, Education, and Clinical Center (VISN 2), James J. Peters VA Medical Center, Bronx, 10468 NY USA; 141 Institute of Molecular Biology and Biochemistry, Centre for Molecular Medicine, Medical University of Graz, Graz, 8036 Austria; 142 Robert and Beverly Lewis Center for Neuroimaging, University of Oregon, Eugene, 97403 OR USA; 143 Department of Neurology, Boston University School of Medicine, Boston, 02118 MA USA; 144 The National Heart, Lung, and Blood Institute’s Framingham Heart Study, Framingham, 01702-5827 MA USA; 145 Department of Medical Biometry, Informatics and Epidemiology, University Hospital, Bonn, D-53012 Germany; 146 Laboratory of NeuroGenetics, Department of Psychology & Neuroscience, Duke University, Durham, 27708-0086 NC USA; 147 Department of Molecular Epidemiology, Leiden University Medical Center, Leiden, 2333 The Netherlands; 148 Institute of Cardiovascular and Medical Sciences, University of Glasgow, Glasgow, G12 8QQ United Kingdom; 149 Lieber Institute for Brain Development, Johns Hopkins University Medical Campus, Baltimore, 21205 MD USA; 150 Department of Hygiene and Epidemiology, University of Ioannina Medical School, Ioannina, 45110 Greece; 151 Department of Public Health, University Hospital of Bordeaux, Bordeaux, 33076 France; 152 Department of Internal Medicine, Erasmus Medical Center, Rotterdam, 3015 The Netherlands; 153 Campbell Family Mental Health Institute, Centre for Addiction and Mental Health, University of Toronto, Toronto, M5T 1L8 Canada; 154 Department of Public Health, University of Helsinki, Helsinki, 00014 Finland; 155 Department of Radiology, Erasmus MC, Rotterdam, 3015 The Netherlands; 156 Department of Psychiatry and Behavioral Sciences, Faculty of Medicine, University of Crete, Heraklion, GR-71003 Greece; 157 Human Genetics Center, School of Public Health, University of Texas Health Science Center at Houston, Houston, 77030 TX USA; 158 Human Genome Sequencing Center, Baylor College of Medicine, Houston, 77030-3411 TX USA; 159 Neuropsychiatric Genetics Research Group, Department of Psychiatry and Trinity College Institute of Neuroscience, Trinity College Dublin, Dublin, DO2 AY89 Ireland; 160 Center for Translational and Systems Neuroimmunology, Department of Neurology, Columbia University Medical Center, New York, 10032 NY USA; 161 Program in Medical and Population Genetics, Broad Institute, Cambridge, 02142 MA USA; 162 Department of Neurology, University Hospital of Bordeaux, Bordeaux, 33000 France; 163 Department of Global Health, University of Washington, Seattle, 98104 WA USA; 164 Department of Cardiology, Leiden University Medical Center, Leiden, 2333 The Netherlands; 165 Institute for Behavioral Genetics, University of Colorado, Boulder, 80309 CO USA; 166 Wellcome Trust Sanger Institute, Wellcome Trust Genome Campus, Cambridge, CB10 1SA UK; 167 Department of Medical Genetics, University of Helsinki and University Central Hospital, Helsinki, 00014 Finland; 168 Division of Neuroscience and Experimental Psychology, School of Biological Sciences, Manchester Academic Health Science Centre, and Manchester Medical School, Institute of Brain, Behaviour, and Mental Health, University of Manchester, Manchester, M13 9PL UK; 169 Department of Clinical Physiology and Nuclear Medicine, Turku University Hospital, Turku, 20520 Finland; 170 School of Medicine and Public Health, University of Newcastle, New South Wales, 2308 Australia; 171 Neuroscience Research Australia, Sydney, 2031 Australia; 172 Faculty of Medicine, University of New South Wales, Sydney, 2052 Australia; 173 Alzheimer Scotland Dementia Research Centre, University of Edinburgh, Edinburgh, EH8 9JZ UK; 174 Division of Nephrology and Hypertension, Department of Internal Medicine, Mayo Clinic, Rochester, MN 55905 USA; 175 Max Planck Institute for Human Cognitive and Brain Sciences, Leipzig, 04103 Germany; 176 Day Clinic for Cognitive Neurology, University Hospital Leipzig, Leipzig, 04103 Germany; 177 Division of Psychiatry Research, Zucker Hillside Hospital, Glen Oaks, 11004 NY USA; 178 Department of Psychiatry, Hofstra Northwell School of Medicine, Hempstead, 11549 NY USA; 179 Division of Psychiatry, University of Edinburgh, Edinburgh, EH10 5HF UK; 180 MRC Lifecourse Epidemiology Unit, University of Southampton, Southampton, SO16 6YD UK; 181 Glenn Biggs Institute for Alzheimer’s and Neurodegenerative Diseases, University of Texas Health Sciences Center, San Antonio, 78229 TX USA

**Keywords:** Genome-wide association studies, Quantitative trait, Cognitive neuroscience, Genetics of the nervous system

Correction to: *Nature Communications* 10.1038/s41467-018-04362-x, published online 29 May 2018.

Christina M. Lill, who contributed to analysis of data, was inadvertently omitted from the author list in the originally published version of this article. This has now been corrected in both the PDF and HTML versions of the article.

