# Supplementary material for: Study of 300,486 individuals identifies 148 independent genetic loci influencing general cognitive function
Source: Nat Commun. 2018 May 29;9:2098. doi: 10.1038/s41467-018-04362-x (PMC5974083; doi:10.1038/s41467-018-04362-x)
Supplement: Supplementary file 1 — Supplementary Information [file 41467_2018_4362_MOESM1_ESM.pdf]

Supplementary Information

Study of 300,486 individuals identifies 148 independent genetic loci influencing general cognitive function

Davies et al.

Supplementary Notes

Supplementary Note 1

Phenotype Descriptions

UK Biobank

Verbal-numerical reasoning test. At baseline, a sub-sample of UK Biobank participants were administered the verbal-numerical reasoning test (n = 187,211; referred to as the ‘fluid intelligence test’ by UK Biobank). Participants were asked 13 multiple-choice questions that assessed verbal and numerical problem solving. The score was the number of questions answered correctly in two minutes. This test has been shown to have adequate test-retest reliability (r = 0.65)[1] and internal consistency (Cronbach α = 0.62)[2]. The verbal-numerical reasoning test was also administered to three sub-samples of participants at, the first repeat assessment visit (n = 20,115), the imaging visit (n = 15,750) or during a web-based cognitive assessment (n = 123,665). In the web-based version of this test there was an additional question, thus the maximum score was 14.

Four samples of UK Biobank participants with verbal-numerical reasoning scores were used in the current analysis. The first sample (VNR Assessment Centre) consists of UK Biobank participants with genome-wide genotyping data, following quality control procedures, who also completed the verbal-numerical reasoning test at baseline (n = 107,586). The second sample (VNR T2) consists of participants who did not complete the verbal-numerical reasoning test at baseline but did complete this test at the first repeat assessment visit in assessment centres (n = 11,123). The third sample (VNR MRI) consists of participants who did not complete the verbal-numerical reasoning test at a previous testing occasion but did complete the test at the imaging visit in assessment centres (n = 3002). The fourth sample (VNR Web-Based) consists of UK Biobank participants with genome-wide

genotyping data who did not complete the verbal-numerical reasoning test at any assessment centre visit but did complete this test during the web-based cognitive assessment (n = 46,322). In this analysis, samples were analysed separately because there were differences in both the test questions and the testing environment.

Reaction time test. At the baseline UK Biobank assessment, 496,790 participants completed the reaction time test. Here, participants were presented with pairs of cards on the computer screen. The two cards could either be the same or different. If the two cards were identical, participants were to push a button box as quickly as possible. There were 12 trials in total. The first five were used as a practice. Of the remaining seven trials, four presented matching cards. The score is the mean time, in milliseconds, to correctly identify the matching cards in these four trials. While there are only a few trials, internal consistency is good (Cronbach $\alpha = 0.85$)[2]. For the current analysis, a sample of 330,069 UK Biobank participants with both scores on the reaction time test and genotyping data were used.

CHARGE Cohorts

3C-Dijon. Scores on the following cognitive ability tests were used to create the fluid-type general cognitive function component: Trail Making Test B (TMTB; time to complete part B – in seconds), Benton Visual Retention Test (BVRT; number of questions answered correctly), and Delayed Recall (Five Words Memory Test; number of words correctly remembered). The tests, the method of application and key references have been described in detail elsewhere[3]. The listwise N was 3,652. The Pearson correlations (*r*s) among the 3 tests ranged from -0.34 to 0.14 (mean of absolute values = 0.20). Principal components analysis was applied to these 3 tests. The first unrotated principal component (FUPC) accounted for 47.4% of the total test variance. Loadings on the FUPC were as follows: Delayed Recall = 0.49, BVRT = 0.77 and TMTB = -0.76

AgeCoDe. Scores on the following cognitive ability tests were used to create the fluid-type general cognitive function component: CERAD verbal fluency (number of animals named in one minute), CERAD immediate recall (sum of correctly remembered words over 3 trials; Range: 0-30), CERAD constructional praxis recall (four drawings previously copied should be reproduced from memory; range of performance ratings: 0-11). The tests, the method of application and key references have been described in detail elsewhere[4]. The listwise N was 617. The *r*s among the 3 tests ranged from 0.25 to 0.36 (mean of absolute values = 0.29). Principal component analysis was applied to these 3 tests. The FUPC accounted for 52.7% of the total test variance. Loadings on the FUPC were as follows: CERAD verbal fluency = 0.76, CERAD immediate recall = 0.76, CERAD constructional praxis recall = 0.66.

AGES. Scores on the following cognitive ability tests were used to create the fluid-type general cognitive function component: Digit Backward Test[5] (number of digit sequences remembered backwards), The Digit Symbol Substitution Test[5] (DSST; number of correct digit symbol matches made), California Verbal Learning Test[6] (CVLT), The Figure Comparison Test[7], The Modified Stroop Test[8] (Part III – word-colour interference), The Cambridge Neuropsychological Test Automated Battery (CANTAB) Spatial Working Memory[9]. The tests, the method of application and key references have also been described in detail elsewhere[10]. Absolute value of the *r*s among the 6 tests ranged from 0.21 to 0.77 (mean 0.39). Principal components analysis was applied to these 6 tests. The FUPC accounted for 50.1% of the total test variance. Loadings on the FUPC were as follows: Digits backward test = 0.64, DSST total correct cells = 0.87, CVLT 1-4 number of unique target words = 0.73, Figure Comparison total correct in 60 sec = 0.83, STROOP Part III time in sec = -0.62, CANTAB Spatial Working Memory total errors = -0.52.

Airwave. Scores on the following cognitive ability tests were used to create the fluid-type general cognitive function component: Reaction (Two-choice reaction time: time taken to select between a left or right response to match a left or right stimulus) , Pairing (Paired associate learning – episodic memory: requires the user to recall the positions of 7 hidden pictures), Quiz (Fluid intelligence: reasoning quiz presents multiple-choice questions, alternating between numerical and verbal problems), Stroop (Mild interference/Stroop interference; This test measured the effect on two-choice reaction time of increasing choice complexity), Number (Working Memory Test - Forward digit span). The listwise N was 9,908. Principal component analysis was applied to these 5 tests. The FUPC accounted for 33.2% of the total test variance. Loadings on the FUPC were as follows: Reaction = 0.61, Pairing = 0.45, Quiz = -0.58 Stroop = 0.70, Number = -0.50.

ARIC. Scores on the following cognitive ability tests were used to create the fluid-type general cognitive function component: Delayed Word Recall Test (total number of words recalled), Digit Symbol Substitution Test (total number of correct symbols), Word Fluency Test (sum of words produced beginning with the letters F, A, and S). The tests, the method of application and key references have been described in detail elsewhere[11-13]. The listwise N was 10,534. The *r*s among the 3 tests ranged from 0.24 to 0.43 (mean 0.34). Principal components analysis was applied to these 3 tests. The FUPC accounted for 56.1% of the total test variance. Loadings on the FUPC were as follows: Delayed Word Recall Test = 0.52, Digit Symbol Substitution Test = 0.63, Word Fluency Test = 0.58.

ASPS. Scores on the following cognitive ability tests were used to create the fluid-type general cognitive ability factor: Alterskonzentrations-Test (AKT; concentration test – time in s), Lern- und Gedächtnistest (LGT) (figural memory, total number of correct answers of two figural subtests), Lern- und Gedächtnistest (LGT) (verbal memory, total number of correct answers of three verbal subtests), Complex reaction time task (computerized task;

reaction time in ms), Digit Span – backward (length of highest correctly repeated digit list), Purdue Pegboard Test (visuo-practical skills; total number of correct elements in most difficult condition [assembly]), Trail Making Test – Version B (time in s). The tests, the method of application and key references have been described in detail elsewhere[14-19]. The listwise N was 748. The absolute *r*s among the 7 tests ranged from 0.125 to 0.528 (mean 0.327). Principal components analysis was applied to these 7 tests. The FUPC accounted for 42.6% of the total test variance. Loadings on the FUPC were as follows: Alterskonzentrations-Test = 0.534, figural memory (LGT) = 0.642, verbal memory (LGT) = 0.725, Complex reaction time task = 0.534, Digit Span = 0.595, Purdue Pegboard Test = 0.722, Trail Making Test-B = 0.774.

ASPS-Fam. Scores on the following cognitive ability tests were used to create the fluid-type general cognitive ability factor: Lern- und Gedächtnistest (LGT) (figural memory, total number of correct answers of two figural subtests) 1, Lern- und Gedächtnistest (LGT) (verbal memory, total number of correct answers of three verbal subtests) 2, Complex reaction time task (computerized task; reaction time in ms) 3, Digit Span – backward (length of highest correctly repeated digit list) 4, Purdue Pegboard Test (visuo-practical skills; total number of correct elements in most difficult condition [assembly]) 5, Trail Making Test – Version B (time in s) 6. The tests, the method of application and key references have been described in detail elsewhere[15-19]. The listwise N was 311. The absolute *r*s among the 6 tests ranged from 0.217 to 0.619 (mean 0.435). Principal components analysis was applied to these 6 tests. The FUPC accounted for 53.2% of the total test variance. Loadings on the FUPC were as follows: figural memory (LGT) 1 = 0.789, verbal memory (LGT) 2 = 0.809, Complex reaction time task 3 = 0.560, Digit Span 4 = 0.606, Purdue Pegboard Test 5 = 0.783, Trail Making Test-B 6 = 0.786.

BASEII. Scores on the following cognitive ability tests were used to a create fluid-type general cognitive function component: Spatial Working Memory (SWM; accuracy for location memory at set size 4); Wisconsin Card Sorting Test (WCST; % correct), which is assumed to index cognitive control or executive functioning; Mental Rotation (MR; sum of correct items in 7 minutes; max. 40); and Identical Pictures (IP; sum of correct answers in 80 seconds; max. 46 items), which is a measure of perceptual speed. The tests, test instructions, and key references have been described in detail elsewhere (SWM and WCST[20]; IP[21]; MR: test was designed for this study with the original versions as models[22]). The listwise N was 1,383. The *r*s among the 4 tests ranged from 0.13 to 0.29 (mean 0.22). Principal components analysis was applied to these 4 tests. The FUPC accounted for 41.2% of the total test variance. Loadings on the FUPC were as follows: SWM = 0.68, WCST = 0.60, MR = 0.62, IP = 0.67.

BATS. Scores on the following cognitive ability tests were used to create the general cognitive function component: Information (Multidimensional Aptitude Battery (MAB) subtest – number of correct answers in 7 minutes), Arithmetic (MAB subtest – number of correct answers in 7 minutes), Vocabulary (MAB subtest – number of correct answers in 7 minutes), Spatial (MAB subtest – number of correct answers in 7 minutes), Object Assembly (MAB subtest – number of correct answers in 7 minutes), Digit Symbol (WAIS-R subtest – number of correct answers in 90 seconds), Cambridge Contextual Reading Test (CCRT) (number of words correctly pronounced), Schonell Graded Word Reading Test (SGWRT) (number of words correctly pronounced). The tests, the method of application and key references have been described in detail elsewhere[23,24]. The listwise N was 2,253. The *r*s among the 8 tests ranged from 0.22 to 0.81 (mean of absolute values = 0.42). Principal component analysis was applied to these 8 tests. The FUPC accounted for 50.8% of the total test variance. Loadings on the FUPC were as follows: Information = 0.82, Arithmetic = 0.72,

Vocabulary = 0.78, Spatial = 0.61, Object Assembly = 0.66, Digit Symbol = 0.42, CCRT = 0.83, SGWRT = 0.79.

BETULA. Scores on the following cognitive ability tests were used to create the fluid-type general cognitive function component: Free Recall of Subject Performed Tasks (SPT; Immediate recall of 16 verb-noun combinations that were enacted during encoding), Fluency A (Generation during 1 minute of words beginning with the letter A), Block Design (from the Wechsler Adult Intelligence Scale), and Letter Digit (Letter Digit Substitution Test, 9 Letters/Digits; test time 1 min). The tests, the method of application and key references have been described in detail elsewhere[25,26]. The listwise N was 373. The *r*s among the 4 tests ranged from 0.26 to 0.57 (mean 0.38). Principal components analysis was applied to these 4 tests. The FUPC accounted for 53.9% of the total test variance. Loadings on the FUPC were as follows: SPT Sum = 0.71, Fluency A = 0.60, Block Design = 0.79, Letter Digit = 0.82.

CHS. Scores on the following cognitive ability tests were used to create the fluid-type general cognitive function component: Modified Mini-Mental Status Score (3MSE; total score up to 100), Digit Symbol Substitution Test (DSST; symbols correctly coded in 90 seconds), Benton Visual Retention Test (BVRT; number of designs of 10 correctly drawn after 10 second exposure with stimulus covered and immediate reproduction from memory tested), Trail Making Test A (TMTA; number of seconds to complete test), and Trail Making Test B (TMTB; number of seconds to complete test). The tests, the method of application and key references have been described in detail elsewhere[27,28]. The listwise N was 1,519. The absolute *r*s among the 5 tests ranged from 0.25 to 0.53 (mean 0.41). Principal components analysis was applied to these 5 tests. The FUPC accounted for 53.1% of the total test variance. Loadings on the FUPC were as follows: 3MSE = 0.69, DSST = 0.79, BVRT = 0.67, TMTA = 0.67, and TMTB = 0.81.

CROATIA-KORČULA. Scores on the following cognitive ability tests were used to create the fluid-type general cognitive function component: Digit Symbol Coding (DSC; sum of correct coding in 2 minutes), Standard Progressive Matrices (SPM; sum of total correct answers in 30 minutes), Verbal Fluency (FAS; sum of word produced beginning with the letters: F, A, S), Audio-Verbal Learning Test (AVLT_8; delayed recall – number of words remembered)[11,13,29]. The listwise N was 2,857. The rs among the 4 tests ranged from 0.19 - 0.49. Principal components analysis was applied to these 4 tests. The FUPC accounted for 48% of the total test variance. Loadings on the FUPC were as follows: DSC = 0.72, SPM = 0.77, FAS = 0.55, AVLT_8 = 0.68.

ELSA. Scores on the following cognitive ability tests were used to create the fluid-type general cognitive function component: Immediate Recall (number of correct words), Animal Naming (total named in one minute), Letter Cancellation Test (number correct), and Delayed Recall (number of correct words). The tests, the method of application and key references have been described in detail elsewhere[30]. The listwise N was 14,432. The *r*s among the 4 tests ranged from 0.28 to 0.70 (mean 0.40). Principal components analysis was applied to these 4 tests. The FUPC accounted for 55.97% of the total test variance. Loadings on the FUPC were as follows: Immediate Recall = 0.85, Animal Naming = 0.71, Letter Cancellation = 0.56, Delayed Recall = 0.85.

EPOZ. Scores on the following cognitive ability tests were used to create the fluid-type general cognitive function component: Stroop test (total time needed for the interference test), Letter Digit Substitution Test (total of correct answers), Word Fluency Test (number of correct animals), and the Word Learning Test (number of correct words on the delayed recall). The tests, the method of application and key references have been described in detail elsewhere[31]. The listwise N was 457. The absolute *r*s among the 4 tests ranged from 0.280 to 0.522 (mean of absolute values = 0.381). Principal component analysis was applied to these 4

tests. The FUPC accounted for 54.0% of the total test variance. Loadings on the FUPC were as follows: Stroop test = -0.759, Letter Digit Substitution Test = 0.819, Word Fluency Test = 0.745, Word Learning Test = 0.599.

ERF. Scores on the following cognitive ability tests were used to create the fluid-type general cognitive function component: Stroop 3 (time needed to complete Stroop color-word card), TMT-B (time needed to complete Trail Making Test part B), Phonemic Fluency (with D,A,T, number of words mentioned beginning with each letter, one minute each, sum of the three trials), 15-word Auditory Verbal Learning Test (AVLT-sum) (sum of immediate (5 iterations) and delayed recall (once)), WAIS Block Design test (n of correct answers, Wechsler scoring). The tests, the method of application and key references have been described in detail elsewhere[32]. The listwise N was 2,566. The absolute *r*s among the tests ranged from 0.607 to 0.331 (mean of absolute values = 0.46). Principal component analysis was applied to these 5 tests. The FUPC accounted for 58.3% of the total test variance. Loadings on the FUPC were as follows: Stroop 3 = -0.792, TMT-B = -0.843, Phonemic Fluency = 0.748, WAIS Block Design test = 0.650, AVLT-sum = 0.772.

FHS. Scores on the following cognitive ability tests were used to create the fluid-type general cognitive function component: Similarities, Trail Making Test B (TMTB), Logical Memory (sum of immediate and delayed recall scores), Visual Reproduction Memory (sum of immediate and delayed recall scores), Paired Associate Learning (sum of immediate and delayed recall scores), and Hooper Visual Organization test. The tests, the method of application and key references have been described in detail elsewhere[33]. Using an unrelated subset of 1,889 subjects, the absolute Pearson correlations among the 6 tests ranged from 0.33 to 0.55 (mean 0.42). Principal components analysis was applied to these 6 tests on the unrelated subset. The FUPC accounted for 52.6% of the total test variance. Loadings on the FUPC were as follows: Similarities = 0.40, TMTB = -0.43, Logical Memory = 0.37, Visual

Reproduction Memory = 0.44, Paired Associate Learning= 0.38, Hooper Visual Organization= 0.43.

Finnish Twin Cohort. Scores on the following cognitive ability tests were used to create the general cognitive function component: Vocabulary subtest from the Wechsler Intelligence Scale Revised (raw score – number correct; every other item from the standard administration included), Digit Span Backwards (raw score – number of trials remembered in reverse order), Trail Making Test B (time, log transformed), Stroop (inhibition, time, log transformed). The tests, the method of application and key references have been described in detail elsewhere[34,35]. The listwise N was 1,408. The *r*s among the 4 tests ranged from -0.19 to 0.40 (mean of absolute values = 0.29). Principal component analysis was applied to these 4 tests. The FUPC accounted for 46.7% of the total test variance. Loadings on the FUPC were as follows: Vocabulary = -0.56, Digit Span Backwards = -0.70, Trail Making Test B = 0.74, Stroop = 0.72. The FUPC was reversed prior to GWAS so that higher scores indicate better performance on the cognitive tasks.

Generation Scotland. Scores on the following cognitive ability tests were used to create the general cognitive function component: Wechsler Digit Symbol Substitution Task (number of digit symbol pairs made correctly), Wechsler Logical Memory Test (sum of immediate and delayed recall of one paragraph), Mill Hill Vocabulary Test, and the phonemic Verbal Fluency Test (sum of the words produced using the letters C, F, and L, each for one minute). The tests, the method of application and key references have been described in detail elsewhere[36,37]. The listwise N was 20,166. The rs among the 4 tests ranged from 0.06 to 0.40 (mean = 0.22). Principal component analysis was applied to these 4 tests. The FUPC accounted for 42% of the total test variance. Loadings on the FUPC were as follows: Digit Symbol = 0.58, Verbal Fluency = 0.71, Logical Memory = 0.63, Mill Hill = 0.66.

GENOA. Scores on the following five cognitive ability tests were used to create the fluid-type general cognitive ability factor: 1) Rey Auditory Verbal Learning Test (RAVLT) – Delayed Recall – Number of words recalled after a 30-minute delay, ranging from 0 to 15; 2) Weschler Adult Intelligence Scale-Revised (WAIS-R): Digit Symbol Substitution Test (DSST) – Processing Speed – Number of symbols correctly matched in 90 seconds; 3) Controlled Oral Word Association Test (COWA) – Verbal Fluency – Sum of words produced beginning with the letters F, A, and S; 4) Stroop Color-Word Test – Concentration – Number of correctly stated color words in 45 seconds; 5) Trail Making Test A – Visual Conceptual Tracking – natural logarithm of time to completion of the test in seconds, with a maximum completion time of 240 seconds.

The tests, the method of application and key references have been described in detail elsewhere [11,13,38-42]. The listwise N was 775. The absolute value of the *r*s among the 5 tests ranged from 0.210 to 0.572 (mean 0.397). Principal components analysis was applied to these 5 tests. The FUPC accounted for 52.4% of the total test variance. Loadings on the FUPC were as follows: RAVLT = 0.660, DSST = 0.859, COWA = 0.587, Stroop = 0.780, Trail A = -0.704.

Harmony. Scores on the following cognitive ability tests were used to create the general cognitive function component: Information (WAIS, 29 questions worth 1 point each), Verbal Fluency (the participant is asked to name as many animals as possible in 1 minute), WAIS Block Design (assemble blocks according to increasingly complex patterns, maximum score of 47), CERAD Word List, Delayed Recall (recall 10 words after 5-minute delay, maximum score of 10), and Symbol Digit (verbally report numbers that match a set of given symbols as rapidly as possible, maximum score of 100; Forms A + B). The tests, the method of application and key references have been described in detail elsewhere[43]. The listwise N is 570. The *r*s among the 4 tests ranged from 0.33 to 0.65 (mean of absolute values = 0.44).

Principal component analysis was applied to these 5 tests. The FUPC accounted for 55.6% of the total test variance. Loadings on the FUPC were as follows: WAIS Information = 0.71, Verbal Fluency = 0.72, WAIS Block Design = 0.77, CERAD Word List = 0.66, Symbol Digit = 0.85.

HCS. Scores on the following cognitive ability tests were used to create the general cognitive ability factor: verbal episodic memory (NAVLT: a 12-word list learning task); Fluency (Letter fluency test: 1 min to write the words); visuospatial functioning (clock drawing task: add numbers to a predrawn clock and set the hands at 10 past 11, scored according to the system of Manos and Wu); Language (item naming: ten pictured items are depicted in the response booklet. Participants are required to write the name of each object beside it); attention/executive function (HAT A: presented with an array of letters written in lower case and have 30 s to write the capital form of each letter beside as many letters in the array as they can). The tests, the method of application and key references have been described in detail elsewhere[44]. The listwise N was 1,637. The rs among the 5 tests ranged from 0.13 to 0.44 (mean of absolute values = 0.26). Principal component analysis was applied to these 5 tests. The FUPC accounted for 41.3% of the total test variance. Loadings on the FUPC were as follows: NAVLT = 0.51, Fluency = 0.53, attention/executive function = 0.39, visuospatial functioning = 0.36, Language = 0.43

HRS. Scores on the following five cognitive ability tests were used to create the fluid-type general cognitive ability factor: Animal Fluency (number of animals named in 1 minute), Number series (total score), Delayed Recall (number of 10 nouns recalled after a 5-minute delay), Serial 7's test (subtract 7 from 100 and continue subtracting 7 from each subsequent number for a total of 5 trials), Backwards counting starting from 86. The cognitive tests, the method of application and key references have been described in detail elsewhere[45,46]. The listwise N was 7,396. Absolute *r*s among the 5 tests ranged from 0.089 to

0.340 (mean 0.211). Principal components analysis was applied to these 5 tests. The FUPC accounted for 37.8% of the total test variance. Loadings on the FUPC were as follows: Animal fluency = 0.664, Number series = 0.735, Delayed Recall = 0.624, Serial 7's = 0.632, Backward counting = 0.346.

LBC1921. Scores on the following cognitive ability tests were used to create the fluid-type general cognitive function component: Moray House Test (total score), Verbal Fluency (sum of letters C, F and L), Raven's Standard Progressive Matrices (sum of total correct answers in 20 minutes), Logical Memory (total of immediate and delayed recall). The tests, the method of application and key references have been described in detail elsewhere[47]. The listwise N was 505. The *r*s among the 4 tests ranged from 0.17 to 0.71 (mean 0.40). Principal components analysis was applied to these 4 tests. The FUPC accounted for 55.9% of the total test variance. Loadings on the FUPC were as follows: Moray House Test = 0.90, Verbal Fluency = 0.56, Raven's Standard Progressive Matrices = 0.84, Logical Memory = 0.65.

LBC1936. Scores on the following cognitive ability tests were used to create the fluid-type general cognitive function component: Moray House Test (total score), Logical Memory (total score of immediate and delayed recall), Spatial Span (total score), Four choice reaction time (mean), Verbal Fluency (sum of letters C, F and L). The tests, the method of application and key references have been described in detail elsewhere[48]. The listwise N was 983. The absolute *r*s among the 5 tests ranged from 0.16 to 0.49 (mean 0.31). Principal components analysis was applied to these 5 tests. The FUPC accounted for 45.4% of the total test variance. Loadings on the FUPC were as follows: Moray House Test = 0.83, Logical Memory = 0.65, Spatial Span = 0.63, Four choice reaction time = -0.66, Verbal Fluency = 0.57.

LIFE-Adult. Scores on the following cognitive ability tests were used to create the fluid-type general cognitive function component: 1. Trail-Making Test B (total time taken), 2. CERAD subtest animals (number of animals named), 3. CERAD learning of word list (recall of 10 items). The tests, the method of application and key references have been described in detail elsewhere[4,49-51]. The listwise N was 3,391. The *r*s among the 3 tests ranged from -0.35 to 0.44 (mean of absolute values = 0.38). Principal component analysis was applied to these 3 tests. The FUPC accounted for 57.9% of the total test variance. Loadings on the FUPC were as follows: Trail-making test B = -0.79, CERAD subtest animals = 0.71, CERAD learning of word list = 0.78.

MAP. Scores on the following cognitive ability tests were used to create the fluid-type general cognitive function component: Logical Memory (total score), Word List Memory (total score and recall), total of Digit Span Forward and Backward, Symbol Digit, Number Comparison, Line Orientation, Progressive Matrices, and Stroop word colour naming (number of colours read correctly in 30 seconds). The tests, the method of application and key references have been described in detail elsewhere[52]. The listwise N was 685. The *r*s among the 8 tests ranged from 0.14 to 0.60 (mean 0.33). Principal components analysis was applied to these 8 tests. The FUPC accounted for 41.7% of the total test variance. Loadings on the FUPC were as follows: Logical Memory = 0.62; Word List Memory and recall = 0.71; Digit Span Forward and Backward = 0.56; Symbol Digit = 0.79; Number Comparison = 0.66; Line Orientation = 0.48; Progressive Matrices = 0.58; Stroop word colour naming = 0.70.

OATS. Scores on the following cognitive ability tests were used to create the fluid-type general cognitive ability factor: Digit Symbol-coding (total correct score in 120 seconds); Semantic Fluency (number of animals named in 1 minute); Controlled Oral Word Association Test (FAS, sum of the 3 letters, F, A, S); Logical Memory Delayed Recall (WMS-III, Story A, story elements recalled after 25-35 mins delay); Benton Visual

Retention Test Recognition (BVRT 15 items, total recognition score); Block Design (WAIS-R, total score); Trail Making Test Part B (time to completion); 8. Rey Auditory Verbal Learning Test (RAVLT Total words recalled over trials 1-5 plus words recalled after 30 mins).

The tests, the method of application and key references have been described in detail elsewhere[53,54]. The listwise N was 442. The absolute *r*s among the 8 tests ranged from 0.064 and 0.525 (mean 0.287). Principal components analysis was applied to these 8 tests. The FUPC accounted for 38.21% of the total test variance. Loadings on the FUPC were as follows: Digit Symbol = 0.703; Semantic Fluency (animals) = 0.633; Controlled Oral Word Association Test = 0.531; Logical Memory delayed = 0.515; Benton Visual Recognition Test = 0.474; Block Design = 0.643; Trail Making Test B = 0.734; 8. RAVLT = 0.661

ORCADES. Scores on the following cognitive ability tests were used to create the fluid-type general cognitive function component: Digit Symbol Coding (sum of correct coding in 2 minutes); Verbal Fluency (sum of letters C, F and L); Logical Memory from Wechsler Memory Scale-III (paragraph immediate and delayed recall summed). The tests, the method of application and key references have been described in detail elsewhere[55-57]. The listwise N was 1,635. The *r*s among the 3 tests ranged from 0.30 to 0.47 (mean 0.40). Principal components analysis was applied to these 3 tests. The FUPC accounted for 60.1% of the total test variance. Loadings on the FUPC were as follows: Digit Symbol Coding = 0.83, Verbal Fluency = 0.73, Logical Memory = 0.76.

PROSPER Study – Scotland. Scores on the following cognitive ability tests were used to create the fluid-type general cognitive ability factor: The Stroop Colour Coding test (third test), the Letter-Digit-Coding test (number of letter digit matches made correctly, Picture Learning Test Immediate Recall (number correct). The tests, the method of

application and key references have been described in detail elsewhere[58]. The listwise N was 1,803. The *r*s among the three tests ranged from 0.271 to 0.519 (mean 0.372). Principal components analysis was applied to these three tests. The FUPC accounted for 58.50% of the total test variance. Loadings on the FUPC were as follows: Stroop Colour-Coding test = 0.803, Letter-Digit-Coding test = 0.831, Picture Learning Test Immediate = 0.647.

PROSPER Study – Ireland. Scores on the following cognitive ability tests were used to create the fluid-type general cognitive ability factor: The Stroop Colour-Coding test (third test), The Letter-Digit-Coding test (number of letter digit matches made correctly), Picture Learning Test Immediate recall (number correct). The tests, the method of application and key references have been described in detail elsewhere[58]. The listwise N was 1,538. The *r*s among the three tests ranged from 0.313 to 0.470 (mean 0.366). Principal components analysis was applied to these three tests. The FUPC accounted for 57.94% of the total test variance. Loadings on the FUPC were as follows: Stroop Colour-Coding test = 0.800, Letter-Digit-Coding test = 0.798, Picture Learning Test Immediate = 0.680.

PROSPER Study – the Netherlands. Scores on the following cognitive ability tests were used to create the fluid-type general cognitive ability factor: The Stroop Colour-Coding test (third test), The Letter-Digit-Coding test (number of letter digit matches made correctly), Picture Learning Test Immediate recall (number correct). The tests, the method of application and key references have been described in detail elsewhere[58]. The listwise N was 739. The *r*s among the three tests ranged from 0.305 to 0.509 (mean 0.385). Principal components analysis was applied to these three tests. The FUPC accounted for 59.26% of the total test variance. Loadings on the FUPC were as follows: Stroop Colour-Coding test = 0.803, Letter-Digit-Coding test = 0.822, Picture Learning Test Immediate = 0.676.

ROS. Scores on the following cognitive ability tests were used to create the fluid-type general cognitive function component: Logical Memory total score (sum of Logical Memory Ia immediate recall and Logical Memory IIa delayed recall), total score of Word List Memory and Recall (sum of word list memory recall, trials 1 – 3, immediate and delay after 5 minutes), total of Digit Span Forward and Backward (sum of digit span forward and digit span backward), Symbol Digit Modalities test (total number of correct matches (90 seconds)), Number Comparison (sum of number of pairs correctly classified minus number incorrectly classified (90 seconds)), Judgment of Line Orientation (total number of correct pairs (out of 15)) and Standard Progressive Matrices (total number of correctly identified missing elements (out of 17)). The tests, the method of application and key references have been described in detail elsewhere[59]. The listwise N was 705. The *r*s among the 7 tests ranged from 0.15 to 0.67 (mean 0.33). Principal components analysis was applied to these 7 tests. The FUPC accounted for 43.4% of the total test variance. Loadings on the FUPC were: Logical Memory = 0.63; Word List Memory and Recall = 0.69; Digit Span Forward and Backward = 0.55; Symbol Digit Modalities test = 0.80; Number Comparison = 0.71; Judgment of Line Orientation = 0.46; Standard Progressive Matrices = 0.71.

RSI. Scores on the following cognitive ability tests were used to create the fluid-type general cognitive function component: Stroop card 3 (time needed to complete the card), Delayed Recall (once), Letter-digit Substitution task (LDST; number correctly coded), Verbal Fluency (number of animals named within one minute), and Peg Board test (sum of pegs with left, right and both hands). The tests, the method of application and key references have been described in detail elsewhere[60]. The listwise N was 1,779. The absolute *r*s among the 5 tests ranged from 0.213 to 0.512 (mean of absolute values = 0.340). Principal component analysis was applied to these 5 tests. The FUPC accounted for 48.1% of the total test variance.

Loadings on the FUPC were as follows: Stroop = -0.737, Delayed Recall = 0.620, LDST =0.796, Verbal Fluency = 0.690, Peg Board test = 0.606.

RSII. Scores on the following cognitive ability tests were used to create the fluid-type general cognitive function component: Stroop card 3 (time needed to complete the card), delayed (once) recall, Letter-Digit Substitution task (LDST; number correctly coded), Verbal Fluency (number of animals named within one minute) and Peg Board test (sum of pegs with left, right and both hands). The tests, the method of application and key references have been described in detail elsewhere[60]. The listwise N was 1,260. The absolute *r*s among the 5 tests ranged from 0.208 to 0.526 (mean of absolute values = 0.340). Principal component analysis was applied to these 5 tests. The FUPC accounted for 48.0% of the total test variance. Loadings on the FUPC were as follows: Stroop = -0.728, Delayed Recall = 0.605, LDST = 0.809, Verbal Fluency = 0.679, Peg Board test = 0.624.

RSIII. Scores on the following cognitive ability tests were used to create the fluid-type general cognitive function component: Stroop card 3 (time needed to complete the card), Delayed Recall (once), Letter-digit Substitution task (LDST; number correctly coded), Verbal Fluency (number of animals named within one minute) and Peg Board test (sum of pegs with left, right and both hands). The tests, the method of application and key references have been described in detail elsewhere[60]. The listwise N was 2,579. The absolute *r*s among the 5 tests ranged from 0.187 to 0.471 (mean of absolute values = 0.310). Principal component analysis was applied to these 5 tests. The FUPC accounted for 45.8% of the total test variance. Loadings on the FUPC were as follows: Stroop = -0.712, Delayed Recall = 0.579, LDST = 0.806, Verbal Fluency = 0.668, Peg Board test = 0.592.

SATSA, Gender. Scores on the following cognitive ability tests were used to create the general cognitive function component: Synonyms (Forced choice; select 1 of five possible

synonyms of target word; maximum score of 30, Forms A + B), Koh's Block Design (assemble blocks according to seven different and increasingly complex patterns, maximum score of 42), Thurstone's Picture Memory Task (episodic memory/nonverbal recognition memory task of 28 pictures identified from among distracters; maximum score of 28), Symbol Digit (verbally report numbers that match a set of given symbols as rapidly as possible, maximum score of 100; Forms A + B). The tests, the method of application and key references have been described in detail elsewhere[61]. The listwise N was 734 (450 SATSA + 284 GENDER). The *r*s among the 4 tests ranged from 0.35 to 0.66 (mean of absolute values = 0.47). Principal component analysis was applied to these 4 tests. The FUPC accounted for 60.8% of the total test variance. Loadings on the FUPC were as follows: Synonyms = 0.72, Koh's Block Design = 0.84, Thurstone's Picture Memory Task = 0.70, Symbol Digit = 0.85.

Sydney MAS. Individuals from non-English speaking backgrounds were excluded. Scores on the following cognitive ability tests were used to create the fluid-type general cognitive ability factor: Digit Symbol-Coding (total correct score in 120 seconds); Semantic Fluency (number of animals named in 1 minute); Controlled Oral Word Association Test (FAS, sum of the 3 letters, F, A, S); Logical Memory Delayed Recall (WMS-III, Story A, story elements recalled after 25-35 mins delay); Benton Visual Retention Test Recognition (BVRT 15 items, total recognition score); Block Design (WAIS-R, total score); Trail Making Test Part B (time to completion); Rey Auditory Verbal Learning Test (RAVLT Total words recalled over trials 1-5 plus words recalled after 30 mins).

The tests, the method of application and key references have been described in detail elsewhere[53]. The listwise N was 727. The absolute *r*s among the 8 tests ranged from 0.152 to 0.551 (mean 0.302). Principal components analysis was applied to these 8 tests. The FUPC accounted for 39.36% of the total test variance. Loadings on the FUPC were as follows: Digit Symbol = 0.746; Semantic fluency (animals) = 0.653; Controlled Oral Word Association Test

= 0.582; Logical Memory Delayed Recall = 0.513; Benton Visual Recognition Test = 0.565; Block Design = 0.634; Trail Making Test B =0.719; RAVLT = 0.571.

Understanding Society. Scores on the following five cognitive ability tests, administered mostly during face-to-face interviews with the assistance of a computer (1.5% of interviews in the full Understanding Society sample were carried out via telephone) were used to create the fluid-type general cognitive function component: Word Recall (assessing verbal declarative memory by measuring immediate and delayed recall of a list of 10 words); Verbal Fluency (in which participants had to name as many animals as they could within one minute); Subtraction (also known as ‘Serial 7s’, where participants had to subtract 7 from 100, then subtract 7 again from their answer, and again up to a maximum of five times); Number Sequence (where participants had to complete a sequence of numbers which had one number missing); and Numerical Reasoning (in which participants had to calculate the answer to numerical problems involving real-life quantities such as monetary transactions; they were first presented with three simple items and were given two more difficult items if successful or one simpler item if not). The tests, the method of application and key references have been described in detail elsewhere[62]. The listwise N was 7,999. The *r*s among the 5 tests ranged from 0.12 to 0.46 (mean of absolute values = 0.29). Principal component analysis was applied to these 5 tests. The FUPC accounted for 44.0% of the total test variance. Loadings on the FUPC were as follows: Word Recall = 0.64, Verbal Fluency = 0.68, Subtraction = 0.49, Number Sequence = 0.70, Numerical Reasoning = 0.78.

VETSA. Scores on the following cognitive ability tests were used to create the fluid-type general cognitive function component: Armed Forces Qualification Tests (total percentile score)[63,64]; Wechsler Memory Scale 3rd Edition (WMS-3) Digit Span (total score for forward and backward conditions)[55]; WMS-3 Letter-Number Sequencing subtest (total correct); Reading Span (total score); Golden Stroop Color-Word condition (total correct

responses)[65]; Delis-Kaplan Executive Function System (DKEFS) Trail Making subtest (time to complete number-letter switching trial)[66]; Wechsler Abbreviated Scale of Intelligence (WASI) Matrix Reasoning subtest (total correct)[13]; Gottschaldt Hidden Figures test (total correct)[67]; Mental (card) Rotation (total correct)[68]; DKEFS Verbal Fluency (total of F, A, and S conditions); California Verbal Learning Test 2nd Edition (CVLT-2) Long Delay Free Recall (number of correct words)[66]; WMS-3 Logical Memory Delayed Recall (number of correct items); and WMS-3 Visual Reproduction Delayed Recall (number of correct items). The tests, the method of application and key references have been described in detail elsewhere[69,70]. The listwise N was 1337. The absolute *r*s among the 14 tests ranged from 0.10 to 0.61 (mean of absolute values = 0.29). Principal component analysis was applied to these 14 tests. The FUPC accounted for 36.2% of the total test variance. Loadings on the FUPC were as follows: AFQT = 0.741, Digit Span = 0.623, Letter-Number Sequencing = 0 .640, Spatial Span = 0.574; Reading Span = 0.618, Stroop Color-Word = 0.559, Trail Making = 0.707, Matrix Reasoning = 0.657, Hidden Figures = 0.700, Mental Rotation = 0.514, Verbal Fluency = 0.510, CVLT-2 = 0.490, Logical Memory = 0.478, Visual Reproduction = 0.528.

YFS. Scores on the following cognitive ability tests were used to create the fluid-type general cognitive function component: Paired Associates Learning test (total number of errors with an adjustment for each stage not attempted due to previous failure); Reaction Time test (Mean five-choice reaction time); Rapid Visual Information test (signal detection measure of sensitivity to the target); and Spatial Working Memory test (total errors); these were from Cambridge Neuropsychological Test Automated Battery (CANTAB). The tests, the method of application and key references have been described in detail elsewhere[71-73]. The listwise N was 1,988. The *r*s among the 4 tests ranged from 0.10 to 0.23 (mean of absolute values = 0.20). Principal component analysis was applied to these 4 tests. The FUPC accounted for 41.3 % of the total test variance. Loadings on the FUPC were as follows:

Paired Associates Learning test 0.62, Reaction Time test 0.49, Rapid Visual Information test -0.72, and Spatial Working Memory test 0.72. The FUPC was reversed prior to GWAS so that higher scores indicate better performance on the cognitive tasks.

COGENT Cohorts

ACPRC. ACPRC Manchester and Newcastle participants were cognitively evaluated twice about five years apart. Evaluations took place across two 90-minute sessions using two alternating neuropsychological test batteries between the periods of 1983 to 2003. Test Battery 1 (TB1), Session 1, covered the domains of Fluid Reasoning (Alice Heim AH4 Intelligence Tests, Parts 1 and 2) and Crystallized Knowledge (Mill Hill Vocabulary Test, Part A [synonyms] and Part B [definitions]). TB1, Session 2, covered Verbal Learning and Memory (Verbal Free Recall of 30 Words Test and Cumulative Verbal Recall of 15 Words Test) and Nonverbal Learning and Memory (Pictorial Recognition Memory Test). Test Battery 2 (TB2), Session 1, covered the domains of Fluid Reasoning (Cattell Culture Fair Test), Crystallized Knowledge (WAIS Vocabulary Test), and Processing Speed (Savage Alphabet Coding Test). TB2, Session 2, covered Processing Speed (Visual Search for Letters Test), Verbal Learning and Memory (Verbal Free Recall for 10 Words Test and Propositions about People Test), and Nonverbal Learning and Memory (Memory for Shapes and Location Test and the Memory Circle Test)[74,75]. The listwise N was 1465.

ADNI. Measures used in ADNI included American National Adult Reading Test (ANART; total errors [reverse scored]); WMS Logical Memory (encoding plus delayed recall total score); Rey Auditory Verbal Learning Test (RAVLT; encoding plus delayed recall total score); Animal Fluency (total words in 60 seconds); Trail Making Test B (TMTB; number of

seconds to complete test); Clock Drawing (total score); and Boston Naming Test (BNT; total correct)[76-78]. The listwise N was 242.

ASPIS. As described previously[76-78], measures included: Raven Progressive Matrices Test (Raven Matrices; raw score); Continuous Performance Task, Identical Pairs version (CPT-IP; d-prime score); Verbal N-Back working memory task (Verbal NBack; total accuracy)[79]; Spatial N-Back working memory task (Spatial NBack; total accuracy). Psychometric data were unavailable, as only composite (principal component) scores were provided. The listwise N was 897.

CAMH. Available measures included: Wechsler Test for Adult Reading (WTAR)[80]; RBANS Word List Memory, Story Memory, Figure Memory, Letter-Number Span, Digit Span, Trails B, Letter Cancellation, Digit Symbol Ccoding, Letter Fluency, Animal Fluency, Line Orientation, Stroop Color-Word. The listwise N was 80.

CNP. Measures included: WASI Vocabulary, WASI Matrix Reasoning, WMS Digit Span, WMS Letter-Number Span, WMS Spatial Span and WMS Visual Reproduction, CVLT, and Choice Reaction Time (RT) task. The listwise N was 628.

DCC. DCC participants took either the CANTAB battery or a traditional cognitive testing battery or both, as described below. Both batteries were administered to all participants in a private room under supervision of a trained administrator who read the instructions from a script. The traditional battery took approximately 30 min and comprised the following tests: Trail Making Test[81] assesses rapid simple sequencing (Trails A) and complex sequencing, requiring the participant to follow a sequential pattern while shifting cognitive sets (Trails B); Controlled Oral Word Association (COWA; Multilingual Aphasia Examination)[82] measures lexical fluency across three letters; Animal Fluency is a brief measure of semantic fluency, animals named in 60 seconds; Processing Speed subtests of the

Wechsler Adult Intelligence Scale-III (WAIS-III)[55] Digit-Symbol Substitution, which assesses psychomotor sequencing, and Symbol Search, which measures scanning and target identification; Digit Span subtest of the WAIS-III[55] measures attention and concentration as reflected by Digit Span Forward and Backward; Stroop Test[83] measures sensitivity to interference through trials with changing task demands (reading, color identification, and response inhibition); and Green Prose Recall[84] quantifies immediate and 30-minute delayed recall of contextually organized stories. The CANTAB battery took approximately 1 h and comprised the following tests: Paired Associates Learning (PAL), Spatial Working Memory (SWM), Verbal Recall (VRM) Intra-Extradimensional Set Shifting (IED), Rapid Visual Processing (RVP), Pattern Recognition Memory (PRM), Spatial Span (SSP) and Spatial Recognition Memory (SRM). Further details of these tests can be found at the CANTAB web site (http://www.cantab.com/science/cantab-tests-all.asp). To reduce practice time and ceiling effects, modified versions of PAL, SWM, PRM, SSP, SRM, VRM were used for some subjects, as described in detail in Need et al.[85]. The listwise N was 1,221.

DNS. As described previously[86], DNS participants were administered a broad neuropsychological battery comprised of the following domains and tests: Processing Speed (Trail Making Test A & B); Attention & Working Memory (Digit Span Forward, Digit Span Backward, Digit Span Reordering, and the Paced Auditory Serial Addition Test [PASAT]); Phonemic Fluency (Letters [FAS]); Semantic Fluency (Categories [animals]); Crystallized Knowledge (WASI Vocabulary); and Fluid Reasoning (WASI Matrix Reasoning). The listwise N was 419.

DUBLIN. Dublin participants were administered the WTAR, as well as Vocabulary, Block Design, Similarities and Matrix Reasoning from the WASI. The listwise N was 92.

GCAP. Cognitive variables selected for GCAP (a) represented key domains of performance impairment in schizophrenia, (b) documented impairment in probands and unaffected siblings of probands, and (c) showed good distributional characteristics[87]. Domains and tests available were the following: Premorbid Ability (WRAT-3 Word Reading); Crystallized Knowledge (WAIS-R Similarities); Fluid Reasoning (WAIS-R Picture Completion); Working Memory (WAIS-R Arithmetic and Digit Span Backward); Processing Speed (WAIS-R Digit Symbol Coding and Trails A & B); Episodic Memory (Logical Memory Immediate and Delayed Recall); Verbal Fluency (Phonemic [FAS] and Categorical [animals, fruits and vegetables]); Visuoperceptual Integration (Benton Judgment of Line Orientation); and Set-shifting (Wisconsin Card Sorting Task). GCAP1 also had adequate data from Letter-Number Sequencing and the N-back, and GCAP2 had adequate data on Verbal Paired Associates and Visual Reproduction. The listwise N was 962.

GENADA. The neuropsychological battery used in GENADA consisted of the Mattis Dementia Rating Scale, 1st Edition (DRS)[88-90]; the Mini-Mental Status Exam (MMSE)[91]; and the Clock Drawing Test[92]. The DRS was designed to test core areas of cognition most affected in AD including Attention, Conceptualization, Construction, Initiation & Perseveration, and Memory. The MMSE is similarly comprised of core neurocognitive domains including Attention, Construction, Language, Memory, Orientation, and Praxis. Clock Drawing is a classic test of Visuospatial Integration and Construction. The listwise N was 782.

HBCS. The cognitive functions test scores were obtained from the Finnish Defense Forces Basic Ability Test, developed by the Finnish Defense Forces Education Development Center. The test battery and its psychometric properties are described in detail elsewhere[93]. In brief, the ability test battery, which was designed to measure general ability and logical thinking, is composed of verbal, arithmetic, and visuospatial reasoning subtests. Each subtest

is timed and consists of 40 multiple-choice questions that are ordered by difficulty. Correct answers were summed to obtain a test score. The verbal and arithmetic subtests comprise four types of questions. In the Verbal Reasoning test, the subject has to choose synonyms or antonyms of a given word, select a word belonging to the same category as a given word pair, identify which word of a word list does not belong in the group, and discern similar relations between word pairs. In the Arithmetic Reasoning test, the subject has to complete a series of numbers that have been arranged to follow a certain rule, to solve verbally expressed short problems, to complete simple arithmetic operations, and to choose similar relations between pairs of numbers. The Visuospatial Reasoning subtest comprises a set of matrices containing a pattern problem with one part removed; it is analogous to Raven's Progressive Matrices. The subject is asked to decide which of the given single figures completes the matrix, and the test requires the subject to conceptualize spatial relations ranging from the very obvious to the very abstract. Detailed information on the selection of the HBCS participants and on the study design can be found elsewhere[94-96]. The listwise N was 304.

IBG. All IBG subjects were administered a test covering Crystallized Knowledge (Vocabulary) and Fluid Reasoning (Block Design) from the Wechsler Adult Intelligence Scale (WAIS-III)[55] if aged 16 or older, or from the Wechsler Intelligence Scale for Children (WISC-III)[97] if younger than 16 years of age. The two-subtest combination of Vocabulary plus Block Design has excellent reliability, correlates highly with the Full Scale IQ score over a wide age range, and is a good measure of g[98]. The listwise N was 299.

LLFS. The LLFS neuropsychological battery covered the following domains and tests: Verbal Learning & Memory (Logical Memory Immediate and Delayed Recall); Attention and Working Memory (Digit Span Forward and Digit Span Backward); and Semantic Fluency (Categories [animals and vegetables]). Additional details of initial

neuropsychological findings from LLFS have been reported recently[99,100]. The listwise N was 4,081.

LOAD. As described previously[101] the LOAD cognitive test battery included the following domains and tests: Verbal Learning & Memory (Logical Memory Immediate and Delayed Recall); Attention and Working Memory (Digit Span Forward and Digit Span Backward); and Semantic Fluency (Categories [animals and vegetables]). The listwise N was 1,029.

LOGOS. As described in detail previously[102], subjects were administered three subtests of the Cambridge Neuropsychological Test Automated Battery[103] namely, Spatial Working Memory (SWM), Stockings of Cambridge (SOC), a planning and problem solving test, and Rapid Visual Information Processing (RVIP), a sustained attention test similar to a CPT. These are nonverbal tests which were administered with the aid of a high-resolution touch-sensitive screen (Advantech) and/or a response key to all subjects in the same order. Visual working memory was assessed with the N-Back Sequential Letter Task[104]. Cognitive flexibility and problem solving were assessed using a computerized version of the Wisconsin Card Sorting Test (WCST)[105]. The Stroop Interference Test[83] was used to measure the selection of appropriate response and interference. Subjects were administered the Iowa Gambling Task (IGT)[106] to assess planning based on emotional processing and integration of incentive information for decision-making. Finally, we used the Word Lists subtest of the Wechsler Memory Scale[56] to assess verbal learning and memory. The listwise N was 734.

MCTFR. Neurocognitive data from the MCTFR study was available as a composite full-scale IQ (FSIQ) score. Measurement of FSIQ was included in the design of the intake assessment for most participants, by way of an abbreviated form of the Wechsler Intelligence Scale for Children-Revised (WISC-R) or Wechsler Adult Intelligence Scale-Revised (WAIS-

R), as age-). The short forms consisted of two Performance subtests (Block Design and Picture appropriate (that is, 16 or younger, and older than 16, respectively Arrangement) and two Verbal subtests (Information and Vocabulary), the scaled scores on which were prorated to determine FSIQ. FSIQ estimates from this short form have been shown to correlate 0.94 with FSIQ from the complete test[98]. The listwise N was 5,446.

MUNICH. MUNICH1 participants completed the full German version of the Wechsler Adult Intelligence Scale–Revised (WAIS-R) IQ battery, which is called the HAWIE-R in Germany[17]. The German WAIS-R included four Crystallized Knowledge subtests (Information, Vocabulary, Similarities and Comprehension), four Fluid Reasoning subtests (Block Design, Object Assembly, Picture Completion and Picture Arrangement), two Working Memory subtests (Digit Span and Arithmetic) and one Processing Speed subtest (Digit Symbol Coding). MUNICH2 participants completed the same 11 subtests from the HAWIE-R/WAIS-R[17,107]. The listwise N was 1,095.

NCNG. NCNG participants completed a battery of psychometric tests, assessing general cognition, memory, attention and speed of processing faculties. The recruitment procedure resulted in a cognitively normal sample, skewed towards the higher functioning intelligence range. General cognitive ability was generated using a hierarchy of principal components analysis (PCA) steps as previously described[108]. The unrotated first component for three subtests from the California Verbal Learning Test-II[109] defined a Memory factor. The first component from the four conditions of D-KEFS[110] Color Word Interference Test defined a Speed factor. These two factor scores, together with the raw score from the Matrix Reasoning subscale of the Norwegian WASI[111], and the overall mean of median reaction times from a multiple choice reaction time task, were used as input for a further PCA, of which the unrotated first component defined the general cognitive ability factor. The listwise N was 625.

PNC. PNC participants completed the Penn Computerized Neurocognitive Battery (Penn CNB)[112,113]. The Penn CNB assesses the following: Abstraction & Concept Formation (Conditional Exclusion Test); Verbal Reasoning (Verbal Reasoning Test); Nonverbal Reasoning (Matrix Reasoning Test); Attention (Continuous Performance Test); Working Memory (Letter N-back Test); Verbal Memory (Word Memory Test); Nonverbal Memory (Face Memory Test and Visual Object Learning Test); and Sensory-Motor Processing Speed (Motor Praxis Test and Finger Tapping Test). The Wide Range Assessment Test 4 (WRAT-4) was also administered and included in the current analysis. For all Penn CNB tests, total correct/accuracy was the primary dependent measure. The listwise N was 4,470.

TOP. Neurocognitive assessment in TOP was carried out by psychologists trained in standardized neuropsychological testing. For TOP1, a 3-hour test battery (including measures of estimated premorbid IQ and adequate test effort) was administered in a fixed order with two breaks with refreshments. Attention & Working Memory were tested with the Digit Span Backward, Digit Span Forward and Letter Number components of the Wechsler Adult Intelligence Scale Third Edition (WAIS-III)[55] and the simple RT and d-prime components of the Bergen N-back test[114]. Verbal Fluency was tested with the Letter Fluency, Category Fluency and Category Switching components of the Delis-Kaplan Executive Function System (D-KEFS) Word Fluency Test, and Condition 1: Color Naming (CW-1), Condition 2: Word Reading components (CW-2), Condition 3: Inhibition (CW-3) and Condition 4: Inhibition/Switching components (CW-4) of the DKEFS Color-Word Interference Test[110]. Psychomotor Speed was tested with the Digit Symbol Coding component of the WAIS-III[55] and with the left and right hand average for the Grooved Pegboard Test[115]. Learning and Memory (Verbal and Nonverbal) were tested with the California Verbal Learning Test Second Edition (CVLT-II)[109], the Logical Memory I Recall Total Score of the Wechsler Memory Scale Third Edition (WMS-III)[116], and the Long Term Memory component of the

Rey Complex Figure Test (RCFT-LTM)[117]. General intelligence was tested with the Block Design, Matrix Reasoning, Similarities and Vocabulary components of the Wechsler Abbreviated Scale of Intelligence (WASI)[111], and the National Adult Reading Test (NART)[118]. The 4-subtest estimated IQ from the WASI was strongly correlated (r=.67, $P<10^{-46}$) with the computed general cognitive function component utilized in the present study. A subset of these measures were administered to TOP2 participants including the NART, Vocabulary, Similarities, Block Design, Matrix Reasoning, Digit Span, Letter-Number Span, and Logical Memory. The listwise N was 633.

ZHH. ZHH participants were recruited to serve as healthy comparison subjects for studies of patients with schizophrenia and other psychiatric disorders, and cognitive testing was mostly performed using the MATRICS Consensus Cognitive Battery (MCCB)[119,120]. The MCCB evaluates seven domains of cognitive function including: Speed of Processing (BACS Digit Symbol Coding, Trail Making Test Part A and Semantic Fluency (Animals); Attention/Vigilance (Continuous Performance Test - Identical Pairs version (CPT-IP); Working Memory (Spatial Span and Letter-Number Span); Verbal Learning (Hopkins Verbal Learning Test - Revised [HVLT-R]); Visual Learning (Brief Visuospatial Memory Test - Revised [BVMT-R]); and Reasoning & Problem Solving (NAB Mazes). The seventh MCCB domain, Social Cognition, was excluded from the current analysis, and three additional measures were added: Trails B, Phonemic Fluency (FAS) and WRAT-3 Word Reading. The listwise N was 176.

Supplementary Note 2

Cohort Descriptions

Below, in alphabetical order, there are descriptions of each of the cohorts included in the meta-analyses. In addition, Supplementary Data 18 has the following data for each cohort: cohort name, N, % women, maximum age, minimum age, mean age, SD of age, N excluded with stroke, N excluded with dementia; and Supplementary Data 19 has the following information: genotyping platform, genotyping centre, calling method, sample call rate, minor allele frequency restriction applied, Hardy-Weinberg equilibrium value applied, method for adjusting for population stratification, genetic imputation software used, genetic reference panel used, analysis software used for phenotype creation and genetic association, and additional covariates used.

Three-City Dijon Study (3C-Dijon Study)

The Three-City (3C) Study is a prospective population-based cohort study conducted in three French cities—Bordeaux, Dijon, Montpellier—comprising 9,294 participants in total[3]. To be eligible, participants had to live in one of the cities, be registered on the electoral rolls in 1999, be 65 years or older, and not be institutionalized. The study protocol was approved by the Ethical Committee of the University Hospital of Kremlin-Bicêtre and each participant provided informed consent. Data reported in this article were obtained in Dijon (3C-Dijon study), where 4,931 individuals were recruited between March 1999 and March 2001. Genotyping was performed at the Centre National de Genotypage (www.cng.fr), Evry, France, using Illumina Human610-Quad BeadChips, on 4,263 individuals, of whom 186 were excluded for the following reasons: non-Caucasian ethnicity (N=20), first-degree relatives (N=128), call rate < 0.95, gender inconsistencies, and population stratification outliers (N=38). Of the remaining 4,077 individuals, 3864 individuals had information on the

following test types: Trail Making Test B (TMTB), Benton Visual Retention Test (BVRT), and Delayed Recall (Five Words Memory Test). After excluding 212 individuals with history of stroke or dementia, the final sample size available for analysis was 3,652.

Age and Cognitive Performance Research Cohort (ACPRC)

The ACPRC Manchester and Newcastle Longitudinal Studies of Aging is a longitudinal research program in the UK[75]. Volunteers were recruited by advertisements in local community centres, newspapers, radio, and television. These began in Manchester in 1984 to 1986, with refreshment samples recruited in 1989/90 and 1991/92. The only exclusion criteria were that volunteers were at least age 45 years and were able to attend a regional test centre independently. All the research studies described were approved by the University of Manchester Research Ethics Committee. From 1999-2002, those volunteers still actively participating in the project were invited to consent to collection of blood samples to extract DNA for genetic analyses. Participants were genotyped using the Illumina 610-Quadv1 chip (Illumina, Inc., San Diego, CA, USA). Stringent QC procedures were applied to the genotype data, 549,692 SNPs were retained and 708 individuals were available for the present analysis[74].

A second set of participants was recruited from Newcastle, UK, following the same research protocol as Manchester[75]. Recruitment began in Newcastle in 1983/1984 with advertisements in local newspapers, radio and television. The Newcastle study attracted 2,052 volunteers, 513 men aged from 49 to 86 years (mean age: 65.2 y, SD = 11.8 y), and 1,539 women aged from 46 to 92 years (mean age: 67.4 y, SD = 14.3 y). All Newcastle participants were healthy, lived independently, and were able to make their way unaided to the University of Newcastle to take two different batteries of cognitive tests. The University of Newcastle Research Ethics Committee approved all the research methods. Newcastle volunteers still

actively participating in the project were invited to consent to collection of blood samples to extract DNA for genetic analyses. Participants were genotyped using the Illumina 610-Quadv1 chip (Illumina, Inc., San Diego, CA, USA). Stringent QC procedures were applied to the genotype data, 549,692 SNPs were retained and 757 individuals were available for the present analysis[74]. Additional Manchester and Newcastle details were published previously[75].

AgeCoDe

The AgeCoDe study started in 2003 and consists of 3,327 non-demented people over 75 years old, who were randomly selected from the general-practice registry in six German cities (Bonn, Dusseldorf, Hamburg, Leipzig, Mannheim, and Munich)[121]. Subjects were followed-up in 1.5 year intervals and received cognitive testing at each follow-up[4]. The general cognitive function phenotype was determined at the third follow-up of the study where a broader cognitive assessment was available. 1,995 subjects were assessed at this follow-up who had a mean age of 83.9 years. 1,322 (60.3%) subjects were female and all subjects were of European ancestry. For 899 subjects genome-wide data were available. Among participants with genome-wide data, we excluded patients with prevalent and incident dementia (N=140) and stroke (N=84) during the following six years, and those with insufficient neuropsychological data (N=58). After application of these criteria, 617 individuals were available for the phenotype determination. Afterwards our sample was subdivided in two subsamples (hereafter called AgeCoDe_Basel and AgeCoDe_DietBB) who were genotyped on different genotyping platforms and analysed separately. In each subsample quality control measures were applied; 358 subjects remained for the AgeCoDe_Basel, and 180 subjects for the AgeCoDe_DietBB subsample. Genotyping of the AgeCoDe_Basel subsample was performed at Division of Molecular and Cognitive Neuroscience, University of Basel. Genotyping of the AgeCoDe_DietBB subsample was performed at Life and Brain Centre, Department of Genomics, University of Bonn. Principal

component analysis (PCA) solutions in the subsamples were almost identical to the solution in the whole sample (N=617) and computed factor scores correlated perfectly (r=0.999) with each other. We chose to use component scores of PCA based on the whole sample due to bigger sample size, which may enhance stability and generalizability of the PCA solution.

Aging Gene-Environment Susceptibility – Reykjavik Study (AGES)

The AGES-Reykjavik Study is a single center prospective cohort study based on the Reykjavik Study. The Reykjavik Study was initiated in 1967 by the Icelandic Heart Association to study cardiovascular disease and risk factors. The cohort included men and women born between 1907 and 1935 who lived in Reykjavik at the 1967 baseline examination. Re-examination of surviving members of the cohort was initiated in 2002 as part of the AGES-Reykjavik Study. The AGES-Reykjavik Study is designed to investigate aging using a multifaceted comprehensive approach that includes detailed measures of brain function and structure. All cohort members were European Caucasians. Briefly, as part of a comprehensive examination, all participants answered a questionnaire, underwent a clinical examination, and had blood drawn[122]. All consenting participants were requested to take a neuropsychological test battery[10]. Among participants with genome-wide data, 2,862 participants were available for the present analysis.

The Airwave Health Monitoring Study (Airwave)

The Airwave Health Monitoring Study was established to evaluate possible health risks associated with use of TETRA, a digital communication system used by police forces and other emergency services in Great Britain since 2001. The study has been broadened to investigate more generally the health of the work force. From 2004, participants from each force who agreed to participate were enrolled either with an enrolment questionnaire or a comprehensive health screening performed locally. This includes a questionnaire, 7-day food

diaries, anthropometry, measurements of cardiovascular and cognitive function, blood chemistry, coagulation, and haematology. Blood and urine samples are stored in vapour phase liquid nitrogen allowing long-term access for biochemical or genetic analysis. By the end of 2012, the study had recruited 42,112 participants, of whom 35,199 (83.6%) had attended the health screening. Almost two thirds of participants were men and 71% of them were a TETRA user. The Airwave Health Monitoring Study is the only large-scale cohort study of police employees worldwide. Participants have consented to the use of their data and samples for future research purposes.

Alzheimer's Disease Neuroimaging Initiative (ADNI)

Data used in the present analysis were obtained from the Alzheimer's Disease Neuroimaging Initiative (ADNI) database (adni.loni.usc.edu). ADNI was launched in 2003 by the National Institute on Aging (NIA), the National Institute of Biomedical Imaging and Bioengineering (NIBIB), the Food and Drug Administration (FDA), private pharmaceutical companies and non-profit organizations, as a $60 million, 5-year public-private partnership. The primary goal of ADNI has been to test whether serial magnetic resonance imaging (MRI), positron emission tomography (PET), other biological markers, and clinical and neuropsychological assessment can be combined to measure the progression of mild cognitive impairment (MCI) and early Alzheimer's disease (AD). ADNI is the result of efforts of many co-investigators from a broad range of academic institutions and private corporations, and subjects have been recruited from over 50 sites across the U.S. and Canada. The initial goal of ADNI was to recruit 800 subjects, but ADNI has been followed by ADNI-GO and ADNI-2. To date these three protocols have recruited over 1,500 adults, ages 55 to 90, to participate in the research, consisting of cognitively normal older individuals, people with early or late MCI, and people with early AD. The follow up duration of each group is specified in the protocols for ADNI-1, ADNI-2 and ADNI-GO. Subjects originally recruited

for ADNI-1 and ADNI-GO had the option to be followed in ADNI-2. For up-to-date information, see www.adni-info.org.[76-78].

The current study included clinically stable healthy controls from ADNI-1 and ADNI-2GO. All ADNI participants provided written informed consent, and the institutional review board of each ADNI site approved study protocols. The neuropsychological tests selected for ADNI were mostly measures used by Alzheimer Disease Centers as part of a collective Uniform Data Set (UDS). The UDS is one part of a national collaborative research facilitated by the National Alzheimer's Coordinating Center and among approximately 30 Alzheimer's Disease Centers funded throughout the U.S. by NIA. ADNI-1 was genotyped on the Illumina Human610-Quad BeadChip (620,901 SNP and CNV markers), and ADNI-2GO was genotyped on the Illumina HumanOmniExpress BeadChip (730,525 SNP and CNV markers).

The Atherosclerosis Risk in Communities Study (ARIC)

The ARIC study is a prospective population-based study of atherosclerosis and clinical atherosclerotic diseases in 15,792 men and women, including 11,478 white participants, drawn from four United States communities (Suburban Minneapolis, Minnesota; Washington County, Maryland; Forsyth County, North Carolina; and Jackson, Mississippi). In the first three communities, the sample reflects the demographic composition of the community. In Jackson, only black residents were enrolled. Participants were between age 45 and 64 years at their baseline examination in 1987-1989 when blood was drawn for DNA extraction and participants consented to genetic testing[123]. A total of 15,020 participants, of whom 10,898 were white, were genotyped at the Broad Institute of MIT and Harvard, Cambridge, Massachusetts, and 9,345 of the latter passed QC criteria for genotyping and were available for analysis after application of all exclusion criteria. Vascular risk factors and outcomes, including transient ischemic attack and stroke, were determined in a standard

fashion[124]. The second clinical examination of the ARIC Study cohort in 1990–1992 included the following three neuropsychological tests: the Delayed Word Recall Test, the Digit Symbol Substitution Test, and the Word Fluency Test[125]. Among white participants with genome-wide data, 8,975 participants were available for the present analysis.

The Austrian Stroke Prevention Study (ASPS)

The ASPS study is a single center prospective follow-up study on the effects of vascular risk factors on brain structure and function in the normal older population of the city of Graz, Austria. The procedure of recruitment and diagnostic work-up of study participants has been described previously[126,127]. A total of 2,007 participants were randomly selected from the official community register stratified by gender and 5-year age groups. Individuals were excluded from the study if they had a history of neuropsychiatric disease, including previous stroke, transient ischemic attacks, and dementia, or an abnormal neurologic examination determined on the basis of a structured clinical interview and a physical and neurologic examination. During 2 study periods between September 1991 and March 1994 and between January 1999 and December 2003 an extended diagnostic work-up including neuropsychological testing was done in 1076 individuals aged 45 to 85 years randomly selected from the entire cohort: 509 from the first period and 567 from the second. In 1992, blood was drawn from all study participants for DNA extraction. They were all European Caucasians. Genotyping was performed in 996 participants, and those 748 who passed genotyping quality control and completed the cognitive tests were available for the present analyses.

The Austrian Stroke Prevention Study Family Study (ASPS-Fam)

ASPS-Fam is a prospective single-center community-based study on the cerebral effects of vascular risk factors in the normal aged population of the city of Graz,

Austria[128,129]. ASPS-Fam represents an extension of the Austrian Stroke Prevention Study (ASPS), which was established in 1991[126,127]. Between 2006 and 2013, study participants of the ASPS and their first-grade relatives were invited to enter ASPS-Fam. Inclusion criteria were no history of previous stroke or dementia and a normal neurologic examination. A total of 418 individuals from 176 families were included into the study. The number of members per family ranged from 2 to 6. The entire cohort underwent a thorough diagnostic workup including clinical history, laboratory evaluation, cognitive testing, and an extended vascular risk factor assessment. They were all European Caucasians. Those 311 participants who passed genotyping quality control and completed the cognitive tests were available for these analyses.

Athens Study of Psychosis Proneness and Incidence of Schizophrenia (ASPIS)

A detailed description of the ASPIS study has been reported previously[79,130,131]. Briefly, ASPIS examined randomly selected young male conscripts aged 18 to 24 years from the Greek Air Force in their first two weeks of admission to the National Air Force Basic Training Center (Tripolis, Greece). All conscripts had received a standardized screening interview by a team of military doctors of different specialties in order to exclude serious medical conditions, including documented diagnosis of psychotic disorders and substance dependence, and individuals with such conditions were not admitted for military training. In all, 2,029 eligible individuals provided a mouthwash sample for DNA extraction and completed a battery of computerized tasks measuring different aspects of neuropsychological and oculomotor performance. No conscript was excluded owing to medical conditions. All conscripts had already received a standardized screening interview by a team of army medical doctors of different specialties, and major medical conditions had been excluded. Conscripts underwent an extensive interview of computerized neurocognitive abilities and a self-rated psychometric evaluation. After obtaining written informed consent, DNA was

extracted from mouthwash samples. The study protocol was approved by the University Mental Health Research Institute (Athens, Greece) and the Johns Hopkins University Institutional Review Boards.

The Berlin Aging Study II (BASE-II)

The total sample of the BASE-II study consists of 600 younger adults and 1,600 older adults (for a detailed sample description, see Bertram et al.[132]). The cognitive data reported here were collected in an earlier study on neuromodulation in lifespan cognition[133,134]. Of the 1,600 older adults included in BASE-II, 1,414 had participated in that earlier study. Genotyping was performed using the Affymetrix Genome-Wide Human SNP Array 6.0 at the Max Planck Institute for Molecular Genetics, Berlin. After performing a standard quality control procedure, the effective sample was reduced to 1,320 individuals (53.6% female). At the time of cognitive testing, participants were 59 to 71 years of age (mean = 65.3; SD = 2.9). Recruitment of participants was based on advertisements in local newspapers and the public commuter transport system. All participants were Caucasian and all lived independently in the greater metropolitan area of Berlin, Germany. All participants reported normal or corrected vision, were right-handed, as indexed by the Edinburgh Handedness Index[135], had completed at least 8 years of education, and scored over 27 on the Mini-Mental State Examination[91]. No participant was on medications that may have affected cognition and none reported a history of head injuries, medical (e.g., heart attack), neurological (e.g., epilepsy), or psychiatric (e.g., depression) diseases.

Brisbane Adolescent Twins Study (BATS)

The Brisbane Adolescent Twin Studies (BATS)[136] include a series of projects at QIMR Berghofer Medical Research Institute, Brisbane, which have assessed a range of traits in a large population sample (>2,000 families). Here we include the BATS cognition cohort

(1,487 female and 1,296 male participants born 1979-1997), primarily comprising twins aged 16 years and their non-twin siblings (N=2,683, aged 15.4-22.3 years, M=16.4±0.7 years), 1,237 families)[137], as well as a subset of twins and non-twin siblings in the Queensland Twin Imaging (QTIM) study (N=100, aged 18.1-29.6 years, M=22.3±2.9 years, 71 families), who were also assessed on a short battery of cognitive tests[138]. Exclusion criteria were parental or self-report of head injury, neurological or psychiatric illness, substance abuse/dependence, or current use of psychoactive medication in either twin. All participants, as well as a parent or guardian for those aged under 18 years, provided written informed consent. The studies were approved by the Human Research Ethics Committee, QIMR Berghofer. For the current analyses, 2,253 participants (1,201 females) from 999 families remained after selection for 8 cognitive traits, genotyping (79.3% genotyped using Illumina Human610-Quad as previously described[139]; 20.7% using Illumina Core+Exome SNP platform), Caucasian ancestry (as determined from genotyping), and minimum age requirement (16 years). Participants comprised 947 complete twin pairs (365 monozygotic (MZ), 582 dizygotic (DZ)), 27 triplet sets (21 DZ trios, 1 MZ trio, and 5 trios including an MZ pair), 17 unpaired co-twins, and 261 non-twin siblings. They ranged in age from 16.0 to 22.3 years (M=16.4±0.7 years).

The Betula Study (BETULA)

The examined Betula sample was part of a larger prospective cohort study on memory, health and aging[25,140]. All participants were recruited by random selection from the personal registry of the Umeå community. The Betula sub-sample used here consisted of 324 participants (221 females and 103 males) aged between 45 and 95 years (mean = 65.7; SD = 9.0). All participants were native speakers of Swedish. None of the participants had any history of severe neurological illness or events; all had normal or corrected to normal vision, and were in good general health. They were non-demented based on an extensive neuropsychological examination and clinical evaluation of data obtained at the test occasions

and reviews of medical records starting from adulthood. The genotyping of the Betula sample was performed using the Illumina Human Omni Express and Omni 1S BeadChip, at the Life and Brain Centre, University of Bonn.

The Cardiovascular Health Study (CHS)

The CHS is a population-based observational cohort study of risk factors for vascular disease in adults 65 years or older conducted across 4 field centers in the United States: Sacramento County, California; Washington County, Maryland; Forsyth County, North Carolina; and Pittsburgh, Allegheny County, Pennsylvania[141]. The original predominantly white cohort of 5,201 persons was recruited in 1989-1990 from a random sample of seniors on Medicare eligibility lists. An additional 687 African-Americans were enrolled in 1992-1993, for a total sample of 5,888. Vascular risk factors and outcomes, including transient ischemic attack, stroke, cognition, and dementia, were determined using standardized protocols[28,142,143]. DNA was extracted from blood samples drawn on all participants who consented to genetic testing at their baseline examination in 1989-90 or 1992-1993. In 2007-2008, genotyping was performed at the General Clinical Research Center's Phenotyping/Genotyping Laboratory at Cedars-Sinai on 3,980 CHS participants who were free of cardiovascular disease at baseline and who had DNA available for genotyping. Because most other cohorts were predominantly white, the African American participants were excluded from this analysis to limit the potential for false positive associations due to population stratification. Among white participants, genotyping was attempted in 3,397 participants and was successful in 3,295 persons. Beginning in 1989/90 participants completed cognitive tests at 10 annual clinic visits. In addition, as part of the CHS Cognition Study[27,143], in 1997-99, participants were invited to undergo detailed neuropsychological assessment. Among participants with genome-wide data, 1,517 participants were available for the present analysis.

Center for Addiction and Mental Health (CAMH)

CAMH participants were recruited under a study protocol approved by the Research Ethics Board of CAMH, and all participants provided informed, written consent. All participants were identified as Caucasian based on self-reported ethnicity of three out of four grandparents. They were administered the Structured Clinical Interview for DSM-IV Disorders[144], and were interviewed by a psychiatrist to ensure diagnostic accuracy. Individuals with previous head trauma with loss of consciousness, neurological disorders, current or past substance dependence, and a history of a primary psychotic disorder in first-degree relatives were excluded. Eighty CAMH healthy control subjects completed cognitive testing and the genetic protocol[145,146]. All participants were screened with the Mini Mental Status Exam for dementia[91] and a urine toxicology screen. The Hand Dominance Questionnaire was used to examine handedness. All subjects underwent a battery of cognitive test that have been described previously[145] that was administered over approximately 1.5 hours. This battery assessed a wide range of cognitive domains: executive function, working memory, immediate memory, delayed or episodic memory, attention, set-shifting, response inhibition, mental flexibility, visuospatial construction, processing speed, fine visuomotor, and motor skills.

CROATIA-Korčula

The CROATIA study is part of a larger genetic epidemiology research program in Croatian island isolates, "10,001 Dalmatians". The genetic epidemiology research program in Croatian island isolates began in 1999[147], then expanded to study human genetic variation and effects of isolation and inbreeding[148,149] and finally entered the phase of focusing on diseases and gene mapping studies[150-152]. The CROATIA- Korčula study included 2857 participants, of which 1790 are available for this analysis. Participants from the CROATIA-Korčula study

were invited to undergo a neuropsychological examination. Genotyping was performed at the Institute of Human Genetics, Helmholtz Zentrum München, Germany.

Duke Cognition Cohort (DCC)

Participants were recruited to be members of the DCC to study the genetics of normal variation in cognitive performance[85,153]. Informed consent was obtained for all subjects as approved by the Duke University School of Medicine Institutional Review Board. All participants were healthy adult volunteers. Subjects were excluded if they had a Montreal Cognitive Assessment[154] score below 26, were taking a drug or combination of drugs that was decided by a pharmacist as likely to impact their cognition or be indicative of a cognitive impairment, were diagnosed with a serious neurological disorder, had a head injury resulting in memory problems, were diagnosed as learning disabled, or had a serious psychiatric history. Individuals with a blood relative already in the study were also excluded. Each subject donated 20 ml of blood or 5 ml saliva for DNA extraction. DNA was extracted using the QIAGEN (Venlo, The Netherlands) Autopure LS. The DNA was genotyped using Illumina (San Diego, CA, USA) HumanHap 300, Human610, HumanHap550, Human1M, or HumanCore genotyping chips at Duke University. Because of differences in genotyping platform, as well as differences in neuropsychological testing (described below), DCC is comprised of four sub-studies of only European participants (DCC1, n=498; DCC2, n=314; DCC3, n=234; and DCC4, n=147).

Duke Neurogenetics Study (DNS)

Participants were recruited as part of the Duke Neurogenetics Study (DNS), a study investigating biological mechanisms of individual differences in brain function and behaviour[155,156]. Informed consent was obtained for all subjects as approved by the Duke University School of Medicine Institutional Review Board. All participants were healthy,

young adult volunteers free of the following exclusion criteria: (1) medical diagnoses of cancer, stroke, head injury with loss of consciousness, untreated migraine headaches, diabetes requiring insulin treatment, chronic kidney or liver disease, or lifetime history of psychotic symptoms; (2) use of psychotropic, glucocorticoid, or hypolipidemic medication; and (3) conditions affecting cerebral blood flow and metabolism (e.g. hypertension). The DNS seeks to establish broad variability in multiple behavioural phenotypes related to psychopathology, so participants were not excluded based on diagnosis of any past or current DSM-IV Axis I or Axis II disorder. No subjects were taking psychotropic medication at the time or at least 10 days prior to study participation. DNA was isolated from saliva derived from Oragene DNA self-collection kits (DNA Genotek) customized for 23andMe (www.23andme.com). DNA extraction and genotyping were performed through 23andMe by the National Genomics Institute (NGI), a CLIA-certified clinical laboratory and subsidiary of Laboratory Corporation of America. One of two different Illumina arrays with custom content was used to provide genome-wide SNP data: the HumanOmniExpress or HumanOmniExpress-24.18-21. 419 individuals were available for the present study.

DUBLIN (Galway and Dublin, Ireland)

Healthy participants were recruited on the basis of responses to local media advertisements as a comparison group for a larger psychosis study. Participants were included only if they were aged between 18 and 65 years and satisfied the criteria of having no history of major mental health problems, intellectual disability, acquired brain injury, or substance misuse in the preceding 6 months, based on self-report. Sample participants were also excluded from the study if they reported having a first degree relative with a history of psychosis. All patients and control assessments were conducted in accordance with the relevant ethics committees' approval. All participants were of Irish ancestry (i.e., had 4 grandparents born in Ireland) and all provided written informed consent. The sample

consisted of 150 healthy individuals (mean age = 33.49 years; 84 male)[157,158]. Genetics analysis was carried out using DNA obtained from blood samples or saliva samples that were collected using Oragene DNA self-collection kits (DNA Genotek) and genotyped on the Affymetrix 6.0 array.

English Longitudinal Study of Aging (ELSA)

The English Longitudinal Study of Ageing (ELSA) is a population-representative study of individuals living in England and aged over 50 years[30,159]. The participants had originally participated in the Health Survey for England in 1998, 1999, or 2001. Beginning in 2002 with a sample size of 11,391, the ELSA participants have been followed up every two years. At waves 3, 4, 6, and 7 the study was replenished with new study participants from HSE to maintain the size and representativeness of the panel. Cognitive testing data were available at testing waves 1-5 for 14,432 participants. For the present analysis, 6,909 participants had usable phenotypic and genetic data.

The Zoetermeer Study (EPOZ)

The Zoetermeer Study, also called EPOZ (Epidemiologisch Preventief Onderzoek Zoetermeer, translated: Epidemiological Preventive Study Zoetermeer), is a population-based prospective cohort study among 10,361 persons aged 5 to 91 years at the baseline year (1975) that was originally concerned with the prevalence of various chronic diseases[160]. All participants were included between 1975 and 1978. Zoetermeer is a suburban residential community (at that time, with about 55,000 inhabitants) that is near The Hague in the Netherlands.

For the current focus cohort, 909 subjects aged between 60 and 90 years who were randomly selected in strata of age (5-year strata), sex, and study from a larger pool of subjects in the appropriate age groups from the 2 primary studies. On agreement of participation, a list

of contraindications (dementia, contraindications for magnetic resonance imaging [MRI] scanning, blindness) was reviewed to assess eligibility. Of these 909 individuals, 819 subjects were eligible. Among the eligible participants, 514 (63%) agreed to have an MRI brain scan as well as cognitive testing and each individual received up to three scans during 1995-2009[31].

Genotyping was performed in 450 individuals at the Erasmus Medical Center. Quality control measures were applied and 422 participants remained. Among participants with genome-wide data, 378 individuals were available for the present analysis after exclusion.

Erasmus Rucphen Family Study (ERF)

The ERF study is a family-based cohort study in a genetically isolated population in the Netherlands[161,162] including 3,000 participants. Participants are all descendants of a limited number of founders living in the 19th century. Extensive genealogical data are available for this population. The study protocol included venous puncture for DNA isolation and biochemistry, cognitive evaluation, cardiovascular examination, eye assessments, and body composition measurements. Genotyping was done at the Human Genotyping Facility, Genetic Laboratory Department of Internal Medicine, Erasmus MC, Rotterdam, and at the Genotyping Center of Leiden University, The Netherlands. In total, 2,385 samples from the ERF Study were available with good quality genotyping data. Participants were invited to undergo a neuropsychological evaluation. Among participants with genome-wide data, a total of 1,473 participants were available for the present analysis.

Framingham Heart Study (FHS)

The FHS is a three-generation, single-site, community-based, prospective cohort study that was initiated in 1948 to investigate risk factors for cardiovascular disease including stroke. It now comprises 3 generations of participants: the original cohort followed since

1948 (Original)[163]; their offspring and spouses of the offspring, followed since 1971 (Offspring)[164]; and children from the largest offspring families enrolled in 2000 (Gen 3)[165]. The Original cohort enrolled 5,209 men and women who comprised two-thirds of the adult population then residing in Framingham, MA, USA. Survivors continue to receive biennial examinations. The Offspring cohort comprises 5,124 persons (including 3,514 biological offspring) who have been examined approximately once every 4 years. Participants in the first two generations were invited to undergo an initial neuropsychological test battery in 1999-2005[33]. Neuropsychological testing in Gen 3 began in 2009. The population of Framingham was almost entirely white in 1948 when the Original cohort was recruited. Vascular risk factors and outcomes, including transient ischemic attack, stroke and dementia, were identified prospectively since 1948 through an ongoing system of FHS clinic and local hospital surveillance[166,167]. Participants had DNA extracted and provided consent for genotyping in the 1990s. Genotyping was performed at Affymetrix (Santa Clara, CA) through an NHLBI funded SNP-Health Association Resource (SHARe) project and successful in 8,481 persons from the Original, the Offspring, and the Gen3 cohorts. 4,353 genotyped participants have undergone neuropsychological testing. After excluding participants with a neurological condition that might confound the cognitive assessment (e.g., brain tumour or severe head injury), 4,187 participants were available for the present analysis.

Finnish Twin Cohort

The Finnish Twin Cohort sample included twins from two population-based Finnish Twin studies: the Finntwin12 (FT12, twins born in 1983-1987) and the Finntwin16 (FT16, twins born in 1974-1979)[168,169]. A total of 1,408 individuals (638 males and 770 females) had data on cognitive testing at a mean age of 23.48 (SD=2.12, range=21–30) years. Genotyping was performed with the Human670-QuadCustom Illumina BeadChip, and the Illumina Human Core Exome BeadChip at the Welcome Trust Sanger Institute, Cambridge, UK, and

the Broad Institute of MIT and Harvard, USA. A total of 1,133 individuals were included in the genetic analyses (only one member from monozygotic twin pairs was included in the genetic analyses).

All participants gave written informed consent. Study protocols for FT12 and FT16 were approved by the ethical committee of Helsinki and Uusimaa hospital district, Finland and the Institutional Review Board of Indiana University, Bloomington, USA.

Genetic Epidemiology Network of Arteriopathy (GENOA)

The Genetic Epidemiology Network of Arteriopathy (GENOA) study consists of hypertensive sibships that were recruited for linkage and association studies in order to identify genes that influence blood pressure and its target organ damage[170]. In the initial phase of the GENOA study (Phase I: 1996-2001), all members of sibships containing ≥ 2 individuals with essential hypertension clinically diagnosed before age 60 were invited to participate, including both hypertensive and normotensive siblings. In the second phase of the GENOA study (Phase II: 2000-2004), 1,239 European American participants were successfully re-recruited to measure potential target organ damage due to hypertension. From 2001-2006, Phase II GENOA participants that had a sibling willing and eligible to participate underwent a neurocognitive testing battery to assess several domains of cognitive function including learning, memory, attention, concentration, and language (N=967). Participants were excluded from this analysis if they were less than 45 years of age, had history of a stroke, or had unavailable genotype data. A total of 775 European American GENOA participants were included in this analysis.

Generation Scotland (GS)

Generation Scotland: the Scottish Family Health Study[36,37] is a family-structured, population-based cohort study recruited between 2006 and 2011. Regional sampling occurred

in Glasgow, Tayside, Ayrshire, Arran, and North-East Scotland, yielding a total sample size of 24,084 with an age range between 18 and 100, and up to four generations per family. A full description of the cohort is provided elsewhere[36,37] and online at http://www.generationscotland.org/. In the current analysis only subjects with no history of dementia or stroke were included, leaving an analysis sample of 18,830. Genotyping was performed at the Edinburgh Clinical Research Facility, University of Edinburgh[171]. The mean age of the sample was 47.1 years (SD=14.1) and 11,150 (59.2%) of the population were women.

Genotype-Phenotype Associations in Alzheimer's Disease (GENADA)

GENADA is a multi-site study funded by GlaxoSmithKline, Inc. to study the genetics of Alzheimer's disease (AD). As previously described[76,172,173] this Canadian dataset comprised almost 1,000 patients with AD and almost 1,000 non-demented control subjects recruited from nine memory referral clinics in Canada between June 4, 2002 and March 30, 2005. All study participants voluntarily provided an informed and signed consent by self and/or legal representative. To date, data from 801 cases with probable AD and 782 controls without a family history of dementia were available for download from dbGaP; however, only Caucasian healthy controls with adequate SNP and cognitive data were included in the current analysis (N=768). Subjects were genotyped on the Affymetrix GeneChip Human Mapping 500K Array Set. The Mapping 500K platform comprises 2 arrays, the ~262,000 marker Nsp array and the ~238,000 marker Sty array. Combined, the two arrays yielded 500,568 SNPs.

HARMONY twin sample

The Study of Dementia in Swedish Twins (HARMONY) includes like- and unlike-sex twin pairs born before 1935[14,174,175]. A total of 14,435 twins were ascertained for

dementia, with a subset of 1,557 twins receiving a complete clinical evaluation given a suspicion of dementia, including cognitive, physical and medical traits. DNA was extracted from blood samples collected at the evaluation visit. Genotyping using the Illumina Infinium PsychArray ("PsychChip"; https://www.illumina.com/products/by-type/microarray-kits/infinium-psycharray.html) was performed by the SNP&SEQ Technology Platform, Science for Life Laboratory, Uppsala, Sweden [http://snpseq.medsci.uu.se/genotyping/snp-services/]. Among HARMONY participants with genome-wide data, and without a history of stroke or dementia and who were not already participants in the aforementioned SATSA and Gender studies, 448 individuals were available for the present analysis (N=222 or 49.6% female). The sample was on average 77.84 years (SD = 6.99).

Helsinki Birth Cohort Study (HBCS)

HBCS is composed of 8,760 individuals born between the years 1934-44 in one of the two main maternity hospitals in Helsinki, Finland. Between 2001 and 2003, a randomly selected sample of 928 males and 1,075 females participated in a clinical follow-up study with a focus on cardiovascular, metabolic and reproductive health, cognitive function and depressive symptoms. Of those with genotype data, 320 men participated in assessment of cognitive functions in 2009 at the mean age of 67.7 years (SD=2.3). The research plan of the HBCS was approved by the Institutional Review Board of the National Public Health Institute and all participants have signed an informed consent. HBCS samples were genotyped on the Illumina Infinium 610K Quad chip by the Wellcome Trust Sanger Institute, Cambridge, UK using standard procedures.

Hunter Community Study (HCS)

The Hunter Community Study (HCS) is a population-based prospective cohort study of community-dwelling men and women aged 55–85 years of age who reside in Newcastle,

New South Wales (NSW), Australia. The cohort comprises 3,253 participants that were randomly selected from the NSW State electoral roll between 2004 and 2007[176].

Health and Retirement Study (HRS)

The Health and Retirement Study (HRS) is a longitudinal survey of a representative sample of Americans over the age of 50[177-179]. The current sample is over 26,000 persons in 17,000 households. Respondents are interviewed every two years about income and wealth, health and use of health services, work and retirement, and family connections. DNA was extracted from saliva collected during a face-to-face interview in the respondents' homes. These data represent respondents who provided DNA samples and signed consent forms in 2006, 2008, and 2010. Respondents were removed if they had a previous stroke, had dementia, or had missing genotype or phenotype data.

Institute for Behavioral Genetics (IBG)

The IBG samples were collected as part of two population based twin registries: the Colorado Longitudinal Twin Sample (LTS) and the Colorado Twin Sample (CTS)[180]. Inclusion criteria were that individuals be twins who lived in the area; there were no exclusion criteria with respect to IQ or psychiatric illness. Written informed consent (or assent from minors with consent from parent/guardian) was obtained from all participants. IBG youths of European ancestry were included (N=299). IBG samples were genotyped on the Affymetrix 6.0 SNP chip.

Late Onset Alzheimer's Disease Family Study (LOAD)

The NIA-sponsored LOAD Family AD Study is an extensive effort to ascertain well-characterized families and patients with and without AD[101]. The goal of LOAD was to identify and recruit families with two or more siblings with the late-onset form of AD and a cohort of unrelated, non-demented controls similar in age and ethnic background, and to

make the clinical and genetic data available to qualified investigators. A set of 1,074 Caucasian control individuals of European ancestry with genetic and neuropsychological data from LOAD were included in the current analysis. As previously described[101], the recruitment criteria included a family with multiple members affected with late-onset AD that could provide clinical information and a biological sample for DNA extraction. The proband had to have a diagnosis of definite or probable late-onset AD with onset after 60 years of age, and a full sibling with definite, probable, or possible late-onset AD with onset after 60 years of age. A third biologically-related family member was required (first-, second-, or third-degree relative) of the affected sibling pairs and 60 years or older if unaffected, or 50 years or older if diagnosed as having late-onset AD or mild cognitive impairment. Unaffected persons were required to have had documented cognitive testing and clinical examination results to verify the clinical designation. LOAD participants were genotyped using the Illumina assay protocol with hybridization to Illumina Infinium II Human 610Quadv1_B Beadchips, conducted by the Center for Inherited Disease Research (CIDR)[101,181].

Learning on Genetics of Schizophrenia Spectrum (LOGOS)

The LOGOS project recruited 1,540 randomly selected young male conscripts from the Greek Army (mean age=22.13; range=18–44) between June 2008 and July 2011 at the Military Training Camp of Candidate, Supply Army officers (SEAP) in Heraklion, Crete. Following public presentation of the study’s methods and goals in each consecutive series of new conscripts, all participants willing to volunteer received a detailed information sheet and gave written informed consent before screening. All subjects were thoroughly screened for past or current physical and mental health status by the army medical authorities, the study nurse and a trained research psychologist. They underwent a Mini-International Neuropsychiatric Interview[182] and were tested on a single occasion at some point during their 2 months military training in this establishment. Inclusion criteria were recent (last two

months) conscript status in the camp and written informed consent. Exclusion criteria were left-handedness (n=150), personal history of head trauma, medical and neurological conditions (n=68), personal history of DSM-IV Axis I disorders (n=95), current use of prescribed drugs or a positive recreational drug screen (n=0) and performance on a hearing test (n=53). On the basis of these criteria, and after 47 subjects who dropped out, cognitive and genetic data were available for 866 subjects. The LOGOS study was approved by the Ethics Committee of the University of Crete, the Executive Army Bureau, and the Bureau for the Protection of Personal and Sensitive Data of the Greek State. LOGOS was genotyped on Illumina HumanOmniExpress array.

Long Life Family Study (LLFS)

LLFS is an international family-based cohort study designed to examine genetic, behavioral and environmental factors associated with exceptional survival traits[183]. LLFS enrolled 4,559 long-lived probands and their siblings (n=1,445), their offspring (n=2,329) and spouse controls (n=785)[183]. The recruitment of families into the LLFS focused on selecting families with multiple exceptionally old living individuals. Families were recruited through elderly probands (generally in their 90's) who self-reported on the survival history of their parents and siblings, and based on this information, families which showed clustering of exceptional survival were recruited. LLFS probands resided in the catchment areas of four Field Centers (Boston University, Columbia University, University of Pittsburgh, and University of Southern Denmark). Recruited family members were phenotyped through extensive in-home visits by teams of technicians who travelled all over the USA and Denmark. LLFS blood assays were centrally processed at a Laboratory Core at the University of Minnesota, and study protocols were standardized, monitored and coordinated through a Data Management Coordinating Center at Washington University St. Louis. A total of 4,953 LLFS participants were phenotyped in all major domains of healthy aging including

cognition. Of these, 4,815 gave dbGaP sharing permission and had sufficient DNA for GWAS genotyping. LLFS subjects were genotyped on the Illumina 2.5M HumanOmni array, and genotypes were called using Bead Studio. To assess Mendelian errors on autosomal chromosomes, LOKIv3 was run on family data and removed 3,647 SNPs with enough Mendelian errors to be considered outlier SNPs. For SNPs that had Mendel errors, but not enough to be considered outlier SNPs, calls for that SNP were set to missing within each family that had a Mendel error which occurred 153,363 times in the data. Autosomal marker data were removed for 18 individuals who had an autosomal SNP call rate <97.5%, which were considered outliers as compared to the rest of the population. As a final familial QC check, Graphical Representation of Relationships (GRR) was used to check familial relationships based on Identity-by-State; corrections to the family relationships were made as warranted by the data. Quality control procedures for SNPs included eliminating SNPs with a call rate less than 98% (n=83,774; however, 1,188 of these were Mendelian outlier SNPs). Applying both the call rate and Mendelian error criteria, 86,233 autosomal SNPs were removed, leaving ≈ 2 million SNPs passing these QC criteria. Additional SNP QC included the following: MAF <1%; deviation from Hardy-Weinberg equilibrium at $P < 1E\text{-}06$; if there was an allelic mismatch with 1000HG, and if the SNP was not present in 1000 HG. After these QC procedures, genotypes remained for 4,667 European ancestry participants. In the current study, we included only Caucasian subjects of European ancestry, which resulted in a sample of 4,081 individuals for whom SNP and cognitive data were available.

Lothian Birth Cohorts 1921 (LBC1921) and 1936 (LBC1936)

The Lothian Birth Cohorts include surviving participants from the Scottish Mental Surveys of 1932 or 1947 (SMS1932 and SMS1947), having been born, respectively in 1921 (LBC1921) and 1936 (LBC1936)[47,48,184]. The LBC1921 cohort consists of 550 relatively healthy individuals, 316 females and 234 males, assessed on cognitive and medical traits at

about 79 years of age. When tested, the sample had a mean age of 79.1 years (SD = 0.6). The LBC1936 consists of 1,091 relatively healthy individuals assessed on cognitive and medical traits at about 70 years of age. At baseline the sample of 548 men and 543 women had a mean age 69.5 years (SD = 0.8). They were all Caucasian and almost all lived independently in the Lothian region (Edinburgh city and surrounding area) of Scotland. Genotyping was performed at the Edinburgh Clinical Research Facility, University of Edinburgh. Quality control measures were applied; 517 and 1,005 participants remained for LBC1921 and LBC1936 respectively. Among participants with genome-wide data, 459 (LBC1921) and 934 (LBC1936) individuals were available for the present analysis.

LIFE-Adult

LIFE-Adult is a population-based study and a part of the large-scale research project LIFE (Leipzig Research Center for Civilization Diseases). 10,000 residents (main age range 40 – 79 years) from the district of Leipzig (Saxony, Germany) were recruited and extensively phenotyped for a number of diseases and environmental parameters (see Loeffler et al.[49] for a description of the assessment programme). All subjects gave written informed consent to participate in the study. The procedures were conducted according to the Declaration of Helsinki and approved by the University of Leipzig's ethics committee (registration-number: 263-2009-14122009).

The Rush Memory and Aging Project (MAP)

The MAP, started in 1997, enrolled older men and women from assisted living facilities in the Chicago area with no evidence on dementia at baseline[52]. Since October 1997, 1815 participants completed their baseline evaluation, of whom 1,701 were non-Hispanic white people. The follow-up rate of survivors exceeds 90%. Participants agreed to annual clinical evaluations, and signed both an informed consent and an Anatomic Gift Act form

donating their brains at time of death. A more detailed description of the MAP has been published previously[52]. Participants were invited to take a neuropsychological test battery. DNA was extracted from whole blood, lymphocytes, or frozen post-mortem brain tissue. Genotyping was performed at the Broad Institute's Center for Genotyping and the Translational Genomics Research Institute and the Children's Hospital of Philadelphia[185]. Among participants with genome-wide data, 685 individuals were available for the present analysis.

Minnesota Center for Twin and Family Research (MCTFR)

Data used in the current study were accessed as part of the MCTFR Genome-Wide Association Study of Behavioral Disinhibition. This is an epidemiological study of substance abuse and related psychopathology in which the subjects were drawn from the Minnesota Center for Twin and Family Research. The MCTFR is a 20-year, longitudinal, community-representative study conducted at the University of Minnesota and approved by the University of Minnesota Institutional Review Board continuously since inception. It is in part a longitudinal study of two cohorts of adolescent twins and their parents. It additionally includes a parallel longitudinal study of adolescent adoptive siblings, biologically related siblings, and their parents. Over 1,500 twin families and 350 adoptive and biological sibling families have been studied, with follow-up assessments occurring approximately every 3 years. The MCTFR gathered detailed, standardized data on study participants including DSM-IIIR and DSM-IV diagnostic interview and questionnaire data. For the Genome-Wide Association Study of Behavioral Disinhibition, parental intake data plus adolescent data gathered closest to the proband child's 17th birthday (between ages 16.5 and 21) were used[186]. The MCTFR twin-family sample was ascertained through Minnesota birth records. The adoptive-family sample was ascertained from infant placements made by the three largest private adoption agencies in Minnesota. The non-adoptive controls were ascertained

through Minnesota state birth records and selected to have a pair of siblings of comparable age and gender to the adoptive sibling pairs. Eligibility requirements for the adoptive families included having, at the time of the intake assessment, an adopted adolescent between the ages of 11 and 21 years who had been placed permanently in the adoptive home prior to the age of 2 years and a second adolescent in the home who was not biologically related to the adopted adolescent and who was no more than 5 years different in age. The second child could be biologically related to one or both of the parents or could, like the first child, have been adopted and placed prior to the age of 2 years. Additional eligibility requirements, which applied to all subjects, included living within a day's drive of the University of Minnesota laboratory and not having any physical or mental disability that would preclude completing the day-long, in-person intake assessment. To be included in this analysis, the subject must additionally have been willing to make a blood or saliva donation. 5,446 individuals were available for the present study.

MUNICH (Munich, Germany)

The first group of participants (MUNICH1) were randomly selected from the general population of Munich, Germany, and contacted by mail. To exclude subjects with central neurological diseases and psychotic disorders or subjects who had first-degree relatives with psychotic disorders, several screenings were conducted before the volunteers were enrolled in the study. First, subjects who responded were initially screened by telephone for the absence of neuropsychiatric disorders. Second, detailed medical and psychiatric histories were assessed for both themselves and their first-degree relatives by using a semi-structured interview. Third, if no exclusion criteria were fulfilled, subjects were invited to a comprehensive interview including the Structured Clinical Interview for DSM-IV Axis I Disorders–Patient Edition[187] and the Structured Clinical Interview for DSM-IV Axis II Personality Disorders[188] to validate the absence of any lifetime psychotic disorder.

Additionally, the Family History Assessment Module[189] was conducted to exclude psychotic disorders among first-degree relatives. A neurological examination was also conducted to exclude subjects with current central nervous system impairment. In volunteers older than 60 years, the Mini-Mental Status Test[190] was performed to exclude subjects with possible cognitive impairment. The first Munich sample was genotyped on the Illumina OmniExpress chip.

A second set of Munich participants (MUNICH2) were also available for analysis. MUNICH2 consisted of 538 adults aged 19-72 years who were demographically similar to the first sample. The second sample was recruited by the Psychiatric Clinic and Polyclinic of Ludwig Maximilians University, and included subjects of Caucasian descent negative for severe somatic and psychiatric disorders as well as suicidal behavior, history of head injury, or neurological diseases. Absence of somatic disorders was evaluated by a semi-structured interview, absence of mental disorders and suicidal behavior with the Structured Clinical Interview for DSM-IV[191]. Additional details of the Munich samples are published[107]. The second Munich cohort was genotyped on either the Illumina HumanHap300 array or the HumanHap550-Quad array[192]. All Munich participants provided written informed consent, and DNA was extracted from whole blood samples. 1,095 individuals were available for the present study.

Norwegian Cognitive NeuroGenetics Cohort (NCNG)

NCNG study participants were recruited through newspaper advertisements in the Oslo and Bergen urban areas of Norway. All participants were interviewed and probed for past or present neurological or psychiatric diseases known to affect the central nervous system, and for history of substance abuse. Any person with a history of treatment for any of these conditions was excluded from the sample. Participants should have completed basic

education with no history of learning deficits; persons who, after initial inclusion, on subsequent testing scored more than one standard deviation below their age norm on intelligence or memory were excluded. Furthermore, persons with a score on a depression inventory indicating a previously undiagnosed depressive illness were excluded. The participants were native speakers of Norwegian. The project plan was approved by the regional ethical committee for medical research. Permission to obtain and store blood samples for genotyping in a biobank and to establish a registry with relevant information was granted by the Norwegian Department of Health. All participants gave their informed consent for participation, which included donation of a blood sample, DNA extraction and genotyping, and storage of the remaining blood sample in a biobank. NCNG genotyping has previously been described in detail[74]. NCNG DNA samples were newly extracted from blood using the Qiagen Gentra Autopure LS system (Qiagen, Valencia, CA, USA). They were genotyped on the Illumina Human610-Quad Beadchip, and 554,225 SNPs were retained following QC[74]. 625 individuals were available for the present study.

NIMH Genes, Cognition and Psychosis Program (GCAP)

GCAP participants were 639 healthy volunteers (GCAP1) and 325 unaffected siblings (GCAP2) from the NIMH Clinical Brain Disorder Branch (CBDB) Sibling Study of Schizophrenia Genetics[86]. GCAP participants were between the ages of 18 and 61 years and were Caucasians of self-identified European descent (genotype data were used either to confirm or refute self-reported European ancestry). Ruling-out of a psychiatric diagnosis was made independently by two psychiatrists/psychologists using the Structured Clinical Interview for DSM-IV Axis I disorders (SCID-I) and Structured Clinical Interview for DSM-IV Axis II disorders (SCID-II)[193]. Healthy volunteers in the GCAP study were excluded if they had first-degree relatives with schizophrenia spectrum disorders, if they were currently diagnosed with an Axis I disorder, or if they were taking neuroleptic medication. Unaffected

siblings were excluded if they had a history of a psychotic spectrum disorder or schizotypal/schizoid personality disorder. Participants were also excluded if there was a history of serious head trauma, alcohol or drug abuse within the previous 6 months, IQ less than 70, or evidence of learning disability. All participants provided written informed consent. GCAP participants were genotyped on Illumina HumanHap550K, HumanHap610-Quad, or HumanOmni2.5S microarrays at the NIMH Clinical Brain Disorders Branch[86]. After quality control procedures, 278,675 SNPs were available for subsequent genome-wide imputation for the GCAP cohort.

The Older Australian Twins Study (OATS)

Participants were recruited from the Australian Twin Registry and also through a recruitment drive. At baseline, participants were aged 65 years and over. Inclusion criteria included an ability to consent, a co-twin who also consented to participate, completion of some education in English and residence in one of the three Eastern states (Victoria, New South Wales, Queensland). Exclusion criteria included inadequate English to complete the assessment, current diagnosis of malignancy or other life-threatening medical illness and/or a current acute psychosis diagnosis. Informed consent was obtained from all participants and the ethics committees of the Australian Twin Registry, University of New South Wales, University of Melbourne, Queensland Institute of Medical Research and the South Eastern Sydney and Illawarra Area Health Service. At baseline, there were 623 participants with a mean age of 70.77 years and 65.2% of the sample were women. For further details see Sachdev et al.[54,194].

Orkney Complex Disease Study (ORCADES)

The Orkney Complex Disease Study (ORCADES) is a family-based, cross-sectional study that seeks to identify genetic factors influencing cardiovascular and other disease risk

in the population isolate of the Orkney Isles in northern Scotland[195]. Genetic diversity in this population is decreased compared to Mainland Scotland, consistent with the high levels of endogamy historically. Participants were recruited between 2005 and 2011, each having at least two grandparents from Orkney. Fasting blood samples were collected and over 300 health-related phenotypes and environmental exposures were measured in each individual, including a neuropsychological test battery. All participants gave informed consent and the study was approved by Research Ethics Committees in Orkney and Aberdeen.

Philadelphia Neurodevelopmental Cohort (PNC)

Participants were from the Philadelphia Neurodevelopmental Cohort (PNC) of the University of Pennsylvania Neurodevelopmental Genomics Study and the Children's Hospital of Philadelphia (CHOP). The NIH/NIMH funded PNC through the American Reinvestment and Recovery Act of 2009 (ARRA), and it is a collaborative research project between the Brain Behavior Laboratory at the University of Pennsylvania and the Center for Applied Genomics at the Children's Hospital of Philadelphia. PNC consists of youths aged 8-21 years who volunteered to participate in genomic studies of complex pediatric disorders[112,113,196]. As per published PNC reports, participants were first mailed a letter that described the study, followed by a scripted telephone call to establish that the individual was still interested in participation and was able to meet the minimal inclusion criteria. Inclusion criteria included (a) able to provide signed informed consent (for participants under age 18 assent and parental consent were required); (b) English language proficiency; and (c) physically and cognitively able to participate in an interview and computerized neurocognitive testing. The overall sample consisted of children who came for pediatric care, gave blood for genomic studies, and consented to be contacted for future studies. Cognitive and psychiatric assessments were conducted at home (68.8% of participants) or in the laboratory (31.2%) depending on family preference. All participants underwent clinical

assessment, including a neuropsychiatric structured interview and review of electronic medical records[112,113,196]. PNC participants completed the Penn Computerized Neurocognitive Battery (Penn CNB) to assess cognition[113,196]. Valid Penn CNB data from 8,526 PNC participants was downloaded via dbGaP. Samples were genotyped at the Center for Applied Genomics (CAG) at Children's Hospital of Philadelphia. In total, six SNP arrays were used to genotype the full cohort of 8,741 youths. The PNC subjects of European ancestry were genotyped on four different Illumina SNP chips and, using genome-wide genotype data, we determined that 4,711 of these subjects were of European ancestry with valid CNB data. Support for the collection of the data sets was provided by grant RC2MH089983 awarded to Raquel Gur and RC2MH089924 awarded to Hakon Hakonarson. All subjects were recruited through the Center for Applied Genomics at The Children's Hospital in Philadelphia.

PROspective Study of Pravastatin in the Elderly at Risk (PROSPER)

PROSPER was a prospective multicenter randomized placebo-controlled trial to assess whether treatment with pravastatin diminishes the risk of major vascular events in elderly. Between December 1997 and May 1999, subjects were screened and enrolled in Scotland (Glasgow), Ireland (Cork), and the Netherlands (Leiden). Men and women aged 70-82 years were recruited if they had pre-existing vascular disease or increased risk of such disease because of smoking, hypertension, or diabetes. A total number of 5,804 subjects were randomly assigned to pravastatin or placebo. A large number of prospective tests were performed including Biobank tests and cognitive function measurements. A detailed description of the study has been published elsewhere[197-199]. Blood pressure was measured in all subjects during the trial every 3 months by a standardized technique using Omron M4 sphygmomanometers (Omron Healthcare Inc, Bannockburn, Illinois). A whole genome wide screening has been performed in the sequential PHASE project with the use of the Illumina 660K beadchip. DNA was available for genotyping for 5,763 subjects. After QC, 5,244

subjects were left for analysis. Genotyping was performed with the Illumina 660K beadchip. After QC (call rate <95%) 557,192 SNPs were left for analysis. These were imputed to 2.5 million SNPs based on the HAPMAP built 36 with MACH imputation software.

The Religious Orders Study (ROS)

The ROS, started in 1994, enrolled Catholic priests, nuns, and brothers, from about 40 groups in 12 states[59]. Since January 1994, 1321 participants completed their baseline evaluation, of whom 1259 were non-Hispanic white. The follow-up rate of survivors exceeds 90%. Participants were free of known dementia at enrolment, agreed to annual clinical evaluations, and signed both an informed consent and an Anatomic Gift Act form donating their brains at time of death[59]. A more detailed description of the ROS has been published previously[59]. Participants were invited to take a neuropsychological test battery. DNA was extracted from whole blood, lymphocytes, or frozen post-mortem brain tissue. Genotyping was performed at the Broad Institute's Center for Genotyping and the Translational Genomics Research Institute and the Children's Hospital of Philadelphia[185]. Among participants with genome-wide data, 705 individuals were available for the present analysis.

Rotterdam Study (RSI, RSII, RSIII)

The Rotterdam Study is a population-based cohort study among inhabitants of a district of Rotterdam (Ommoord), The Netherlands, and aims to examine the determinants of disease and health in the elderly with a focus on neurogeriatric, cardiovascular, bone, and eye disease. In 1990-1993, 7,983 persons participated and were re-examined every 3 to 4 years (Rotterdam Study-I). In 1999, 3,011 individuals who had become 55 years of age or moved into the study district since the start of the study were added to the cohort (Rotterdam Study-II), and in 2006 a further extension of the cohort was initiated in which 3,932 subjects aged 45–54 years and living in the same district were included (Rotterdam Study-III)[200]. All

participants had DNA extracted at their first visit. Genotyping was attempted in participants with high-quality extracted DNA. Genotyping was done at the Human Genotyping Facility, Genetic Laboratory Department of Internal Medicine, Erasmus MC, Rotterdam, The Netherlands. Participants underwent several neuropsychological tests at the baseline and follow-up examinations[201]. Participants are continuously monitored for major events, including dementia and stroke, by automated linkage of the general practitioners' records and hospital discharge files with the study database[202,203]. Among participants with genome-wide data, 5,091 participants from the Rotterdam Study were available for the present analysis.

SATSA and Gender twin samples

The Swedish Adoption/Twin Study of Aging (SATSA) and the "Sex differences in health and aging" (GENDER) study includes twins born respectively between 1900-1948 (SATSA)[204] and 1906-1925 (GENDER)[205]. The SATSA sample of like-sex twins consists of a subsample of 859 twins aged 50 years and older assessed in-person (IPT) on cognitive, physical and medical traits since 1987. Extraction of DNA from blood samples at the third or subsequent IPT sessions was made[206]. The GENDER sample of unlike sex twins consists of a subset of 498 twins about 69 years and older assessed in-person for cognitive and medical traits beginning in 1995. Extraction of DNA from blood samples taken at first IPT was made[206]. Genotyping using the Illumina Infinium PsychArray ("PsychChip"; https://www.illumina.com/products/by-type/microarray-kits/infinium-psycharray.html) was performed by the SNP&SEQ Technology Platform, Science for Life Laboratory, Uppsala, Sweden [http://snpseq.medsci.uu.se/genotyping/snp-services/]. Among SATSA and Gender participants with genome-wide data, and without a history of stroke or dementia, 703 individuals were available for the present analysis (N=383 or 54.5% female). The combined samples were on average 70.84 years (SD = 7.97).

Sydney Memory and Ageing Study (Sydney MAS)

The Sydney Memory and Ageing Study is a longitudinal community-based study. The participants were randomly recruited from the compulsory electoral roll in Sydney and were aged 70-90 years. Exclusion criteria included limited English or a medical/psychological condition that would prevent them from completing assessments, dementia diagnosis, an age and education-adjusted MMSE score <24, psychotic symptoms, or a diagnosis of schizophrenia/bipolar disorder, multiple sclerosis, motor neuron disease, developmental disability and/or a progressive malignancy. Informed consent was provided by all participants and the ethics committees of the University of New South Wales and the South Eastern Sydney and Illawarra Area Health Service approved the study. At baseline, there were 1,037 participants with a mean age of 78.84 years and 44.8% were men. Further details are given in Sachdev et al.[53].

Thematic Organized Psychosis Research Study (TOP)

Participants were recruited in two waves as part of a large ongoing study on schizophrenia and bipolar disorder, the Thematic Organized Psychosis Research (TOP) study, which is run from the University Hospitals in Oslo, Norway. Two sub-studies (TOP1 and TOP2) were included in the present analysis, and all subjects self-reported Norwegian ancestry, and PCA of an allele-sharing distance matrix across all subjects did not suggest any non-European ancestry genetic outliers[207]. The healthy participants were randomly selected from national statistical records from the same catchment area and contacted by letter inviting them to participate. The healthy sample was screened with interview and with the Primary Care Evaluation of Mental Disorders (PRIME-MD)[208], and subjects were excluded if they or any close relatives had a history of a severe psychiatric disorder (schizophrenia, bipolar disorder and major depression), or substance abuse or dependency in the last three months. Exclusion criteria for all groups were: IQ score below 70, hospitalized head injury,

neurological disorder, unstable or uncontrolled medical condition that interferes with brain function (including hypothyroidism, uncontrolled hypertension and diabetes), and/or outside the age range 17-65 years. To assure valid neurocognitive test performance, all participants had to have Norwegian as their first language or have received their compulsory schooling in Norway, and had to score ≥15 on the forced recognition trial in the California Verbal Learning Test (CVLT-II)[109]. All participants gave written informed consent, and the study was approved by the Regional Committee for Medical Research Ethics and the Norwegian Data Inspectorate, and the Biobank was approved by the Health Department. In TOP1, DNA was collected and participants were genotyped on the Affymetrix 6.0 array and 597,198 SNPs passed quality control filters as previously reported[209,210]. In TOP2, DNA was collected and participants were genotyped on the Illumina OmniExpress array and 605K SNPs passed quality control filters. 633 individuals were available for the present study.

UCLA Consortium for Neuropsychiatric Phenomics (UCLA)

The UCLA Consortium for Neuropsychiatric Phenomics (CNP) is a large study funded by the NIH Roadmap Initiative that aims to facilitate discovery of the genetic and environmental bases of variation in psychological and neural system phenotypes, to elucidate the mechanisms that link the human genome to complex psychological syndromes, and to foster breakthroughs in the development of novel treatments for neuropsychiatric disorders. CNP comprises 8 components led by a team of 52 investigators representing diverse disciplines at the University of California, Los Angeles (UCLA), with five interlocking research projects supported by two research infrastructure cores and a coordinating center. Three research projects focus on clinical and laboratory approaches to understanding brain mechanisms underlying memory, response inhibition, and other behavioral functions disrupted in Schizophrenia, Bipolar Disorder, and Attention-Deficit/Hyperactivity Disorder. These projects examine variations in genetics, brain structure, brain function, and behavior in

2,000 healthy people and 300 suffering from one of the target neuropsychiatric syndromes, and conduct parallel basic science experiments to unravel the biological mechanisms underlying these phenotypes. The participants, ages 21-50 years, were recruited by community advertisements from the Los Angeles area and completed extensive neuropsychological testing, in addition to fMRI scanning. All participants gave written informed consent according to the procedures approved by the Institutional Review Boards at UCLA and the Los Angeles County Department of Mental Health. To be included individuals had to be either "White, Not of Hispanic or Latino Origin" or "Hispanic or Latino, of Any Race" following NIH designations of racial and ethnic minority groups. However, for purposes of the present study, only subjects clustering with European ancestry individuals based on genetic data were included. For participants who spoke both English and Spanish, language for testing was determined by a verbal fluency test. Participants were screened for neurological disease, history of head injury with loss of consciousness or cognitive sequelae, use of psychoactive medications, substance dependence within past 6 months, history of major mental illness or ADHD, and current mood or anxiety disorder. Self-reported history of psychopathology was verified with the SCID-IV. Urinalysis was used to screen for drugs of abuse (cannabis, amphetamine, opioids, cocaine, benzodiazepines) on the day of testing and excluded if results were positive. CNP subjects were genotyped on the Illumina OmniExpress-12v1_A chip.

UK Biobank

UK Biobank is a prospective cohort study designed to investigate the causes and consequences of a range of illnesses affecting middle-aged and older adults[211] (http://www.ukbiobank.ac.uk/). Between 2006 and 2010, 502,620 community-dwelling participants aged between 37 and 73 years completed the baseline assessment. Data collected at baseline included cognitive and physical measures, information on health and lifestyle, and

blood, saliva and urine samples. Sub-samples of UK Biobank participants have undergone repeat assessments, including a web-based cognitive assessment. More detailed information on data collected is reported on UK Biobank's Data Showcase (http://biobank.ctsu.ox.ac.uk/crystal/).

For this analysis, four samples of UK Biobank participants with genome-wide genotyping data who also completed the verbal-numerical reasoning test (referred to as the 'fluid intelligence test' by UK Biobank) were used. The first sample consisted of participants who completed the verbal-numerical reasoning test at the baseline assessment and also had genome-wide genotyping data following quality control procedures (n = 107,586). The second sample consists of participants who did not complete the verbal-numerical reasoning test at baseline but did complete this test at the first repeat assessment visit in assessment centres (n = 11,123). The third sample consists of participants who did not complete the verbal-numerical reasoning test at a previous testing occasion but did complete the test at the imaging visit in assessment centres (n = 3002). The fourth sample consists of participants who did not complete the verbal-numerical reasoning test at any assessment centre visit, but completed this test during the web-based assessment which took place between 2014 and 2015 and who also had genome-wide genotyping data after quality control (n = 46,322). Because there were differences in the testing conditions between the assessment centre and web-based versions of the verbal-numerical reasoning test, these samples were analysed separately in this analysis.

Full details of the UK Biobank genotyping procedure can be found elsewhere[212]. In short, two custom genotyping arrays were used to genotype 49,950 participants (UK BiLEVE Axiom Array) and 438,427 participants (UK Biobank Axiom Array)[212,213]. Genotype data (805,426 markers) were available for 488,377 individuals, with imputation to the HRC reference panel. Downstream quality control steps for the present study involved excluding

(1) those with non-British ancestry based on self-report and a principal components analysis, (2) extreme scores based on heterozygosity and missingness, (3) individuals with neither XX nor XY sex chromosomes, (4) individuals whose reported sex was inconsistent with genetically inferred sex, and (5) individuals with >10 putative third degree relatives from the kinship table. This left 408,095 individuals. All related individuals were identified using the UK Biobank QC variable for relatedness ("in.kinship.table" as described in Bycroft et al.[212]). To maximise our sample size of unrelated individuals, we created a GRM of those related individuals (N = 131,790) and then removed one of a pair of individuals based on a genetic relationship threshold of 0.025 ascertained using GCTA-GREML[214]. After implementing these steps, the sample size was 332,050, with verbal numerical reasoning data available for 168,033 individuals.

Ethical approval for UK Biobank was received from the Research Ethics Committee (REC reference 11/NW/0382).

Understanding Society: the UK Household Longitudinal Study

Understanding Society is a longitudinal study of over 40,000 households in the UK which began in 2009[215,216]. It is an amalgam of four studies, the General Population Sample, the Ethnic Minority Boost Sample, the British Household Panel Survey, and the Innovation Panel. The original household members are contacted each year, and any new members of their household are then included in the study. During the third wave of data collection (2011-2013), cognitive data were collected on five tests. A genome wide scan (Illumina human core exome array) has been conducted on DNA samples from approximately 10,000 people. We were provided with a variable ("GRMindp"), which allows identification of related individuals within the study. This was used to remove those who are more than 5%

related (n = 707). For the present analysis, 7,999 participants with phenotypic and genetic data were available.

Vietnam Era Twin Study of Aging (VETSA)

VETSA is a longitudinal behaviour genetics study of cognitive and brain aging[217,218]. There are three key features to the VETSA design. First, the sample has a narrow age range (~10 years), allowing for examination of individual differences in aging trajectories. Second, the initial assessment was in midlife (mean age=56; range=51-60), which provides a baseline for the transition to older age. Third, data previously collected on VETSA participants is also available; of particular importance is a test of general cognitive ability administered at average age 20 and repeated in each wave of the study.

Participants are members of the Vietnam Era Twin Registry, which is housed at the VA Puget Sound Health Care System in Seattle, WA, USA. All of the twins served in some branch of US military service at some time during the Vietnam era (1965-1975). A 1992 study sought to recruit all Registry twins. It enrolled approximately 8,000 individuals, including approximately 3,300 twin pairs. VETSA participants were randomly recruited from those 3,300 pairs. Eligibility for inclusion was based only on being 51-59 years old at the time of recruitment and willingness of both twins in a pair to participate. Both members of a pair did not need to participate to be included in wave 2, which was an average of 5.6 years later. Additional participants, including attrition replacement participants, were included at wave 2. Subsets have multi-modal MRI and neuroendocrine data. Data collection includes questionnaires filled out at home plus a daylong series of assessments. These include cognitive/neuropsychological assessment of multiple cognitive domains, personality and psychosocial assessments, and health/medical assessments.

There are approximately 55% MZ and 45% DZ twins in the sample. There are 1,291 individuals with wave 1 cognitive, psychosocial, and health/medical data, and 1,205 with the same wave 2 data. Of these, 1,016 have data at both time points. Structural MRIs were obtained from 545 individuals at wave 1 and 447 at wave 2. At wave 1 only, salivary cortisol, testosterone, and DHEAS data were collected on 780 participants.

VETSA participants live throughout the US. The sample is primarily Caucasian (European-American): 86% based on self-report. The average educational attainment is 13.8 years (SD = 2.1). At wave 1, 79% were married, and 78% were employed full-time. Nearly 80% report no combat experience. The sample is similar with respect to health and lifestyle characteristics to American men in their age range based on US Center for Disease Control and Prevention data.

The Cardiovascular Risk in Young Finns Study (YFS)

The Cardiovascular Risk in Young Finns Study (YFS) is an ongoing multicenter follow up study of 3,596 children and adolescents aged 3-18 years who participated in first cross-sectional study in 1980. The subjects were randomly selected from national register from the cities of Helsinki, Kuopio, Tampere, Turku, and Oulu and their rural surroundings. After the baseline study, follow-up studies have been conducted in 1983, 1986, 1989, 1992, 2001, 2007 and 2011. At the latest follow-up visit, a computerized cognitive testing battery (CANTAB®) was used to assess cognitive performance. In the YFS, a subset of tests sensitive to aging were selected from the full CANTAB battery. The sample included 945 female and 780 male subjects. The mean age of subjects who underwent cognitive testing and had genotype data available was 41.9 (SD = 5.0) years. Five subjects were excluded due to self-reported stroke prior to principal component analysis. All subjects were of Caucasian origin. The genotyping was performed with Illumina 670k Bead chip at Welcome Trust

Sanger Institute, Cambridge, United Kingdom. Quality control measures were applied prior to imputation.

Zucker Hillside Hospital (ZHH)

Participants from the New York metropolitan area were recruited through advertisements, word of mouth, referrals, and study registries. Participants had no history of a current DSM-IV Axis I major mood or psychotic disorder as assessed by structured diagnostic interview[144]. Other exclusion criteria included: (1) intellectual or learning disability; and (2) significant medical illness that could affect brain structure and/or function. Written informed consent was obtained from all participants prior to neurocognitive testing. This study was approved by the Institutional Review Board of the North Shore – Long Island Jewish Health System. ZHH participants provided blood samples for DNA extraction. DNA samples were genotyped on approximately 1M SNPs using the Illumina Omni-1Quad platform. All quality-control procedures were performed in SVS version 7.3.1 (GoldenHelix Inc), except for cryptic identity and cryptic relatedness, which were performed in Plink. Following QC, 803,582 high-quality autosomal SNPs were available for analysis in 176 individuals[219].

Supplementary Note 3

Cohort Acknowledgments

3C-Dijon

This work was made possible by the generous participation of the participants and their families. This work was supported by the National Foundation for Alzheimer's disease and related disorders, the Institut Pasteur de Lille, the Centre National de Génotypage, Inserm, FRC (fondation pour la recherche sur le cerveau) and Rotary. This work has been developed and supported by the LABEX (laboratory of excellence program investment for the future) DISTALZ grant (Development of Innovative Strategies for a Transdisciplinary approach to ALZheimer's disease). The Three-City Study was performed as part of a collaboration between the Institut National de la Santé et de la Recherche Médicale (Inserm), the Victor Segalen Bordeaux II University and Sanofi-Synthélabo. The Fondation pour la Recherche Médicale funded the preparation and initiation of the study. The 3C Study was also funded by the Caisse Nationale Maladie des Travailleurs Salariés, Direction Générale de la Santé, MGEN, Institut de la Longévité, Agence Française de Sécurité Sanitaire des Produits de Santé, the Aquitaine and Bourgogne Regional Councils, Fondation de France and the joint French Ministry of Research/INSERM "Cohortes et collections de données biologiques" programme. Lille Génopôle received an unconditional grant from Eisai. Stéphanie Debette was supported for this work by grants from the European Research Council (ERC), the EU Joint Programme - Neurodegenerative Disease Research (JPND), the Agence Nationale de la Recherche (ANR), the Initiative of Excellence of the University of Bordeaux.

AgeCoDe

Principal Investigators: Wolfgang Maier, Bonn; Martin Scherer, Hamburg; Steffi Riedel-Heller, Leipzig.

We want to thank both all participating patients and their general practitioners for their good collaboration. We also thank all additional members of the Age-CoDe Study Group. The work described in the present publication was performed within the context of the German Research Network on Dementia (KND) and the German Research Network on Degenerative Dementia (KNDD), which are funded by the German Federal Ministry of Education and Research (grants KND: 01GI0102, 01GI0420, 01GI0422, 01GI0423, 01GI0429, 01GI0431, 01GI0433, 01GI0434; grants KNDD: 01GI1007A, 01GI0710, 01GI0711, 01GI0712, 01GI0713, 01GI0714, 01GI0715, 01GI0716, 01ET1006B). Further the work is funded by the 7th framework programme of the European Union (ADAMS project, HEALTH-F4-2009-242257). Analyses was also funded by the German Federal Ministry of Education and Research (BMBF: FKZ 01EA1410A, 01EA1410B, FKZ 01EA1410E) within the project “Diet-Body-Brain (DietBB) - Competence Cluster in Nutrition Research”.

AGES

The Age, Gene/Environment Susceptibility (AGES Reykjavik) Study was initiated to examine genetic susceptibility and gene/environment interaction as these contribute to phenotypes common in old age, and represents a continuation of the Reykjavik Study cohort begun in 1967. The Age, Gene/Environment Susceptibility Reykjavik Study has been funded by NIH contract N01-AG-12100, the NIA Intramural Research Program, Hjartavernd (the Icelandic Heart Association), and the Althingi (the Icelandic Parliament). The study is approved by the Icelandic National Bioethics Committee, (VSN: 00-063) and the Data

Protection Authority. The researchers are indebted to the participants for their willingness to participate in the study.

Airwave

The Airwave study is funded by the UK Home Office (780- TETRA) with additional support from the National Institute for Health Research (NIHR), Imperial College Healthcare NHS Trust and Imperial College Biomedical Research Centre. The views expressed in this publication are those of the authors and not necessarily those of the Home Office, the Department of Health, the NHS or the NIHR. We thank all participants in the Airwave Health Monitoring Study. We also thank Louisa Cavaliero who assisted in data collection and management. The study has ethical approval through the National Health Service multi-site research ethics committee (MREC/13/NW/0588). Each participant provided informed written consent to participate in the study following procedures approved by the MREC. PE is Director of the MRC-PHE Centre for Environment and Health and acknowledges support from the Medical Research Council and Public Health England (MR/L01341X/1). PE acknowledges support from the NIHR Biomedical Research Centre at Imperial College Healthcare NHS Trust and Imperial College London, and the NIHR Health Protection Research Unit in Health Impact of Environmental Hazards (HPRU-2012-10141). PE is a UK Dementia Research Institute (DRI) Professor, UK DRI at Imperial College London. The UK DRI is funded by the Medical Research Council, Alzheimer's Society and Alzheimer's Research UK. This work used the computing resources of the UK MEDical BIOinformatics partnership (UK MED-BIO) which is supported by the Medical Research Council (MR/L01632X/1). UK Biobank genotyping was partly supported by the British Heart Foundation (grant SP/13/2/30111). PE is a member of the UK Biobank Steering Committee.

ARIC

The Atherosclerosis Risk in Communities Study is carried out as a collaborative study supported by National Heart, Lung, and Blood Institute contracts (HHSN268201100005C, HHSN268201100006C, HHSN268201100007C, HHSN268201100008C, HHSN268201100009C, HHSN268201100010C, HHSN268201100011C, and HHSN268201100012C), R01HL087641, R01HL59367, and R01HL086694; National Human Genome Research Institute contract U01HG004402; and National Institutes of Health contract HHSN268200625226C. The authors thank the staff and participants of the ARIC study for their important contributions. Infrastructure was partly supported by Grant Number UL1RR025005, a component of the National Institutes of Health and NIH Roadmap for Medical Research.

ASPS and ASPS-Fam

The authors thank the staff and the participants for their valuable contributions. We thank Birgit Reinhart for her long-term administrative commitment, Elfi Hofer for the technical assistance at creating the DNA bank, Ing. Johann Semmler and Anita Harb for DNA sequencing and DNA analyses by TaqMan assays and Irmgard Poelzl for supervising the quality management processes after ISO9001 at the biobanking and DNA analyses. The research reported in this article was funded by the Austrian Science Fund (FWF) grant number P20545-P05, P13180, PI904, P20545-B05 the Austrian National Bank Anniversary Fund, P15435, and by the Austrain Federal Ministry of Science, Research and Economy under the aegis of the EU Joint Programme-Neurodegenerative Disease Research (JPND)-www.jpnd.eu. The Medical University of Graz and the Steiermärkische Krankenanstaltengesellschaft support the databank of the ASPS.

BASE II

BASE-II has been financed by the Max Planck Society and the Federal Ministry of Education and Research.

BATS

We thank the Brisbane twins and siblings for their participation. From the QIMR Berghofer Medical Research Institute we acknowledge Marlene Grace and Ann Eldridge for sample collection; Kerrie McAloney for study co-ordination; Harry Beeby, Daniel Park, and David Smyth for IT support; and Anjali Henders and the Molecular Genetics Laboratory for DNA sample preparation. This work is supported by the Australian Research Council (A7600334, A79906588, A79801419, DP0212016, DP0664638, DP1093900) and the National Health and Medical Research Council (Medical Bioinformatics Genomics Proteomics Program, 389891). We acknowledge the University of Michigan for providing computational resources through access to the Michigan Imputation Server.

BETULA

The Betula Study was supported by grants to L.N. from Knut and Alice Wallenberg's Foundation, Sweden. SG was supported by a grant from Helse Vest RHF to SLH (Grant 911554). We also thank the Centre for Advanced Study (CAS) at the Norwegian Academy of Science and Letters in Oslo for hosting collaborative projects and workshops between Norway, Sweden and Scotland in 2011-2012.

CHS

This CHS research was supported by NHLBI contracts HHSN268201200036C, HHSN268200800007C, N01HC55222, N01HC85079, N01HC85080, N01HC85081, N01HC85082, N01HC85083, N01HC85086; and NHLBI grants U01HL080295, R01HL087652, R01HL105756, R01HL103612, R01HL120393, and R01HL130114 with

additional contribution from the National Institute of Neurological Disorders and Stroke (NINDS). Additional support was provided through R01AG023629, R01AG15928, and R01AG033193 from the National Institute on Aging (NIA). A full list of principal CHS investigators and institutions can be found at CHS-NHLBI.org. The provision of genotyping data was supported in part by the National Center for Advancing Translational Sciences, CTSI grant UL1TR000124, and the National Institute of Diabetes and Digestive and Kidney Disease Diabetes Research Center (DRC) grant DK063491 to the Southern California Diabetes Endocrinology Research Center. The content is solely the responsibility of the authors and does not necessarily represent the official views of the National Institutes of Health.

CROATIA-Korcula

We would like to acknowledge the staff of several institutions in Croatia that supported the field work, including but not limited to The University of Split and Zagreb Medical Schools and the Croatian Institute for Public Health. We would also like to acknowledge the invaluable contributions of the recruitment teams in Korcula, the administrative teams in Croatia and Edinburgh, and the people of Korcula. The CROATIA-Korcula study was funded by grants from the Medical Research Council (UK), European Commission Framework 6 project EUROSPAN (Contract No. LSHG-CT-2006-018947), European Commission Framework 7 project BBMRI-LPC (FP7 313010), the Republic of Croatia Ministry of Science, Education and Sports research grant (216-1080315-0302), the Croatian Science Foundation (grant 8875), CEKOM (Ministry of Economy, Entrepreneurship and Crafts) and the Research Centre of Excellence in Personalized Medicine (Ministry of Science and Education).

DCC

We acknowledge the Ellison Medical Foundation New Scholar award AG-NS-0441-08 (to O.C.).

DNS

We thank all members of the Laboratory of NeuroGenetics for their assistance in conducting the Duke Neurogenetics Study. M.A.S. was supported by an NSF Graduate Research Fellowship. The Duke Neurogenetics Study was supported by Duke University and NIH grant R01DA033369. A.R.H. is further supported by NIH grant R01AG049789.

ELSA

The English Longitudinal Study of Ageing is jointly run by University College London, Institute for Fiscal Studies, University of Manchester and National Centre for Social Research. Genetic analyses have been carried out by UCL Genomics and funded by the Economic and Social Research Council and the National Institute on Aging. All GWAS data has been deposited in the European Genome-phenome Archive.

Framingham Heart Study (FHS)

This work was supported by the National Heart, Lung and Blood Institute's Framingham Heart Study (Contract No. N01-HC-25195 and No. HHSN268201500001I) and its contract with Affymetrix, Inc. for genotyping services (Contract No. N02-HL-6-4278). A portion of this research utilized the Linux Cluster for Genetic Analysis (LinGA-II) funded by the Robert Dawson Evans Endowment of the Department of Medicine at Boston University School of Medicine and Boston Medical Center. This study was also supported by grants from the National Institute of Aging (R01s AG033193, AG054076, AG049607, AG008122 and AG033040; and U01-AG049505 and AG052409) and the National Institute of Neurological Disorders and Stroke (R01-NS017950). We would like to thank the dedication

of the Framingham Study participants, as well as the Framingham Study team, especially investigators and staff from the Neurology group, for their contributions to data collection. Dr. DeCarli is supported by the Alzheimer’s Disease Center (P30 AG 010129). The views expressed in this manuscript are those of the authors and do not necessarily represent the views of the National Heart, Lung, and Blood Institute; the National Institutes of Health; or the U.S. Department of Health and Human Services.

Finnish Twin Cohort

We warmly thank the participating twin pairs for their contribution. Anja Häppölä and Kauko Heikkilä are acknowledged for their valuable contribution in recruitment, data collection, and data management. Ulla Kulmala, Henri Lehtinen, Anniina Mara, Kristiina Saanakorpi, Marja Saarinen, Sanna Selinheimo, Mari Siltala, Timo Säämänen, Lauri Tapola, Annamari Tuulio-Henriksson and Sini Yli-Pohja are acknowledged for data collection. Antti-Pekka Sarin and Samuli Ripatti are acknowledged for genotype data quality controls and imputation. GWAS analyses were run at the ELIXIR Finland node hosted at CSC – IT Center for Science for ICT resources. Phenotyping and genotyping of the Finnish twin cohorts was supported by the Academy of Finland Center of Excellence in Complex Disease Genetics (grants 213506, 129680), the Academy of Finland (grants 100499, 205585, 118555, 141054, 265240, 263278 and 264146 to J. Kaprio), National Institute of Alcohol Abuse and Alcoholism (grants AA-12502, AA-00145, and AA-09203 to R. J. Rose and AA15416 and K02AA018755 to D. M. Dick), and the Welcome Trust Sanger Institute, UK.

GENDER

Gender was supported by the MacArthur Foundation Research Network on Successful Aging, The Axel and Margaret Ax:son Johnson’s Foundation, The Swedish Council for Social Research, and the Swedish Foundation for Health Care Sciences and Allergy Research

[PI: B. Malberg; A. Dahl Aslan]. DNA extraction and genotyping was supported in part by NIH R01 AG028555 (Reynolds) and NIH R01 AG037985 (Pedersen).

Generation Scotland

We would like to acknowledge the contributions of the families who took part in the Generation Scotland: Scottish Family Health Study, the general practitioners and Scottish School of Primary Care for their help in recruiting them, and the whole Generation Scotland team, which includes academic researchers, IT staff, laboratory technicians, statisticians and research managers. Generation Scotland received core funding from the Chief Scientist Office of the Scottish Government Health Directorate CZD/16/6 and the Scottish Funding Council HR03006. Genotyping of the GS:SFHS samples was carried out by the Genetics Core Laboratory at the Edinburgh Clinical Research Facility, University of Edinburgh, Scotland and was funded by the Medical Research Council UK and the Wellcome Trust (Wellcome Trust Strategic Award "STratifying Resilience and Depression Longitudinally" (STRADL) Reference 104036/Z/14/Z).

GENOA

Support for the Genetic Epidemiology Network of Arteriopathy (GENOA) was provided by the National Heart, Lung and Blood Institute (HL054464, HL054457, HL054481, HL087660, HL119443) and the National Institute of Neurological Disorders and Stroke (NS041558) of the National Institutes of Health. Genotyping was performed at the Mayo Clinic (S.T.T, Mariza de Andrade, Julie Cunningham) and was made possible by the University of Texas Health Sciences Center (Eric Boerwinkle, Megan Grove-Gaona). We would also like to thank the families that participated in the GENOA study.

HARMONY

Harmony was supported by NIH grant R01 AG08724, PI's: M. Gatz, N.L. Pedersen. DNA extraction and genotyping was supported in part by AG028555 (Reynolds) and AG037985 (Pedersen).

HCS

The authors would like to thank the men and women participating in the HCS as well as The University of Newcastle, Vincent Fairfax Family Foundation and The Hunter Medical Research Institute.

HRS

HRS is supported by the National Institute on Aging (NIA U01AG009740). Analysis of cognitive phenotypes is also supported by NIA (R03 AG048806 and R01AG055406). Genotyping was funded separately by NIA (RC2 AG036495, RC4 AG039029). Our genotyping was conducted by the NIH Center for Inherited Disease Research (CIDR) at Johns Hopkins University. Genotyping quality control and final preparation of the data were performed by the Genetics Coordinating Center at the University of Washington.

LIFE-Adult

This study is supported by LIFE – Leipzig Research Center for Civilization Diseases, Universität Leipzig. LIFE is funded by means of the European Union, by the European Regional Development Fund (ERDF) and by means of the Free State of Saxony within the framework of the excellence initiative. LIFE is funded by means of the European Union, by the European Regional Development Fund (ERDF) and by means of the Free State of Saxony within the framework of the excellence initiative.

LCB1921 and LBC1936 (LBCs)

We thank the cohort participants and team members who contributed to these studies. Phenotype collection in the Lothian Birth Cohort 1921 was supported by the UK Biotechnology and Biological Sciences Research Council (BBSRC), The Royal Society and The Chief Scientist Office of the Scottish Government. Phenotype collection in the Lothian Birth Cohort 1936 was supported by Research Into Ageing (continues as part of Age UK The Disconnected Mind project). Genotyping of the cohorts was funded by the BBSRC. The work was undertaken by The University of Edinburgh Centre for Cognitive Ageing and Cognitive Epidemiology (CCACE), part of the cross council Lifelong Health and Wellbeing Initiative (MR/K026992/1). Funding from the BBSRC and Medical Research Council (MRC) is gratefully acknowledged. Saskia Hagenaars worked alongside the LBCs-CCACE team, who also analysed the UK Biobank data; her contribution to this study represents independent research part-funded by the National Institute for Health Research (NIHR) Biomedical Research Centre at South London and Maudsley NHS Foundation Trust and King's College London.

NCNG

The NCNG study has been funded through the Research Council of Norway (including the FUGE program), the National Institutes of Health, the University of Oslo, the University of Bergen, the Bergen Research Foundation (BFS), Helse Vest, and the Western Norway Regional Health Authority, the KG Jebsen Centre for Psychosis Research, and Dr. Einar Martens Fund. We also thank the Centre for Advanced Study (CAS) at the Norwegian Academy of Science and Letters in Oslo for hosting collaborative projects and workshops between Norway, Sweden and Scotland in 2011-2012.

OATS

We thank the OATS participants and gratefully acknowledge the support and assistance of the OATS Research Team. This work was facilitated by access to the Australian Twin Registry, a national research resource supported by the NHMRC Enabling Grant 310667 and administered by the University of Melbourne. The study is supported by a National Health and Medical Research Council (NHMRC)/Australian Research Council Strategic Award (Grant 401162) and a NHMRC Project Grant (1045325). DNA was extracted by Genetic Repositories Australia, an Enabling Facility, supported by the NHMRC Grant 401184. OATS genotyping was partly funded by a CSIRO Flagship Collaboration Fund Grant. Genome-wide genotyping was performed by the Diamantina Institute, University of Queensland.

ORCADES

The Orkney Complex Disease Study (ORCADES) was supported by the Chief Scientist Office of the Scottish Government (CZB/4/276, CZB/4/710), the Royal Society, the MRC Human Genetics Unit quinquennial programme “QTL in Health and Disease”, Arthritis Research UK and the European Union framework program 6 EUROSPAN project (contract no. LSHG-CT-2006-018947). DNA extractions were performed at the Edinburgh Clinical Research Facility, University of Edinburgh. We would like to acknowledge the invaluable contributions of the research nurses in Orkney, the administrative team in Edinburgh and the people of Orkney.

PROSPER

The PROSPER study was supported by an investigator initiated grant obtained from Bristol-Myers Squibb. Prof. Dr. J. W. Jukema is an Established Clinical Investigator of the Netherlands Heart Foundation (grant 2001 D 032). Support for genotyping was provided by

the seventh framework program of the European commission (grant 223004) and by the Netherlands Genomics Initiative (Netherlands Consortium for Healthy Aging grant 050-060-810).

The Rotterdam Study (RSI, RSII, RSIII)

The Rotterdam Study is funded by Erasmus Medical Center and Erasmus University, Rotterdam, Netherlands Organization for the Health Research and Development (ZonMw), the Research Institute for Diseases in the Elderly (RIDE), the Ministry of Education, Culture and Science, the Ministry for Health, Welfare and Sports, the European Commission (DG XII), and the Municipality of Rotterdam. The authors are grateful to the study participants, the staff from the Rotterdam Study and the participating general practitioners and pharmacists. The generation and management of the Illumina exome chip v1.0 array data for the Rotterdam Study (RS-I) was executed by the Human Genotyping Facility of the Genetic Laboratory of the Department of Internal Medicine (www.glimdna.org), Erasmus MC, Rotterdam, The Netherlands. The generation and management of GWAS genotype data for the Rotterdam Study (RS-I, RS-II, RS-III) was executed by the Human Genotyping Facility of the Genetic Laboratory of the Department of Internal Medicine, Erasmus MC, Rotterdam, The Netherlands. The GWAS datasets are supported by the Netherlands Organization of Scientific Research NWO Investments (nr. 175.010.2005.011, 911-03-012), the Genetic Laboratory of the Department of Internal Medicine, Erasmus MC, the Research Institute for Diseases in the Elderly (014-93-015; RIDE2), the Netherlands Genomics Initiative (NGI)/Netherlands Organization for Scientific Research (NWO) Netherlands Consortium for Healthy Aging (NCHA), project nr. 050-060-810. Carolina Medina-Gomez, PhD, Lennard Karsten, PhD, and Linda Broer, PhD, for QC and variant calling; Arp, Mila Jhamai, Marijn Verkerk, Lizbeth Herrera and Marjolein Peters, PhD, and Carolina Medina-Gomez, PhD, for their help in creating the GWAS database.

SATSA

SATSA was supported by NIH grants R01 AG04563, R01 AG10175, the MacArthur Foundation Research Network on Successful Aging, the Swedish Council For Working Life and Social Research (FAS) (97:0147:1B, 2009-0795) and Swedish Research Council (825-2007-7460, 825-2009-6141); PI: N.L. Pedersen. DNA extraction and genotyping was supported in part by NIH R01 AG028555 (Reynolds) and NIH R01 AG037985 (Pedersen).

Sydney MAS

We thank the Sydney MAS participants and the Sydney MAS Research Team for their support and assistance. The study is supported by a National Health and Medical Research Council (NHMRC)/Australian Research Council Strategic Award (Grant 401162) and NHMRC Program Grants (350833, 568969). DNA was extracted by Genetic Repositories Australia, an Enabling Facility, supported by the NHMRC Grant 401184. Genome-wide genotyping was performed by the Ramaciotti Centre, University of New South Wales.

Understanding Society

These data are from Understanding Society: The UK Household Longitudinal Study, which is led by the Institute for Social and Economic Research at the University of Essex and funded by the Economic and Social Research Council (Grant Number: ES/M008592/1). The data were collected by NatCen and the genome wide scan data were analysed by the Wellcome Trust Sanger Institute. Information on how to access the data can be found on the Understanding Society website https://www.understandingsociety.ac.uk/

Data governance was provided by the METADAC data access committee, funded by ESRC, Wellcome, and MRC. (Grant Number: MR/N01104X/1)

VETSA

William Kremen and Michael Lyons are Principal Investigators on the Vietnam Era Twin Study of Aging (VETSA), which is funded by the US National Institute on Aging (AG050595, AG022381). SNP genotyping for VETSA was performed by deCODE Genetics, Reykjavik, Iceland.

YFS

The Young Finns Study has been financially supported by the Academy of Finland: grants 286284, 134309 (Eye), 126925, 121584, 124282, 129378 (Salve), 117787 (Gendi), and 41071 (Skidi); the Social Insurance Institution of Finland; Competitive State Research Financing of the Expert Responsibility area of Kuopio, Tampere and Turku University Hospitals (grant X51001); Juho Vainio Foundation; Paavo Nurmi Foundation; Finnish Foundation for Cardiovascular Research; Finnish Cultural Foundation; Tampere Tuberculosis Foundation; Emil Aaltonen Foundation; Yrjö Jahnsson Foundation; Signe and Ane Gyllenberg Foundation; and Diabetes Research Foundation of Finnish Diabetes Association.

Sources of Genetic Results from Genome-wide Association Consortia

The following consortia provided summary data which were used in downstream analyses.

CARDIoGRAM. Coronary artery disease data have been contributed by CARDIoGRAMplusC4D investigators.

CHARGE-Aging and Longevity. Longevity data have been provided by the CHARGE-Aging and Longevity consortium. Longevity was defined as reaching age 90 years or older. Genotyped participants who died between the ages of 55 and 80 years were used as the control group. There were 6,036 participants who achieved longevity and 3757 participants in the control group across participating studies in the discovery meta-analysis.

Broer L, Buchman AS, Deelen J, Evans DS, Faul JD, Lunetta KL, Sebastiani P, Smith JA, Smith AV, Tanaka T, Yu L, Arnold AM, Aspelund T, Benjamin EJ, De Jager PL, Eirkisdottir G, Evans DA, Garcia ME, Hofman A, Kaplan RC, Kardia SL, Kiel DP, Oostra BA, Orwoll ES, Parimi N, Psaty BM, Rivadeneira F, Rotter JI, Seshadri S, Singleton A, Tiemeier H, Uitterlinden AG, Zhao W, Bandinelli S, Bennett DA, Ferrucci L, Gudnason V, Harris TB, Karasik D, Launer LJ, Perls TT, Slagboom PE, Tranah GJ, Weir DR, Newman AB, van Duijn CM and Murabito JM. GWAS of Longevity in CHARGE Consortium Confirms APOE and FOXO3 Candidacy. J Gerontol A Biol Sci Med Sci. 2015;70:110-8.

The CHARGE Aging and Longevity working group analysis of the longevity phenotype was funded through the individual contributing studies. The working group thanks all study participants and study staff.

DIAGRAM. Type 2 diabetes data were obtained from the DIAGRAM consortium.

ENIGMA. Brain imaging data were obtained from the ENIGMA consortium.

GIANT. BMI data were obtained from the GIANT consortium.

International Genomics of Alzheimer's Project (IGAP). Alzheimer's disease data were obtained from (IGAP).

International Genomics of Alzheimer's Project (IGAP) is a large two-stage study based upon genome-wide association studies (GWAS) on individuals of European ancestry. In stage 1, IGAP used genotyped and imputed data on 7,055,881 single nucleotide polymorphisms (SNPs) to meta-analyse four previously-published GWAS datasets consisting of 17,008 Alzheimer's disease cases and 37,154 controls (The European Alzheimer's disease Initiative – EADI, the Alzheimer Disease Genetics Consortium – ADGC, The Cohorts for Heart and Aging Research in Genomic Epidemiology consortium – CHARGE, The Genetic and Environmental Risk in AD consortium – GERAD). In stage 2, 11,632 SNPs were genotyped and tested for association in an independent set of 8572 Alzheimer's disease cases and 11,312 controls. Finally, a meta-analysis was performed combining results from stages 1 & 2.

We thank the International Genomics of Alzheimer's Project (IGAP) for providing summary results data for these analyses. The investigators within IGAP contributed to the design and implementation of IGAP and/or provided data but did not participate in analysis or writing of this report. IGAP was made possible by the generous participation of the control subjects, the patients, and their families. The i–Select chips was funded by the French National Foundation on Alzheimer's disease and related disorders. EADI was supported by the LABEX (laboratory of excellence program investment for the future) DISTALZ grant, Inserm, Institut Pasteur de Lille, Université de Lille 2 and the Lille University Hospital. GERAD was supported by the Medical Research Council (Grant No. 503480), Alzheimer's Research UK (Grant No. 503176), the Wellcome Trust (Grant No. 082604/2/07/Z) and German Federal Ministry of Education and Research (BMBF): Competence Network Dementia (CND) grant No. 01GI0102, 01GI0711, 01GI0420. CHARGE was partly supported

by the NIH/NIA grant R01 AG033193 and the NIA AG081220 and AGES contract N01–AG–12100, the NHLBI grant R01 HL105756, the Icelandic Heart Association, and the Erasmus Medical Center and Erasmus University. ADGC was supported by the NIH/NIA grants: U01 AG032984, U24 AG021886, U01 AG016976, and the Alzheimer's Association grant ADGC–10–196728.

Psychiatric Genetics Consortium. Schizophrenia, bipolar disorder, and ADHD were obtained from the Psychiatric Genetics Consortium.

LD Hub. Lung cancer, Autism spectrum disorder, Birth weight, Depressive symptoms, Hypertension, Pulse wave Arterial Stiffness, Angina, Heart attack, Parental longevity, Forced expiratory volume in 1-second (FEV1), Hand grip strength, Happiness, Health satisfaction, Heel bone mineral density, Osteoarthritis, Overall health rating, Wearing of glasses or contact lenses, Short-sightedness, Long-sightedness, Sleep duration, Sleeplessness/insomnia, and Subjective wellbeing were obtained via LD Hub.

We gratefully acknowledge all the studies and databases that made GWAS summary data available: ADIPOGen (Adiponectin genetics consortium), C4D (Coronary Artery Disease Genetics Consortium), CARDIoGRAM (Coronary ARtery DIsease Genome wide Replication and Meta-analysis), CKDGen (Chronic Kidney Disease Genetics consortium), dbGAP (database of Genotypes and Phenotypes), DIAGRAM (DIAbetes Genetics Replication And Meta-analysis), ENIGMA (Enhancing Neuro Imaging Genetics through Meta Analysis), EAGLE (EArly Genetics & Lifecourse Epidemiology Eczema Consortium, excluding 23andMe), EGG (Early Growth Genetics Consortium), GABRIEL (A Multidisciplinary Study to Identify the Genetic and Environmental Causes of Asthma in the European Community), GCAN (Genetic Consortium for Anorexia Nervosa), GEFOS (GEnetic Factors for OSteoporosis Consortium), GIANT (Genetic Investigation of ANthropometric Traits),

GIS (Genetics of Iron Status consortium), GLGC (Global Lipids Genetics Consortium), GPC (Genetics of Personality Consortium), GUGC (Global Urate and Gout consortium), HaemGen (haemotological and platelet traits genetics consortium), HRgene (Heart Rate consortium), IIBDGC (International Inflammatory Bowel Disease Genetics Consortium), ILCCO (International Lung Cancer Consortium), IMSGC (International Multiple Sclerosis Genetic Consortium), MAGIC (Meta-Analyses of Glucose and Insulin-related traits Consortium), MESA (Multi-Ethnic Study of Atherosclerosis), PGC (Psychiatric Genomics Consortium), Project MinE consortium, ReproGen (Reproductive Genetics Consortium), SSGAC (Social Science Genetics Association Consortium) and TAG (Tobacco and Genetics Consortium), TRICL (Transdisciplinary Research in Cancer of the Lung consortium), UK Biobank.

We gratefully acknowledge the contributions of Alkes Price (the systemic lupus erythematosus GWAS and primary biliary cirrhosis GWAS) and Johannes Kettunen (lipids metabolites GWAS).

Supplementary Figures

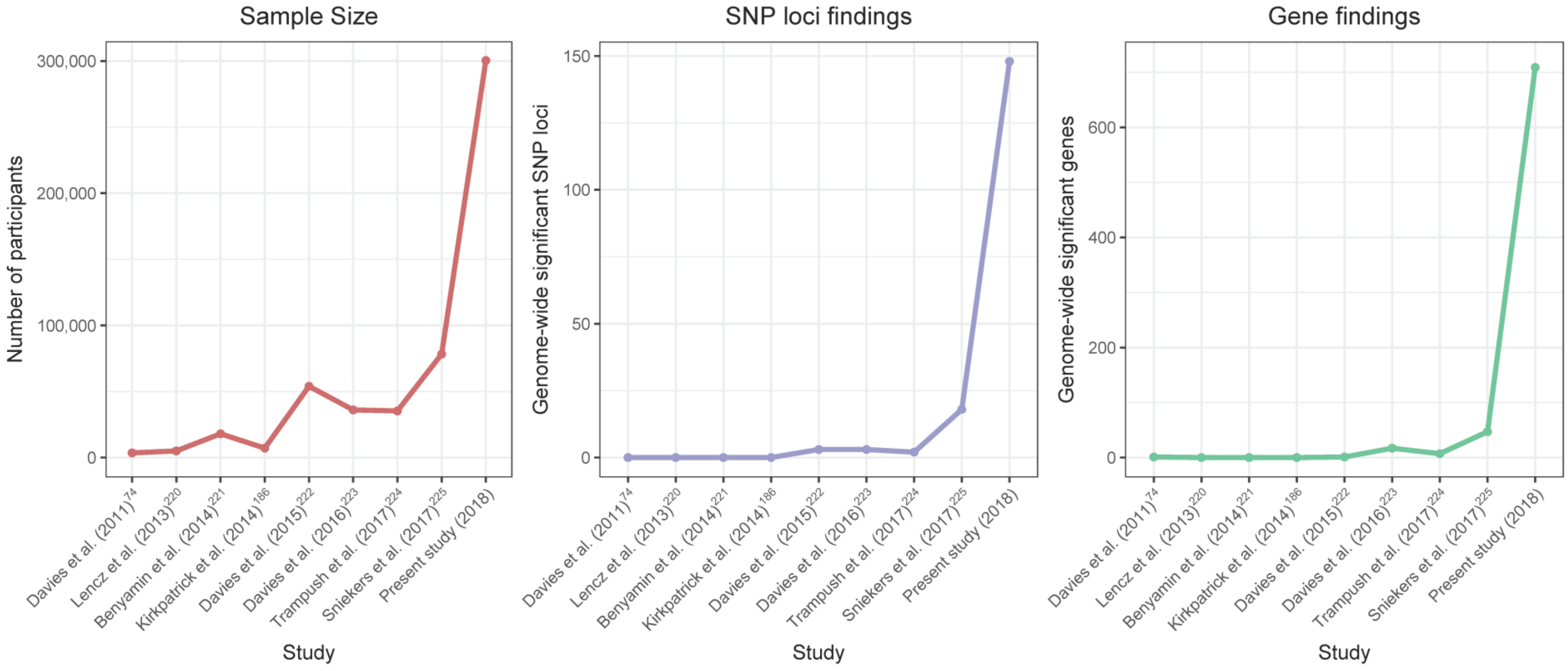

Supplementary Figure 1 Summary of molecular genetic association studies with general cognitive function to date.

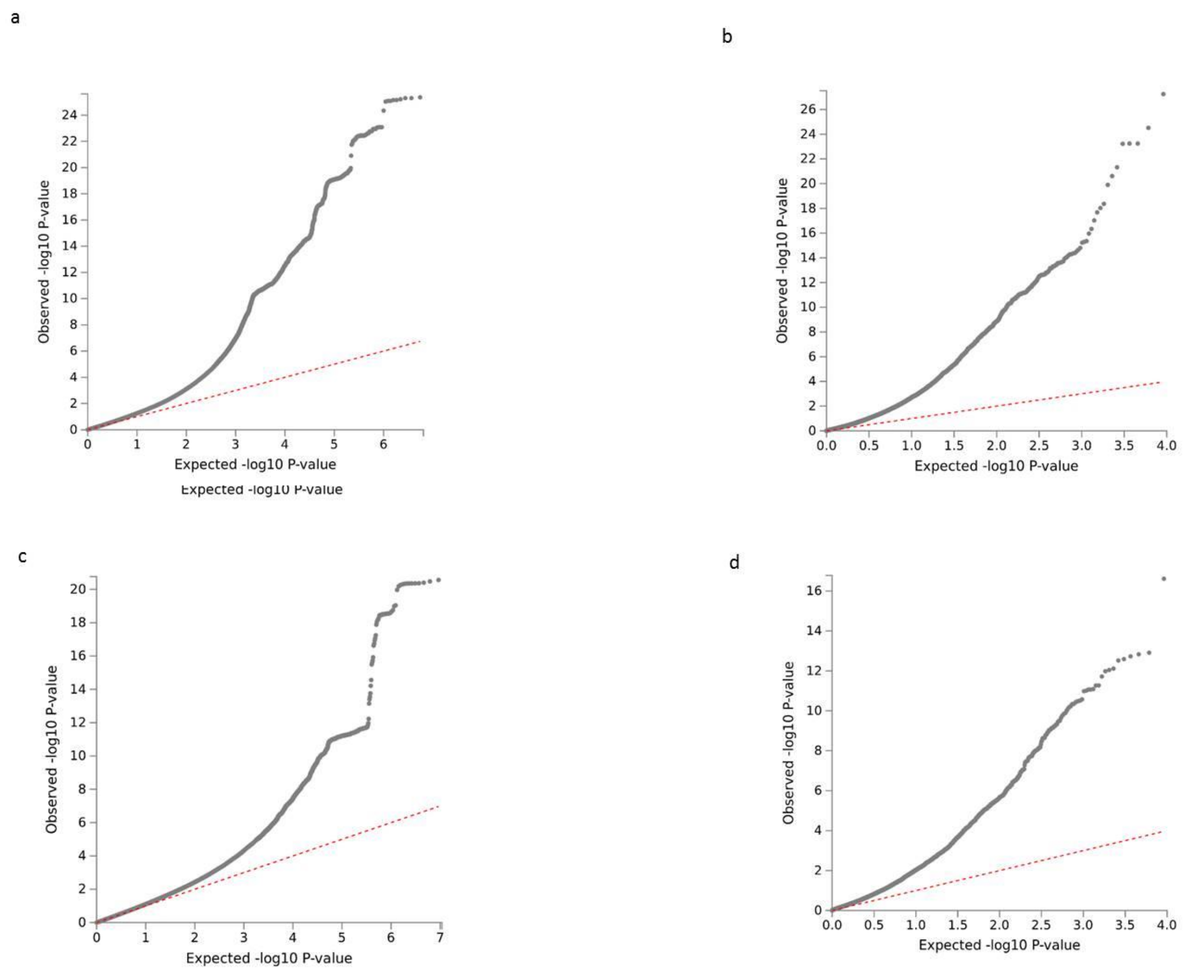

Supplementary Figure 2 Quantile-quantile plots for a) general cognitive function GWAS, b) general cognitive function gene-based analysis, c) reaction time GWAS, d) reaction time gene-based analysis.

Supplementary Tables

Tissue group	Number of genes	Beta	SE	P
**Brain**	**16778**	**0.0942**	**0.0115**	**1.43E-16**
**Pituitary**	**16778**	**0.0838**	**0.0147**	**6.40E-09**
Testis	16778	0.0225	0.00891	0.0057259
Ovary	16778	0.0457	0.0203	0.012051
Uterus	16778	0.0512	0.0274	0.030849
Nerve	16778	0.0281	0.0227	0.10825
Colon	16778	0.0114	0.0398	0.3877
Cervix Uteri	16778	0.00986	0.0417	0.40648
Blood Vessel	16778	-0.00132	0.0251	0.52097
Adrenal Gland	16778	-0.00413	0.0195	0.58384
Blood	16778	-0.00265	0.0104	0.60017
Fallopian Tube	16778	-0.00986	0.0353	0.61006
Stomach	16778	-0.012	0.0306	0.6518
Prostate	16778	-0.0138	0.0332	0.66166
Muscle	16778	-0.00532	0.0115	0.67848
Bladder	16778	-0.0239	0.0353	0.75046
Pancreas	16778	-0.0111	0.0164	0.75164
Esophagus	16778	-0.0421	0.0418	0.84337
Small Intestine	16778	-0.0203	0.0181	0.86915
Vagina	16778	-0.034	0.0296	0.87484
Skin	16778	-0.0284	0.0219	0.90239
Heart	16778	-0.0295	0.0171	0.95803
Adipose Tissue	16778	-0.0537	0.0297	0.96474
Thyroid	16778	-0.0468	0.0244	0.97219
Kidney	16778	-0.0388	0.0202	0.97252
Liver	16778	-0.0206	0.0107	0.97257
Spleen	16778	-0.0284	0.0142	0.97709
Breast	16778	-0.0837	0.0412	0.97896
Lung	16778	-0.0619	0.0232	0.99622
Salivary Gland	16778	-0.0655	0.0227	0.99809

Supplementary Table 1. MAGMA gene-property analysis for general cognitive function. 30 general tissue types were created using gene expression data based on GTEx RNA-seq data. Tissue groupings that withstood Bonferroni are highlighted in bold.

	threshold	General cognitive function beta	se	r2 %	p	Reaction time beta	se	r2 %	p
**ELSA**	0.01	0.156	0.012	2.06	**$1.61×10^{-40}$**	-0.055	0.011	0.3	**$3.59×10^{-7}$**
	0.05	0.169	0.011	2.54	**$8.09×10^{-50}$**	-0.052	0.011	0.27	**$1.37×10^{-6}$**
	0.1	0.167	0.011	2.52	**$1.99×10^{-49}$**	-0.06	0.011	0.36	**$2.91×10^{-8}$**
	0.5	0.168	0.011	2.63	**$1.70×10^{-51}$**	-0.066	0.011	0.43	**$1.42×10^{-9}$**
	1	0.166	0.011	2.59	**$1.20×10^{-50}$**	-0.065	0.011	0.42	**$2.35×10^{-9}$**
**Generation Scotland**	0.01	0.197	0.012	3.31	**$2.49×10^{-60}$**	-0.074	0.011	0.55	**$3.64×10^{-11}$**
	0.05	0.204	0.012	3.71	**$1.08×10^{-67}$**	-0.074	0.011	0.54	**$5.23×10^{-11}$**
	0.1	0.204	0.012	3.73	**$5.02×10^{-68}$**	-0.072	0.011	0.52	**$1.34×10^{-10}$**
	0.5	0.198	0.011	3.59	**$2.00×10^{-65}$**	-0.072	0.011	0.52	**$1.29×10^{-10}$**
	1	0.198	0.011	3.59	**$1.59×10^{-65}$**	-0.075	0.011	0.56	**$2.49×10^{-11}$**
**Understanding Society**	0.01	0.198	0.011	3.24	**$3.74×10^{-66}$**	-0.04	0.011	0.15	**0.00021**
	0.05	0.219	0.011	4.12	**$3.29×10^{-84}$**	-0.051	0.011	0.26	**$1.61×10^{-6}$**
	0.1	0.222	0.011	4.31	**$6.17×10^{-88}$**	-0.05	0.011	0.25	**$2.62×10^{-6}$**
	0.5	0.217	0.011	4.23	**$1.85×10^{-86}$**	-0.051	0.011	0.26	**$1.50×10^{-6}$**
	1	0.217	0.011	4.21	**$5.45×10^{-86}$**	-0.051	0.011	0.26	**$1.57×10^{-6}$**

Supplementary Table 2. Associations between polygenic profiles based on general cognitive function and reaction time and general cognitive function in ELSA, Generation Scotland and Understanding Society. Significant associations (FDR correct p-value =< 0.00021) are shown in bold. All beta values are standardised.

Tissue group	Genes	Beta	SE	P
**Brain**	**16803**	**0.0659**	**0.00922**	**4.66E-13**
**Pituitary**	**16803**	**0.036**	**0.0114**	**0.00075967**
Ovary	16803	0.0413	0.015	0.0029259
Uterus	16803	0.0345	0.0192	0.036096
Nerve	16803	0.0281	0.0166	0.044747
Testis	16803	0.0106	0.0073	0.074192
Blood	16803	0.00536	0.00863	0.2673
Colon	16803	0.0163	0.0269	0.27224
Thyroid	16803	0.00374	0.0174	0.41498
Skin	16803	0.00152	0.016	0.46205
Muscle	16803	0.000574	0.00937	0.47559
Adrenal gland	16803	-0.00161	0.0147	0.54367
Cervix uteri	16803	-0.00348	0.0273	0.5507
Esophagus	16803	-0.00756	0.0282	0.60555
Prostate	16803	-0.00787	0.0222	0.63854
Stomach	16803	-0.00833	0.022	0.64737
Bladder	16803	-0.00959	0.0238	0.65642
Spleen	16803	-0.00605	0.0107	0.71344
Fallopian tube	16803	-0.014	0.0239	0.72152
Blood vessel	16803	-0.0125	0.0179	0.75659
Heart	16803	-0.0094	0.0133	0.76087
Vagina	16803	-0.0187	0.0208	0.81623
Small intestine	16803	-0.0186	0.0138	0.91219
Adipose tissue	16803	-0.045	0.0206	0.98547
Liver	16803	-0.0218	0.00893	0.99265
Kidney	16803	-0.0374	0.0151	0.99351
Lung	16803	-0.0424	0.0165	0.9949
Pancreas	16803	-0.0354	0.0131	0.99668
Salivary gland	16803	-0.0471	0.0165	0.99785
Breast	16803	-0.0801	0.0273	0.99831

Supplementary Table 3. MAGMA gene-property analysis for reaction time. 30 general tissue types were created using gene expression data based on GTEx RNA-seq data. Tissue groupings that withstood Bonferroni are highlighted in bold.

Supplementary References

1. Lyall, D. M. et al. Cognitive test scores in UK Biobank: data reduction in 480,416 participants and longitudinal stability in 20,346 participants. *PloS One* doi:10.1371/journal.pone.0154222 (2016).
2. Hagenaars, S. P. et al. Shared genetic aetiology between cognitive functions and physical and mental health in UK Biobank (N= 112 151) and 24 GWAS consortia. *Mol. Psychiatry **21***, 1624-1632 (2016).
3. The 3C Study Group. Vascular factors and risk of dementia: design of the Three-City Study and baseline characteristics of the study population. *Neuroepidemiology **22**,* 316–325 (2003).
4. Wolfsgruber, S. et al. The CERAD neuropsychological assessment battery total score detects and predicts Alzheimer disease dementia with high diagnostic accuracy. *Am. J. Geriatr. Psychiatry* **22**, 1017–1028 (2014).
5. Wechsler, D. *WAIS-IIIUK administration and scoring manual*. (Psychological Corporation: London, 1998).
6. Delis, D.C., Kramer, J.H., Kaplan, E., & Ober, B.A. *California Verbal Learning Test: Adult Version Manual*. (The Psychological Corporation: San Antonio, TX, 1987).
7. Salthouse, T., & Babcock, R. Decomposing adult age differences in executive function. *Dev. Psychol.* **27**, 763–776 (1991).
8. Spreen, O., & Strauss, E. *A compendium of neuropsychological tests*. (Oxford University Press: New York, 1991).
9. Sweeney, J. A., Kmiec, J. A., & Kupfer, D. J. Neuropsychologic impairments in bipolar and unipolar mood disorders on the CANTAB neurocognitive battery. *Biol. Psychiatry* **48**, 674–684 (2000).

10. Saczynski, J. S. et al. Cognitive impairment: an increasingly important complication of type 2 diabetes: the age, gene/environment susceptibility–Reykjavik study. *Am. J. Epidemiol.* **168**, 1132–1139 (2008).

11. Lezak, M. *Neuropsychological Assessment 3rd Ed*. (Oxford: Oxford University Press, 1995).

12. Knopman, D. S., & Ryberg, S. A verbal memory test with high predictive accuracy for dementia of the Alzheimer type. *Arch. Neurol.* **46**, 141–145 (1989).

13. Wechsler, D. *WAIS-R Manual*. (Psychological Corporation, San Antonio, TX, 1981).

14. Gatterer, G. *Alters-Konzentrations-Test (AKT)*. (Verlag für Psychologie Hogrefe, Göttingen, Germany, 1990).

15. Bäumler, G. *Lern-und Gedächtnistest: LGT-3*. (Dr CJ Hogrefe Verlag Psychologie, Göttingen, Germany, 1974).

16. Reaktionsgerät, W. *Grundprogramm*. (Dr. Schuhfried Ges. mbH, Mödling, Austria, 1991).

17. Tewes, U. *Hamburg-Wechsler Intelligenztest für Erwachsene (HAWIE-R)*. (Hogrefe, Göttingen, Germany, 1991).

18. Tiffin, J., & Asher, E. J. The Purdue Pegboard: norms and studies of reliability and validity. *J. Appl. Psychol*. **32**, 234 (1948).

19. Army individual test battery. *Manual of directions and scoring*. (United States War Department, Adjutant General's office, Washington DC, 1994).

20. Nagel, I.E. et al. Human aging magnifies genetic effects on executive functioning and working memory. *Front. Hum. Neurosci.* doi:10.3389/neuro.09.001.2008 (2008).

21. Lindenberger, U., Mayr, U., & Kliegl, R. Speed and intelligence in old age. *Psychol. Aging* **8**, 207-220 (1993).

22. Vandenberg, S.G., & Kuse, A.R. Mental rotations, a group test of three-dimensional spatial visualization. *Percept. Mot. Skills*. **47**, 599–604 (1978).

23. Luciano, M., Wright, M.J., Geffen, G.M., Geffen, L.B., Smith, G.A., & Martin, N.G. A genetic investigation of the covariation among inspection time, choice reaction time, and IQ subtest scores. *Behav. Genet.* **34**, 41–50 (2004).

24. Wainwright, M., Wright, M.J., Geffen, G.M., Geffen, L.B., Luciano, M., & Martin, N.G. Genetic and environmental sources of covariance between reading tests used in neuropsychological assessment and IQ subtests. *Behav. Genet*. **34**, 365–376 (2004).

25. Nilsson, L.-G., Adolfsson, R., Bäckman, L., de Frias, C.M., Molander, B., & Nyberg, L. Betula: A prospective cohort study on memory, health and aging. *Aging Neuropsychol. C.* **11**, 134–148 (2004).

26. Larsson, M., Nilsson, L.-G., Olofsson, J.K., & Nordin, S. Demographic and cognitive predictors of cued odor identification: evidence from a population-based study. *Chem. Senses* **29**, 547–554 (2004).

27. Lopez, O.L. et al. Neuropsychological characteristics of mild cognitive impairment subgroups. *J. Neurol. Neurosur. Ps.* **77,** 159–165 (2006).

28. Fitzpatrick, A.L. et al. Incidence and prevalence of dementia in the Cardiovascular Health Study. *J. Am. Geriatr. Soc.* **52**, 195–204 (2004).

29. Raven, J.C., Court, J.H., & Raven, J. *Manual for Raven's Progressive Matrices and Vocabulary Scales*. (H. K. Lewis, London, UK, 1977).

30. Steptoe, A., Breeze, E., Banks, J., & Nazroo, J. Cohort Profile: The English Longitudinal Study of Ageing. *Int. J. Epidemiol.* **42**, 1640–1648 (2013).

31. Cees De Groot, J. et al. Cerebral white matter lesions and cognitive function: the Rotterdam Scan Study. *Ann. Neurol*. **47**, 145–151 (2000).

32. Liu, F. et al. The apolipoprotein E gene and its age-specific effects on cognitive function. *Neurobiol. Aging* **31**, 1831–1833 (2010).

33. Au, R., et al. New norms for a new generation: cognitive performance in the Framingham offspring cohort. *Exp. Aging Res*. **30**, 333–358 (2004).

34. Latvala, A. et al. Genetic origins of the association between verbal ability and alcohol dependence symptoms in young adulthood. *Psychol. Med.* **41**, 641–651 (2011).

35. Vuoksimaa, E., Kaprio, J., Eriksson, C.P., & Rose, R.J. Pubertal testosterone predicts mental rotation performance of young adult males. *Psychoneuroendocrino*. **37**, 1791–1800 (2012).

36. Smith, B.H et al. Cohort Profile: Generation Scotland: Scottish Family Health Study (GS: SFHS). The study, its participants and their potential for genetic research on health and illness. *Int. J. Epidemiol*. **42**, 689–700 (2012).

37. Smith, B.H. et al. Generation Scotland: the Scottish Family Health Study; a new resource for researching genes and heritability. *BMC Med. Genet*. **7**, 74 (2006).

38. Rey, A. *L'Examen Clinique en Psychologie*. (Presses Universitaires de France, 1964).

39. Spreen, O., & Strauss E. *Memory. A Compendium of Neuropsychological Tests: Administration, Norms, and Commentary*. (Oxford University Press, New York, 1998).

40. Benton, A.L. Neuropsychological assessment. *Annu. Rev. Psychol*. **45**, 1–23 (1994).

41. Stroop J. Studies of Inference in Serial Verbal Reactions. *J. Exp. Psycho*. **18**, 643-662 (1935).

42. Golden, C. J. *Stroop color and word test: cat. no. 30150M; a manual for clinical and experimental uses*. (Stoelting Company, Wood Dale, IL, 1978).

43. Gatz, M. et al. Complete ascertainment of dementia in the Swedish Twin Registry: the HARMONY study. *Neurobiol. Aging*. **26**, 439–447 (2005).

44. Schofield, P.W. et al. The Audio Recorded Cognitive Screen (ARCS): a flexible hybrid cognitive test instrument. *J. Neurol. Neurosur. Ps.*, **86**, 602–607 (2010).

45. Crimmins, E.M., Kim, J.K., Langa, K.M., & Weir, D.R. Assessment of cognition using surveys and neuropsychological assessment: the Health and Retirement Study and the Aging, Demographics, and Memory Study. *J. Gerontol. B-Psychol.* **66**, i162–i171 (2011).

46. Ofstedal, M.B., Fisher, G.G., & Herzog, A.R. *Documentation of cognitive functioning measures in the Health and Retirement Study.* (University of Michigan, Ann Arbor, MI, 2005)

47. Deary, I.J., Whiteman, M.C., Starr, J.M., Whalley, L.J., & Fox, H.C. The impact of childhood intelligence on later life: following up the Scottish mental surveys of 1932 and 1947. *J. Pers. Soc. Psychol.* **86**, 130-147 (2004).

48. Deary, I.J., et al. The Lothian Birth Cohort 1936: a study to examine influences on cognitive ageing from age 11 to age 70 and beyond. *BMC Geriatr.* **7**, 28 (2007).

49. Loeffler, M. et al. The LIFE-Adult-Study: objectives and design of a population-based cohort study with 10,000 deeply phenotyped adults in Germany. *BMC Public Health* **15**, 691 (2015).

50. Stein, J. et al. The assessment of changes in cognitive functioning: Age-, education-, and gender-specific reliable change indices for older adults tested on the cerad-np battery: Results of the German study on ageing, cognition, and dementia in primary care patients (AgeCoDe). *Am. J. Geriatr. Psychiatry* **20**, 84–97 (2012).

51. Then, F.S. et al. Association between mental demands at work and cognitive functioning in the general population–results of the health study of the Leipzig research center for civilization diseases (LIFE). *J. Occup. Med. Toxicol.* **9**, 23 (2014).

52. Bennett, D.A., Schneider, J.A., Buchman, A.S., Barnes, L.L., Boyle, P.A., & Wilson, R.S. Overview and Findings from the Rush Memory and Aging Project. *Curr. Alzheimer Res.* **9**, 646-663 (2012).

53. Sachdev, P.S. et al. The Sydney Memory and Ageing Study (MAS): methodology and baseline medical and neuropsychiatric characteristics of an elderly epidemiological non-demented cohort of Australians aged 70–90 years. *Int. Psychogeriatr.* **22**, 1248–1264 (2010).

54. Sachdev, P.S. et al. Cognitive functioning in older twins: the Older Australian Twins Study. *Australas. J. Ageing* **30**, 17–23 (2011).

55. Wechsler, D. *Wechsler Adult Intelligence Scale-Third Edition (WAIS-III). Stockholm Pearson Assessment*. (The Psychological Corporation, San Antonio, 1997).

56. Wechsler, D. *WMS-III: Technical Manual.* (The Psychological Corporation, San Antonio, 1997).

57. Lezak, M. *Neuropsychological Assessment 4th Ed*. (Oxford University Press, Oxford, 2004).

58. Houx, P. J. et al. Testing cognitive function in elderly populations: the PROSPER study. *J. Neurol. Neurosur. Ps.* **73**, 385–389 (2002).

59. Bennett, D.A., Schneider, J.A., Arvanitakis, Z., & Wilson, R.S. Overview and findings from the religious orders study. *Curr. Alzheimer Res.* **9**, 628-645 (2012).

60. Hoogendam, Y.Y., Hofman, A., van der Geest, J.N., van der Lugt, A., & Ikram, M.A. Patterns of cognitive function in aging: the Rotterdam Study. *Eur. J. Epidemiol.* **29**, 133–140 (2014).

61. Pedersen, N.L., Plomin, R., Nesselroade, J.R., & McClearn, G.E. A quantitative genetic analysis of cognitive abilities during the second half of the life span. *Psychol. Sci.* **3**, 346–353 (1992).

62. McFall, S. *Understanding Society: UK Household Longitudinal Study: Cognitive Ability Measures*. (Institute for Social and Economic Research, University of Essex, 2013).

63. Bayroff, A.G., & Anderson, A.A. *Development of Armed Forces Qualification Tests 7 and 8 (Technical Research Report 1122).* (US Army Research Institute, Alexandria, VA, 1963).

64. Orme, D.R., Brehm, W., & Ree, M.J. Armed Forces Qualification Test as a measure of premorbid intelligence. *Mil. Psychol*. **13**, 187-179 (2001).

65. Golden, C. J. *Stroop Color and Word Test*. (Muli-Health Systems, 2003).

66. Delis, D.C., Kaplan, E., & Kramer, J.H. *Delis-Kaplan executive function system: Technical manual*. (Psychological Corporation, San Antonio, 2000).

67. Thurstone, L.L. *A factorial study of perception*. (University of Chicago Press, Chicago, 1944).

68. Ekstrom, R.B., French, J.W., Harman, H.H., & Dermen, D. *Manual for kit of factor-referenced cognitive tests*. (Educational Testing Service, Princeton, NJ, 1976)

69. Kremen, W.S. et al. Cognition in midlife and beyond: The Vietnam Era Twin Study of Aging (VETSA). *Gerontologist* **44** 188 (2004).

70. Franz, C.E. et al. A 35-year longitudinal assessment of cognition and midlife depression symptoms: the Vietnam Era Twin Study of Aging. *Am. J. Geriat. Psychiat*. **19**, 559–570 (2011).

71. Smith, P.J., Need, A.C., Cirulli, E.T., Chiba-Falek, O., & Attix, D.K. A comparison of the Cambridge Automated Neuropsychological Test Battery (CANTAB) with “traditional” neuropsychological testing instruments. *J. Clin. Exp. Neuropsyc.* **35**, 319–328 (2013).

72. De Luca, C. R. et al. Normative data from the CANTAB. I: development of executive function over the lifespan. *J. Clin. Exp. Neuropsyc*. **25**, 242–254 (2003).

73. Lowe, C., & Rabbitt, P. Test re-test reliability of the CANTAB and ISPOCD neuropsychological batteries: theoretical and practical issues. *Neuropsychologia*, **36**, 915–923 (1998).

74. Davies, G. et al. Genome-wide association studies establish that human intelligence is highly heritable and polygenic. *Mol. Psychiatry*, **16**, 996-1005 (2011).

75. Rabbitt, P.M.A. et al. The University of Manchester longitudinal study of cognition in normal healthy old age, 1983 through 2003. *Aging Neuropsychol. C.* **11**, 245–279 (2004).

76. Martinez-Mir, A. et al. Genetic study of neurexin and neuroligin genes in Alzheimer's disease. *J. Alzheimers Dis.* **35**, 403–412 (2013).

77. Morra, J.H. et al. Automated 3D mapping of hippocampal atrophy and its clinical correlates in 400 subjects with Alzheimer's disease, mild cognitive impairment, and elderly controls. *Hum. Brain. Mapp*. **30**, 2766–2788 (2009).

78. Mueller, S. G. et al. Ways toward an early diagnosis in Alzheimer's disease: the Alzheimer's Disease Neuroimaging Initiative (ADNI). *Alzheimers Dement.* **1**, 55–66 (2005).

79. Smyrnis, N., Avramopoulos, D., Evdokimidis, I., Stefanis, C.N., Tsekou, H., & Stefanis, N.C. Effect of schizotypy on cognitive performance and its tuning by COMT val 158 Met genotype variations in a large population of young men. *Biol. Psychiat*. **61**, 845–853 (2007).

80. Holdnack H. A. *Wechsler Test of Adult Reading: WTAR*. (The Psychological Corporation, San Antonio, TX, 2001).

81. US Army. Army Individual Test Battery. In *Manual of Directions and Scoring*. (War Department, Adjutant General's Office, Washington DC, 1944).

82. Benton, A., Hamsher, K., & Sivan, A. *Multilingual Aphasia Examination: Manual of Instructions.* (AJA Associates Inc, Iowa City, 1978).

83. Golden, C. The measurement of creativity by the Stroop color and word test. *J. Pers. Assess.* **39**, 502–506 (1975).

84. Green, P. *Story Recall Test.* (Green's Publishing, Edmonton, 2005).

85. Need, A.C. et al. A genome-wide study of common SNPs and CNVs in cognitive performance in the CANTAB. *Hum. Mol. Genet*. **18**, 4650–4661 (2009).

86. Dickinson, D. et al. Differential effects of common variants in SCN2A on general cognitive ability, brain physiology, and messenger RNA expression in schizophrenia cases and control individuals. *JAMA Psychiat*. **71**, 647-656 (2014).

87. Dickinson, D., Goldberg, T.E., Gold, J.M., Elvevåg, B., & Weinberger, D.R. Cognitive factor structure and invariance in people with schizophrenia, their unaffected siblings, and controls. *Schizophrenia Bull.* **37**, 1157–1167 (2011).

88. Lucas, J.A. et al. Normative data for the Mattis Dementia Rating Scale. *J. Clin. Exp. Neuropsyc.* **20**, 536–547 (1998).

89. Schmidt, R et al. The Mattis Dementia Rating Scale: normative data from 1,001 healthy volunteers. *Neurology* **44**, 964–966 (1994).

90. Smith, G.E., Ivnik, R.J., Malec, J.F., Kokmen, E., Tangalos, E., & Petersen, R.C. Psychometric Properties of the Mattis Dementia Rating Scale. *Assessment* **1**, 123-132 (1994).

91. Folstein, M. F., Folstein, S. E., & McHugh, P. R. "Mini-mental state": a practical method for grading the cognitive state of patients for the clinician. *J. Psychiat. Res.* **12**, 189–198 (1975).

92. Battersby, W.S., Bender, M.B., Pollack, M., & Kahn, R.L. Unilateral spatial agnosia (inattention) in patients with cerebral lesions. *Brain* **79**, 68–93 (1956).

93. Tiihonen, J. et al. Premorbid intellectual functioning in bipolar disorder and schizophrenia: results from a cohort study of male conscripts. *Am. J. Psychiat*. **162**, 1904–1910 (2005).

94. Rikkönen, K. et al. Infant growth and hostility in adult life. *Psychosom. Med*. **70**, 306–313 (2008).

95. Barker, D.J.P., Osmond, C., Forsén, T.J., Kajantie, E., & Eriksson, J.G. Trajectories of growth among children who have coronary events as adults. *New Engl. J. Med*. **353**, 1802–1809 (2005).

96. Eriksson, J.G., Osmond, C., Kajantie, E., Forsén, T.J., & Barker, D.J.P. Patterns of growth among children who later develop type 2 diabetes or its risk factors. *Diabetologia* **49**, 2853–2858 (2006).

97. Wechsler, D. *Wechsler Intelligence Scale for Children-Third Edition (WISC-III).* (The Psychological Corporation, San Antonio, 1991).

98. Sattler, J. M. *Assessment of Children: Cognitive Applications* (4th ed.). (Jerome M. Sattler, La Mesa, 2001).

99. Barral, S. et al. Cognitive function in families with exceptional survival. *Neurobiol. Aging* **33**, 619.e1-7 (2012).

100. Cosentino, S., Schupf, N., Christensen, K., Andersen, S.L., Newman, A., & Mayeux, R. Reduced prevalence of cognitive impairment in families with exceptional longevity. *JAMA Neurol*. **70**, 867–874 (2013).

101. Lee, J.H., Cheng, R., Graff-Radford, N., Foroud, T., & Mayeux, R. Analyses of the national institute on aging late-onset Alzheimer's disease family study: implication of additional loci. *Arch. Neurol*. **65**, 1518–1526 (2008).

102. Roussos, P., Giakoumaki, S.G., Georgakopoulos, A., Robakis, N.K., & Bitsios, P. The *CACNA1C* and *ANK3* risk alleles impact on affective personality traits and startle

reactivity but not on cognition or gating in healthy males. *Bipolar Disord*. **13**, 250–259 (2011).

103. Sahakian, B.J., & Owen, A.M. Computerized assessment in neuropsychiatry using CANTAB: discussion paper. *J. Roy. Soc. Med*. **85**, 399–402 (1992).

104. Cohen, J. D.et al. Temporal dynamics of brain activation during a working memory task. *Nature* **386**, 604–608 (1997).

105. Nelson, H. E. A modified card sorting test sensitive to frontal lobe defects. Cortex **12**, 313–324 (1976).

106. Bechara, A., Damasio, A.R., Damasio, H., & Anderson, S.W. Insensitivity to future consequences following damage to human prefrontal cortex. *Cognition* **50**, 7–15 (1994).

107. Rujescu, D., Hartmann, A.M., Gonnermann, C., Möller, H.J., & Giegling, I. M129V variation in the prion protein may influence cognitive performance. *Mol. Psychiatry* **8**, 937 (2003).

108. Espeseth, T., Christoforou, A., Lundervold, A.J., Steen, V.M., Le Hellard, S., & Reinvang, I. Imaging and cognitive genetics: the Norwegian Cognitive NeuroGenetics sample. *Twin Res. Hum. Genet.* **15**, 442–452 (2012).

109. Delis, D.C., Kramer, J.H., Kaplan, E., & Ober, B.A. *California Verbal Learning Test (CVLT-II). Norwegian manual supplement.* (Pearson Assessment, Stockholm, 2004).

110. Delis, D.C., Kaplan, E., & Kramer, J. *The Delis-Kaplan Executive Function System (D-KEFS). Norwegian manual.* (Pearson Assessment, Stockholm, 2005).

111. Wechsler, D. *Wechsler Abbreviated Scale of Intelligence (WASI). Norwegian manual supplement.* (Pearson Assessment, Stockholm, 2007).

112. Gur, R.C. et al. Neurocognitive growth charting in psychosis spectrum youths. *JAMA Psychiat*. **71**, 366–374 (2014).

113. Gur, R.C. et al. Age group and sex differences in performance on a computerized neurocognitive battery in children age 8- 21. *Neuropsychology* **26**, 251-265 (2012).

114. Haatveit, B.C., Sundet, K., Hugdahl, K., Ueland, T., Melle, I., & Andreassen, O.A. The validity of d prime as a working memory index: results from the “Bergen n-back task”. *J. Clin. Exp. Neuropsyc.* **32**, 871–880 (2010).

115. Klove, H. Clinical neuropsychology. In F. M. Forster (Ed.), *The Medical Clinics of North America*. (Saunders, New York, 1963).

116. Wechsler, D. *Wechsler Memory Scale-third edition (WMS-III). Norwegian manual. Stockholm Pearson Assessment*. (Pearson Assessment, Stockholm, 2007).

117. Meyers, J.E., & Meyers, K.R. *Rey Complex Figure Test and Recognition Trial: Professional manual.* (Psychological Assessment Resources, Lutz, FL, 1995).

118. Vaskinn, A., & Sundet, K. Estimating premorbid IQ: A Norwegian version of National Adult Reading Test. *Journal of the Norwegian Psychological Association* **38**, 1133–1140 (2001).

119. Kern, R.S. et al. The MATRICS Consensus Cognitive Battery, part 2: co-norming and standardization. *Am. J. Psychiat.* **165**, 214–220 (2008).

120. Nuechterlein, K.H. et al. The MATRICS consensus cognitive battery, part 1: Test selection, reliability, and validity. *Am. J. Psychiat.* **165**, 203–213 (2008).

121. Luck, T. et al. Mild cognitive impairment in general practice: age-specific prevalence and correlate results from the German study on ageing, cognition and dementia in primary care patients (AgeCoDe). *Dement. Geriatr. Cogn.* **24**, 307–316 (2007).

122. Harris, T.B. et al. Age, gene/environment susceptibility–Reykjavik Study: multidisciplinary applied phenomics. *Am. J. Epidemiol.* **165**, 1076–1087 (2007).

123. The Aric Investigators. The Atherosclerosis Risk in Communities (ARIC) study: design and objectives. *Am. J. Epidemiol.* **129**, 687–702 (1989).

124. Rosamond, W.D. et al. Stroke incidence and survival among middle-aged adults: 9-year follow-up of the Atherosclerosis Risk in Communities (ARIC) cohort. *Stroke* **30**, 736–743 (1999).

125. Blair, C.K. et al. *APOE* genotype and cognitive decline in a middle-aged cohort. *Neurology* **64**, 268–276 (2005).

126. Schmidt, R., Fazekas, F., Kapeller, P., Schmidt, H., & Hartung, H.-P. MRI white matter hyperintensities: Three-year follow-up of the Austrian Stroke Prevention Study. *Neurology* **53**, 132–132 (1999).

127. Schmidt, R. et al. Assessment of cerebrovascular risk profiles in healthy persons: definition of research goals and the Austrian Stroke Prevention Study (ASPS). *Neuroepidemiology* **13**, 308–313 (1994).

128. Seiler, S. et al. Magnetization transfer ratio relates to cognitive impairment in normal elderly. *Front. Aging Neurosci*. **6**, 263 (2014).

129. Ghadery, C. et al. R2* mapping for brain iron: associations with cognition in normal aging. *Neurobiol. Aging* **36**, 925–932 (2015).

130. Hatzimanolis, A. et al. Common genetic variation and schizophrenia polygenic risk influence neurocognitive performance in young adulthood. *Am. J. Med. Genet. B* **168**, 392–401 (2015).

131. Stefanis, N.C. et al. Impact of schizophrenia candidate genes on schizotypy and cognitive endophenotypes at the population level. *Biol. Psychiat.* **62**, 784–792 (2007).

132. Bertram, L. et al. Cohort profile: the Berlin aging study II (BASE-II). *Int. J. Epidemiol.* **43**, 703–712 (2013).

133. Li, S.-C. et al. Aging magnifies the effects of dopamine transporter and D2 receptor genes on backward serial memory. *Neurobiol. Aging* **34**, 358e.1-10 (2013).

134. Papenberg, G. et al. Dopaminergic gene polymorphisms affect long-term forgetting in old age: further support for the magnification hypothesis. *J. Cognitive Neurosci*. **25**, 571–579 (2013).

135. Oldfield, R.C. The assessment and analysis of handedness: the Edinburgh inventory. *Neuropsychologia* **9**, 97–113 (1971).

136. Wright, M. J., & Martin, N. G. The Brisbane Adolescent Twin Study: outline of study methods and research projects. *Aust. J. Psychol*. **56**, 65-78 (2004).

137. Luciano, M. et al. A genetic two-factor model of the covariation among a subset of Multidimensional Aptitude Battery and Wechsler Adult Intelligence Scale—Revised subtests. *Intelligence* **31**, 589–605 (2003).

138. Chiang, M.-C. et al. Genetics of brain fiber architecture and intellectual performance. *J. Neurosci*. **29**, 2212–2224 (2009).

139. Medland, S.E. et al. Common variants in the trichohyalin gene are associated with straight hair in Europeans. *Am. J. Hum. Genet.* **85**, 750–755 (2009).

140. Nilsson, L.-G et al. The Betula prospective cohort study: Memory, health, and aging. *Aging Neuropsychol. C.* **4**, 1–32 (1997).

141. Fried, L.P. et al. The cardiovascular health study: design and rationale. *Ann. Epidemiol.* **1**, 263–276 (1991).

142. Longstreth, W. T. et al. Frequency and predictors of stroke death in 5,888 participants in the Cardiovascular Health Study. *Neurology* **56**, 368–375 (2001).

143. Lopez, O. L., Kuller, L. H., Fitzpatrick, A., Ives, D., Becker, J. T., & Beauchamp, N. Evaluation of dementia in the cardiovascular health cognition study. *Neuroepidemiology* **22**, 1–12 (2003).

144. First, M.B., Spitzer, R.L., Gibbon, M., & Williams, J.B. *Structured Clinical Interview for DSM-IV Axis I Disorders–Non-Patient Edition*. (New York Biometrics Research Dept, New York State Psychiatric Institute, 1995).

145. Voineskos, A.N. et al. Age-related decline in white matter tract integrity and cognitive performance: a DTI tractography and structural equation modeling study. *Neurobiol. Aging* **33**, 21–34 (2012).

146. Lett, T.A. et al. Prefrontal white matter structure mediates the influence of *GAD1* on working memory. *Neuropsychopharmacol.* **41**, 2224–2231 (2016).

147. Rudan, I., Campbell, H., & Rudan, P. Genetic epidemiological studies of Eastern Adriatic Island Isolates, Croatia: Objectives and Strategies. *Collegium Antropol.* **23**, 531–546 (1999).

148. Rudan, I. et al. Effects of inbreeding, endogamy, genetic admixture, and outbreeding on human health: a "1001 Dalmatians" study. *Croat. Med. J.* **47**, 601–610 (2006).

149. Campbell, H. et al. Effects of genome-wide heterozygosity on a range of biomedically relevant human quantitative traits. *Hum. Mol. Genet.* **16**, 233–241 (2007).

150. Vitart, V. et al. Heritabilities of ocular biometrical traits in two croatian isolates with extended pedigrees. *Invest. Ophth. Vis. Sci.* **51**, 737–743 (2010).

151. Vitart, V. et al. *SLC2A9* is a newly identified urate transporter influencing serum urate concentration, urate excretion and gout. *Nat. Genet.* **40**, 437-442 (2008).

152. Polašek, O. et al. Genome-wide association study of anthropometric traits in Korčula Island, Croatia. *Croat. Med. J.* **50**, 7–16 (2009).

153. Cirulli, E.T. et al. Common genetic variation and performance on standardized cognitive tests. *Eur. J. Hum. Genet.* **18**, 815-820 (2010).

154. Nasreddine, Z.S. et al. The Montreal Cognitive Assessment, MoCA: a brief screening tool for mild cognitive impairment. *J. Am. Geriatr. Soc.* **53**, 695–699 (2005).

155. Nikolova, Y.S. et al. Beyond genotype: serotonin transporter epigenetic modification predicts human brain function. *Nat. Neurosci*. **17**, 1153–1155 (2014).

156. Scult, M.A. et al. A common polymorphism in scn2a predicts general cognitive ability through effects on pfc physiology. *J. Cognitive. Neurosci*. **27**, 1766-74 (2015).

157. Rose, E.J. et al. The miR-137 schizophrenia susceptibility variant rs1625579 does not predict variability in brain volume in a sample of schizophrenic patients and healthy individuals. *Am. J. Med. Genet. B* **165**, 467–471 (2014).

158. Rose, E.J., & Donohoe, G. Brain vs behavior: an effect size comparison of neuroimaging and cognitive studies of genetic risk for schizophrenia. *Schizophrenia Bull*. **39**, 518–526 (2012).

159. Marmot, M., Banks, J., Blundell, R., Lessof, C., & Nazroo, J. *Health, wealth and lifestyles of the older population in England: The 2002 English Longitudinal Study of Ageing*. (Institute for Fiscal Studies: London, 2002).

160. Hofman, A., Boomsma, F., Schalekamp, M.A., & Valkenburg, H.A. Raised blood pressure and plasma noradrenaline concentrations in teenagers and young adults selected from an open population. *Brit. Med. J*. **1**, 1536–1538 (1979).

161. Service, S. et al. Magnitude and distribution of linkage disequilibrium in population isolates and implications for genome-wide association studies. *Nat. Genet*. **38**, 556–560 (2006).

162. Aulchenko, Y. S. et al. Linkage disequilibrium in young genetically isolated Dutch population. *Eur. J. Hum. Genet*. **12**, 527-534 (2004).

163. Dawber, T.R., & Kannel, W.B. The Framingham Study an epidemiological approach to coronary heart disease. *Circulation* **34**, 553–555 (1966).

164. Feinleib, M., Kannel, W.B., Garrison, R.J., McNamara, P.M., & Castelli, W.P. The Framingham offspring study. Design and preliminary data. *Prev. Med.* **4**, 518–525 (1975).

165. Splansky, G.L. et al. The third generation cohort of the National Heart, Lung, and Blood Institute's Framingham Heart Study: design, recruitment, and initial examination. *Am. J. Epidemiol.* **165**, 1328–1335 (2007).

166. Carandang, R. et al. Trends in incidence, lifetime risk, severity, and 30-day mortality of stroke over the past 50 years. *JAMA.* **296**, 2939–2946 (2006).

167. Seshadri, S. et al. The lifetime risk of stroke: estimates from the Framingham Study. *Stroke* **37**, 345–350 (2006).

168. Kaprio, J., Pulkkinen, L., & Rose, R.J. Genetic and environmental factors in health-related behaviors: studies on Finnish twins and twin families. *Twin Res. Hum. Genet.* **5**, 366–371 (2002).

169. Kaprio, J. The Finnish twin cohort study: an update. *Twin Res. Hum. Genet*. **16**, 157-162 (2013).

170. Daniels, P.R. et al. Familial aggregation of hypertension treatment and control in the Genetic Epidemiology Network of Arteriopathy (GENOA) study. *Am. J. Med*. **116**, 676–681 (2004).

171. Kerr, S.M. et al. Pedigree and genotyping quality analyses of over 10,000 DNA samples from the Generation Scotland: Scottish Family Health Study. *BMC Med. Genet.* **14**, 38 (2013).

172. Li, H. et al. Candidate single-nucleotide polymorphisms from a genomewide association study of Alzheimer disease. *Arch. Neurol.* **65**, 45–53 (2008).

173. Hu, X. et al. Meta-analysis for genome-wide association study identifies multiple variants at the *BIN1* locus associated with late-onset Alzheimer's disease. *PloS One* doi:10.1371/journal.pone.0016616 (2011).

174. Gatz, M. et al. Role of genes and environments for explaining Alzheimer disease. *Arch. Gen. Psychiat.* **63**, 168–174 (2006).

175. Hong, M.-G. et al. Genome-wide and gene-based association implicates *FRMD6* in Alzheimer disease. *Hum. Mutat.* **33**, 521–529 (2012).

176. McEvoy, M. et al. Cohort profile: the hunter community study. *Int. J. Epidemiol.* **39**, 1452–1463 (2010).

177. Juster, F.T., & Suzman, R. An overview of the Health and Retirement Study. *J. Hum. Resour.* **30**, S7–S56 (1995).

178. Sonnega, A., Faul, J.D., Ofstedal, M.B., Langa, K.M., Phillips, J.W., & Weir, D.R. Cohort profile: the health and retirement study (HRS). *Int. J. Epidemiol.* **43**, 576–585 (2014).

179. Crimmins, E., Guyer, H., Langa, K., Ofstedal, M.B., Wallace, R., & Weir, D. Documentation of physical measures, anthropometrics and blood pressure in the Health and Retirement Study. *HRS Documentation Report DR-011* **14**, 47–59 (2008).

180. Rhea, S.-A., Gross, A.A., Haberstick, B.C., & Corley, R.P. Colorado twin registry. *Twin Res. Hum. Genet.* **9**, 941–949 (2006).

181. Shi, H. et al. Genetic variants influencing human aging from late-onset Alzheimer's disease (LOAD) genome-wide association studies (GWAS). *Neurobiol. Aging* **33**, 1849.e5-18 (2012).

182. Sheenan, D.V., Lecrubier, Y., Sheenan, K.H., Amorim, P., Janavas, J., & Weiller, E. The Mini-International Neuropsychiatric Interview (M.I.N.I.): the development and

validation of a structured diagnostic psychiatric interview for DSM-IV and ICD-10. *J. Clin. Psychiat.* **59**, 22–57 (1998).

183. Newman, A.B. et al. Health and function of participants in the Long Life Family Study: A comparison with other cohorts. *Aging* **3**, 63-76 (2011).

184. Deary, I.J., Gow, A.J., Pattie, A., & Starr, J.M. Cohort profile: the Lothian Birth Cohorts of 1921 and 1936. *Int. J. Epidemiol.* **41**, 1576–1584 (2012).

185. Chibnik, L.B. et al. CR1 is associated with amyloid plaque burden and age-related cognitive decline. *Ann. Neurol.* **69**, 560–569 (2011).

186. Kirkpatrick, R.M., McGue, M., Iacono, W.G., Miller, M.B., & Basu, S. Results of a "GWAS Plus:" General cognitive ability is substantially heritable and massively polygenic. *PloS One* doi:10.1371/journal.pone.011239 (2014).

187. First, M.B., Spitzer, R.L., Gibbon, M., & Williams, J.B. *Structured Clinical Interview for DSM-IV Axis I Disorders - Patient Edition*. (New York Biometrics Res Dep, New York State Psychiatric Institute, 1995).

188. First, M.B., Spitzer, R.L., Gibbon, M., & Williams, B.W. *Structured Clinical Interview for DSM-IV Axis II Personality Disorders (SCID-II).* (New York Biometrics Res Dep New York State Psychiatric Institute, 1990).

189. Rice, J.P et al. Comparison of direct interview and family history diagnoses of alcohol dependence. *Alcohol Clin. Exp. Res.* **19**, 1018–1023 (1995).

190. Kessler, J., Folstein, S.E., & Denzler, P. *Mini-Mental Status Test, German Version*. (Beltz, Weinheim, 1990)

191. Wittchen, H.U., Zaudig, M., & Fydrich, T. *SCID. Structured Clinical Interview for DSM-IV Axis I and II. Manual*. (Hogrefe, Göttingen, 1997).

192. Ising, M. et al. Genetic effects on information processing speed are moderated by age–converging results from three samples. *Genes Brain Behav.* **13**, 501–507 (2014).

193. First, M.B., & Gibbon, M. The Structured Clinical Interview for DSM-IV Axis I Disorders (SCID-I) and the Structured Clinical Interview for DSM-IV Axis II Disorders (SCID- II). In: *Comprehensive handbook of psychological assessment, Vol.* ***2****: Personality assessment*, 134–143, (2004).

194. Sachdev, P.S. et al. A comprehensive neuropsychiatric study of elderly twins: the Older Australian Twins Study. *Twin Res. Hum. Genet.* **12**, 573-582 (2009).

195. McQuillan, R et al. Runs of homozygosity in European populations. *Am. J. Hum. Genet.* **83**, 359–372 (2008).

196. Roalf, D.R. et al. Within-individual variability in neurocognitive performance: Age-and sex-related differences in children and youths from ages 8 to 21. *Neuropsychology* **28**, 506-518 (2014).

197. Shepherd, J. et al. The design of a prospective study of pravastatin in the elderly at risk (PROSPER). *Am. J. Cardiol.* **84**, 1192–1197 (1999).

198. Shepherd , J. et al. PROspective Study of Pravastatin in the Elderly at Risk. Pravastatin in elderly individuals at risk of vascular disease (PROSPER): a randomised controlled trial. *Lancet* **360**, 1623-1630 (2002).

199. Trompet, S. et al. Replication of LDL GWAs hits in PROSPER/PHASE as validation for future (pharmaco) genetic analyses. *BMC Med. Genet.* doi:10.1186/1471-2350-12-131 (2011).

200. Hofman, A. et al. The Rotterdam Study: 2016 objectives and design update. *Eur. J. Epidemiol.* **30**, 661–708 (2015).

201. Prins, N.D. et al. Cerebral small-vessel disease and decline in information processing speed, executive function and memory. *Brain* **128**, 2034–2041 (2005).

202. Bots, M.L., Looman, S.J., Koudstaal, P. J., Hofman, A., Hoes, A.W., & Grobbee, D.E. Prevalence of stroke in the general population. *Stroke* **27**, 1499–1501 (1996).

203. Hollander, M., Koudstaal, P.J., Bots, M.L., Grobbee, D.E., Hofman, A., & Breteler, M.M.B. Incidence, risk, and case fatality of first ever stroke in the elderly population. The Rotterdam Study. *J. Neurol. Neurosur. Ps.* **74**, 317–321 (2003).

204. Finkel, D., & Pedersen, N.L. Processing speed and longitudinal trajectories of change for cognitive abilities: The Swedish Adoption/Twin Study of Aging. *Aging Neuropsychol. C.* **11**, 325–345 (2004).

205. Gold, CH., et al. Gender and health: a study of older unlike-sex twins. *J. Gerontol. B Psychol. Sci. Soc. Sci*. **57**, S168-176 (2002).

206. Davies, G., et al. A genome-wide association study implicates the APOE locus in nonpathological cognitive ageing. *Mol. Psychiatry* **19**, 76–87 (2014).

207. Bakken, T.E. et al. Association of common genetic variants in GPCPD1 with scaling of visual cortical surface area in humans. *P. Natl. Acad. Sci. USA* **109**, 3985–3990 (2012).

208. Spitzer, R.L. et al. Utility of a new procedure for diagnosing mental disorders in primary care: the PRIME-MD 1000 study. *JAMA* **272**, 1749–1756 (1994).

209. Djurovic, S. et al. A genome-wide association study of bipolar disorder in Norwegian individuals, followed by replication in Icelandic sample. *J. Affect. Disorders* **126**, 312–316 (2010).

210. Athanasiu, L. et al. Gene variants associated with schizophrenia in a Norwegian genome-wide study are replicated in a large European cohort. *J. Psychiat. Res.* **44**, 748–753 (2010).

211. Sudlow, C. et al. UK biobank: an open access resource for identifying the causes of a wide range of complex diseases of middle and old age. *PLoS Med*. doi:10.1371/journal.pmed.1001779 (2015).

212. Bycroft, C. et al. Genome-wide genetic data on ~500,000 UK Biobank participants. Preprint at https://www.biorxiv.org/content/early/2017/07/20/166298 (2017).
213. Wain, L.V. et al. Genome-wide association analyses for lung function and chronic obstructive pulmonary disease identify new loci and potential druggable targets. *Nat. Genet.* **49**, 416-425 (2017).
214. Yang, J., Lee, S.H., Goddard, M.E. & Visscher, P.M. GCTA: a tool for genome-wide complex trait analysis. *Am. J. Hum. Genet.* **88**, 76-82 (2011).
215. Buck, N. & McFall, S. Understanding Society: design overview. *Longitudinal and Life Course Studies* **3**, 5 – 17 (2012).
216. Lynn, P. Sample design for understanding society. *Understanding Society Working Paper Series* (2009).
217. Kremen, W.S., Franz, C.E., & Lyons, M.J. VETSA: the Vietnam era twin study of aging. *Twin Res. Hum. Genet.* **16**, 399–402 (2013).
218. Kremen, W. S. et al. Genes, environment, and time: the Vietnam era twin study of aging (VETSA). *Twin Res. Hum. Genet.* **9**, 1009–1022 (2006).
219. Malhotra, A. K. et al. Association between common variants near the melanocortin 4 receptor gene and severe antipsychotic drug–induced weight gain. *Arch. Gen. Psychiat.* **69**, 904–912 (2012).
220. Lencz, T. et al. Molecular genetic evidence for genetic overlap between general cognitive ability and risk for schizophrenia: A report from the cognitive genomics consortium (COGENT). *Mol. Psychiatry* **19**, 168-174 (2014).
221. Benyamin, B. et al. Childhood intelligence is heritable, highly polygenic and associated with *FNBP1L*. *Mol. Psychiatry* **19**, 253-258 (2014).

222. Davies, G. et al. Genetic contributions to variation in general cognitive function: a meta-analysis of genome-wide association studies in the CHARGE consortium (N= 53 949). *Mol. Psychiatry* **20**, 183-192 (2015).

223. Davies, G. et al. Genome-wide association study of cognitive functions and educational attainment in UK Biobank (N = 112 151). *Mol. Psychiatry* **21**, 758-767 (2016).

224. Trampush, J.W. et al. GWAS meta-analysis reveals novel loci and genetic correlates for general cognitive function: a report from the COGENT consortium. *Mol. Psychiatry* **22**, 336-345 (2017).

225. Sniekers, S. et al. Genome-wide association meta-analyses of 78,308 individuals identifies new loci and genes influencing human intelligence. *Nat. Genet.* **49**, 1107-1112 (2017).
