# Supplementary material for: Study of 300,486 individuals identifies 148 independent genetic loci influencing general cognitive function
Source: Nat Commun. 2018 May 29;9:2098. doi: 10.1038/s41467-018-04362-x (PMC5974083; doi:10.1038/s41467-018-04362-x)
Supplement: Supplementary file 3 — Description of Additional Supplementary Files [file 41467_2018_4362_MOESM3_ESM.pdf]

Description of Additional Supplementary Files

File Name: Supplementary Data 1
Description: Genome-wide significant (P < 5 x 10-8) SNP association results for the GWA meta-analysis of CHARGE-COGENT and UK Biobank general cognitive function. Lead SNPs highlighted in bold. GWA results are also shown for CHARGE, COGENT, UK Bioank and Reaction time.

File Name: Supplementary Data 2
Description: Genome-wide suggestive (1 x 10-5 > P > 5 x 10-8) SNP association results for the GWA meta-analysis of CHARGE-COGENT and UK Biobank general cognitive function.

File Name: Supplementary Data 3
Description: Look-ups of independent significant SNPs and tagged SNPs for general cognitive function with intelligence (Hill et al., 2017), educational attainment (Okbay et al., 2016 GWAS) and cognitive function (Sniekers et al., 2017). Previously reported genome-wide significant findings are shown in red.

File Name: Supplementary Data 4
Description: Independent significant SNPs and SNPs tagged within each identified genomic locus for general cognitive function. Nearest gene was defined based on ANNOVAR annotations showing the most deleterious annotation for each of the tagged SNPs. The functional consequence of genetic variation in each SNP was also defined using ANNOVAR. CADD scores describe how deleterious this genetic variation in each of these SNPs is with higher numbers indicating a more deleterious SNP. RegulomeDB score (RDB score) provides a metric describing the biological evidence indicating that this SNP acts as a regulatory element. RDB scores range from 1a (the most evidence) to 7 (the least evidence). minChrState is the minimum 15-core chromatin state across 127 tissue/cell types. A score <8 indicates an open chromatin state. eqtl=1 indicates evidence that the SNP is an expressed quantitative locus. Novel genomic loci are presented in bold.

File Name: Supplementary Data 5
Description: Independent significant SNPs and tagged SNPs for general cognitive function that have previously been identified at P < 9 × 10-6 in GWAS of diseases and traits listed in the GWAS catalog 2017-08-22 (www.ebi.ac.uk/gwas/).

File Name: Supplementary Data 6
Description: Genome-wide significant (P < 2.75 x 10-6) gene based associations for CHARGE-COGENT and UK Biobank general cognitive function. Novel gene based associations are presented in bold.

File Name: Supplementary Data 7
Description: MAGMA gene-set analysis for general cognitive function. Gene-sets that withstood Bonferroni correction (α=0.05/10894) are highlighted in bold. Abbreviations: GO, Gene ontology; bp, biological processes; cc, cellular components; mf, molecular functions.

File Name: Supplementary Data 8
Description: MAGMA gene-property analysis for general cognitive function. 53 specific tissue types were created using gene expression data based on GTEx RNA-seq data. Tissue groupings that withstood Bonferroni are highlighted in bold.

File Name: Supplementary Data 9
Description: Genetic correlations between general cognitive function, reaction time and 52 health outcomes. Significant genetic correlations (FDR p-value threshold general cognitive function =< 0.0269 reaction time =< 0.008) are highlighted in bold.

File Name: Supplementary Data 10
Description: Genome-wide significant (P < 5 x 10-8) SNP association results for UK Biobank reaction time. Lead SNPs highlighted in bold. GWA results for general cogntive function (GCF) are also shown.

File Name: Supplementary Data 11
Description: Genome-wide suggestive (1 x 10-5 > P > 5 x 10-8) SNP association results for UK Biobank reaction time.

File Name: Supplementary Data 12
Description: Independent significant SNPs and SNPs tagged within each identified genomic locus for reaction time. Nearest gene was defined based on ANNOVAR annotations showing the most deleterious annotation for each of the tagged SNPs. The functional consequence of genetic variation in each SNP was also defined using ANNOVAR. CADD scores describe how deleterious this genetic variation in each of these SNPs is with higher numbers indicating a more deleterious SNP. RegulomeDB score (RDB score) provides a metric descibing the biological evidence indicating that this SNP acts as a regulatory element. RDB scores range from 1a (the most evidence) to 7 (the least evidence). minChrState is the minimum 15-core chromatin state across 127 tissue/cell types. A score <8 indicates an open chromatin state. eqtl=1 indicates evidence that the SNP is an expressed quantitative locus. Novel genomic loci are presented in bold.

File Name: Supplementary Data 13
Description: Genome-wide significant (P < 2.75 x 10-6) gene based associations for UK Biobank reaction time. Novel gene based associations are presented in bold.

File Name: Supplementary Data 14
Description: MAGMA gene-set analysis for reaction time. Abbreviations: GO, Gene ontology; bp, biological processes; cc, cellular components; mf, molecular functions.

File Name: Supplementary Data 15
Description: MAGMA gene-property analysis for reaction time. 53 specific tissue types were created using gene expression data based on GTEx RNA-seq data. Tissue groupings that withstood Bonferroni are highlighted in bold.

File Name: Supplementary Data 16
Description: Overlapping genome-wide significant SNP associations between general cognitive function and reaction time. Lead SNPs from the general cognitive function GWAS are in bold. GCF, general cognitive function; RT, reaction time.

File Name: Supplementary Data 17
Description: Over lapping significant gene-based associations between general cognitive function and reaction time. GCF, general cognitive function; RT, reaction time. Novel gene-based associations for general cognitive function are presented in bold.

File Name: Supplementary Data 18
Description: . Cohort descriptive statistics: The total number of participants (% female) and mean, minimum and maximum age per cohort are indicated. The cohort N presented here is the number of individuals that contributed to the meta-analysis, and so these values may differ to the total cohort N stated in the cohort descriptions (Supplementary Note 2).

File Name: Supplementary Data 19
Description: Cohort specific details of genotyping platforms, quality control and imputation algorithms. PCA: Principal Component Analysis, MDS: Multidimensional Scaling, MAF: Minor Allele Frequency, SNP: Single Nucleotide Polymorphism, HWE: Hardy-Weinberg Equilibrium.

File Name: Supplementary Data 20
Description: Sources of GWA results from consortia investigating psychiatric disease and health.
